# Consistency of Ictal Epileptogenic Foci in Children With Tuberous Sclerosis Complex and Its Clinical Predictors

**DOI:** 10.1097/WNP.0000000000001260

**Published:** 2026-05-20

**Authors:** Carmen Stevering, Willem Otte, Frans Leijten, Mireille Bourez, Louis Wagner, Kees Braun, Floor E. Jansen

**Affiliations:** * Department of Paediatric Neurology, Brain Center UMC Utrecht, Utrecht, the Netherlands;; † Department of Neurology, Brain Center UMC Utrecht, Utrecht, the Netherlands;; ‡ Stichting Epilepsie Instellingen Nederland, Heemstede, the Netherlands;; § Academic Center of Epileptology Kempenhaeghe/Maastricht University Medical Center, Maastricht, the Netherlands; and; || Academic Center of Epileptology Kempenhaeghe/Maastricht University Medical Center, Heeze, the Netherlands.

**Keywords:** Tuberous sclerosis complex, Epilepsy, EEG, Epileptogenic focus, Consistency

## Abstract

**Purpose:**

The course of epilepsy in patients with tuberous sclerosis complex (TSC) is highly variable. Consistency of the epileptogenic zone is considered important in presurgical evaluation. The authors aimed to (1) assess consistency of ictal epileptogenic foci over time in patients with TSC, (2) evaluate concordance between interictal epileptiform abnormalities and ictal epileptogenic foci, and (3) identify determinants of ictal consistency.

**Methods:**

This single-center retrospective, observational study included children with definite TSC and epilepsy with ≥2 ictal presurgical EEGs with an interval ≥1 year. In all EEGs, the regional localization of interictal and ictal epileptiform abnormalities was scored based on the EEG reports. All ictal epileptogenic foci were classified with an “ictal focus consistency score,” according to one of the following subcategories: “single focus, consistent,” “from unclear localization/diffuse or generalized to unifocal,” “≥2 or more foci, each consistent,” “≥2 foci of which at least one is consistent,” “starting multifocal but at last EEG unifocal,” and “multifocal changing otherwise.” The primary end point was unifocal or multifocal consistency of ictal foci. The secondary end point was concordance between ictal epileptogenic foci and localization of interictal epileptiform abnormalities. The third end point involved an exploratory assessment of potential determinants of ictal consistency, including TSC variant, age at epilepsy onset, epileptic spasms, multiple seizure types, interictal multifocality, and tuber load.

**Results:**

The authors included 45 patients with TSC with a median of 3 (interquartile range 2–4) ictal and 8 (interquartile range 5–10) interictal EEGs per patient. Median time between first and last ictal EEG was 28.4 months (19.0–60.3). Ictal foci were consistent over time in 11 patients (24.4%), of whom 9 had 1 consistent ictal focus. Nine other patients had a single consistent ictal focus on the first EEGs, but developed a second focus at the median age of 67.7 months. Of the 13 patients who later underwent surgery, 3 had a single focus, and only 2 of 13 had consistent ictal EEGs. The same three patients with a single focus over time were part of the seven postoperative seizure-free patients. All six nonseizure-free children had ictal multifocality. Interictal and ictal epileptogenic foci were discordant in 42 of 45 patients (93.3%). Exploratory analyses of potential determinants of ictal consistency suggested a negative association with interictal multifocality (risk ratio 0.32, 95% confidence interval 0.06–0.97, *p* = 0.04), which was not significant after adjustment for monitoring duration.

**Conclusions:**

Ictal epileptogenic foci are consistent in only 25% of the patients with TSC. Interictal epileptogenic foci are often discordant with ictal foci. This instability in the ictal epileptogenic focus might be explained by the presence of larger epileptogenic networks in TSC that undergo evolution over time.

**Significance:**

The findings in this study could have important implications for presurgical evaluation in patients with TSC and might help to understand the cause of (late) surgical failure.

## Abstract 2

Supplemental Digital Content is Available in the Text.

Tuberous sclerosis complex (TSC) is an autosomal dominant disease affecting many organ systems, such as the brain, heart, skin, and kidneys. Excessive neuronal proliferation and abnormal migration and differentiation underlie the development of the characteristic brain lesions, such as tubers or dysplastic lesions, subependymal nodules, and giant cell astrocytomas. Tubers are associated with neurologic symptoms as epilepsy, intellectual disability, focal neurologic deficits, and autism spectrum disorders.^1^ Epilepsy is present in up to 85% of individuals with TSC,^2,3^ with onset in the first 3 years of life in >70%^2^ and is drug resistant in the majority.^4^ Epilepsy surgery is often considered in these patients, but the presurgical work-up is challenged by an abundance of tubers as potential epileptogenic lesions.^4^

Epilepsy surgery has proven effective in selected patients with TSC, resulting in seizure freedom in 55% to 65% of operated patients.^5–8^ However, postoperative seizure freedom rates decline over time, to 48% to 57% after 10 years of follow-up.^5^ The cause of late surgical failure often remains unexplained.^9^ A major concern is that the epileptogenic focus may shift from one tuber to another with newly developing foci after resection. A previous study showed, however, that of 15 patients in whom ictal EEGs were studied, a shift of foci over time was only seen in 2 patients.^10^ This center previously studied the evolution of interictal epileptogenic foci in a cohort of 21 patients with TSC, and found that 94% of them had one consistent, dominant interictal focus, although over time the number of interictal foci increased on average with 25% (median follow-up interval 9 years).^11^

Improved understanding of the consistency of ictal and interictal EEG foci is important to optimize surgical candidate selection in TSC. With this study, the authors aimed to describe the natural evolution of ictal epileptogenic foci in children with TSC and the determinants of ictal consistency. In addition, the authors correlated ictal with interictal EEG findings over time because they hypothesized that interictal epileptiform abnormalities would predict the ictal epileptogenic focus.

## METHODS

### Study Population

This retrospective, observational study enrolled children (1) with a confirmed genetic or clinical diagnosis of TSC,^12^ (2) with a diagnosis of epilepsy, (3) with ≥2 ictal EEGs available with an interval of at least 1 year. Patients who underwent epilepsy surgery before their second ictal EEG was performed were excluded. Patients were enrolled from the Dutch TSC Registry, with consent for use of data for scientific research. Patient inclusion took place from 2014 onward and data were extracted in 2024. The Dutch Medical Research Involving Human Subjects Act did not apply, as confirmed by the ethical committee of the University Medical Center in Utrecht.

### EEG Evaluation

All available EEGs were evaluated per patient and epileptogenic focus localization was scored for both the ictal and interictal epileptogenic foci. The seizure onset region (focus of ictal epileptiform EEG abnormalities) was based on the description of the original EEG reports, because all EEGs were performed in tertiary referral centers with expertise in pediatric epileptology. All EEG reports were evaluated by one trained researcher, and in case of doubt, a pediatric epileptologist was consulted. A description of both ictal and interictal epileptogenic foci was collected in the electronic Case Report Form.

For each EEG, the authors collected the age at time of recording, the number and type of antiseizure medications, and technical aspects of the EEG recording. Technical aspects of the EEG included the number of electrodes (21-channel, 64-channel, 85-channel/high-resolution, or 128-channel EEG), and its duration in hours. Background pattern was classified in subgroups of “normal background pattern,” “focal slow rhythm,” “diffuse slow rhythm,” or “epileptic encephalopathy/no recognizable physiologic background pattern.” Per EEG, the authors scored whether clinical or electrographical seizures were recorded and whether interictal epileptiform abnormality was detected, with ictal recording defined as recording of at least one clinical or electrographical seizure.

To evaluate regional consistency of ictal EEG findings over time, the authors plotted the reported foci in a simplified brainmap (Fig. 1), and manually categorized regional localizations by side and region, for example, frontal, central, temporal, parietal, or occipital, or a combination of these locations (e.g., “right frontocentrotemporal”). Additional localization options were “bifrontal,” “unclear lateralization or midline,” ‘”hemispheric generalized,” “bilateral generalized,” or “unclear localization (for example, in case of epileptic spasms).” In case of multiple ictal epileptogenic foci per EEG, each focus was individually scored, and if mentioned in the EEG report, one focus could be marked as “dominant.” If two foci in the same region were described as separate localizations in the report of the same EEG, these were classified as two foci. In case of two foci in the same region but described individually on different EEGs, these were classified as likely one focus (i.e., regionally consistent).

**FIG. 1.**
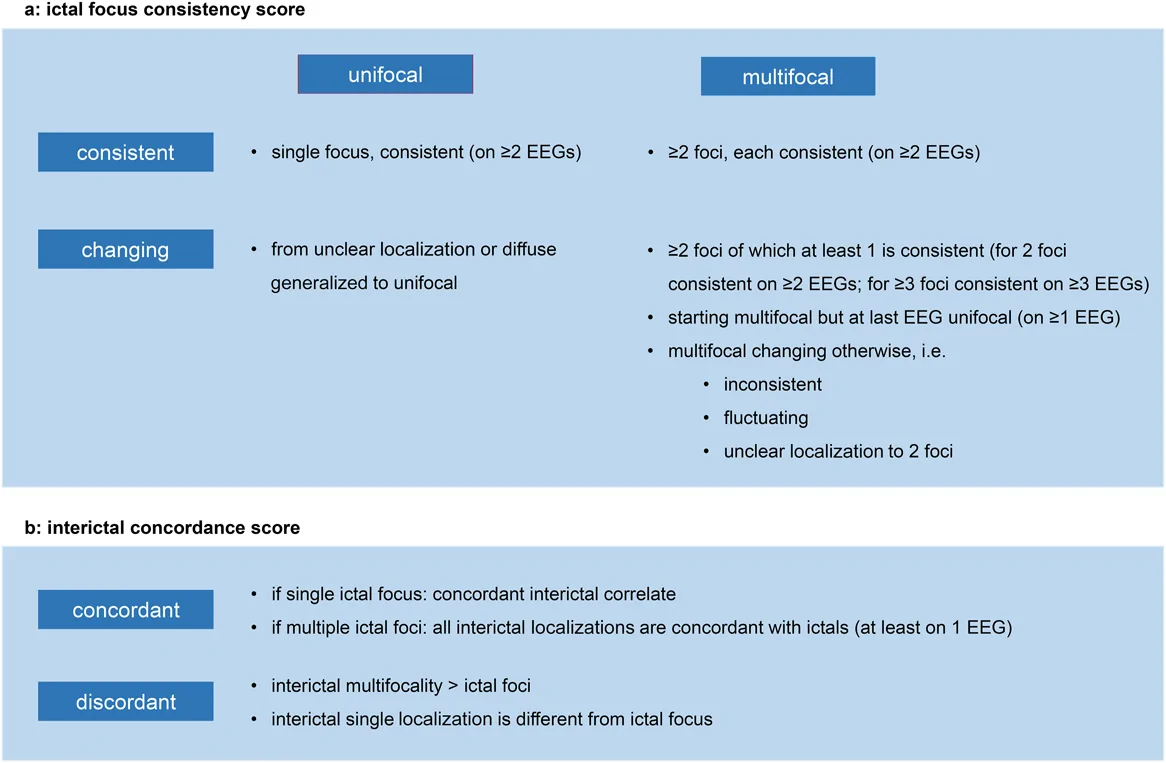
Ictal focus consistency and interictal concordance scores.

For the interictal EEG recording results, the authors evaluated whether epileptiform abnormality was reported or not, and if so whether this was unifocal or multifocal. In case of one or two interictal localizations of epileptiform abnormalities, these were scored according to the same localizations as used for the ictal epileptogenic focus. More than two interictal localizations of epileptiform abnormalities were scored as “diffuse multifocal,” which in case of three or more foci restricted to one hemisphere was scored as “hemispheric diffuse multifocal.”

### Clinical Data Collection

Epilepsy characteristics included age at epilepsy onset, history of epileptic spasms, and seizure semiology. Seizure semiology and seizure type were classified according to the International League Against Epilepsy classification.^13^ The authors additionally scored the number of seizure types localizing to different epileptogenic foci as judged by semiology description. Other clinical parameters such as TSC mutation type, intellectual disability, and autism spectrum disorder diagnosis were collected as well. Treatment variables, including antiseizure medication changes, mammalian target of rapamycin inhibitors, ketogenic diet, and vagal nerve stimulator, were not included as covariates, because these interventions are not expected to change the location of the epileptogenic focus. The authors acknowledge that antiseizure medications could theoretically suppress multifocal activity, making it seem unifocal on EEG, but this remains speculative and was not examined in this study. In addition, the authors evaluated the latest MRI performed during the study period for the radiologically most suspect epileptogenic lesion and included this information in the brainmaps. A highly suspect lesion was defined as having a combination of at least two of the following dysplastic features: T2 hypointense on unmyelinated MRI, cortical thickening, transmantle sign, calcification, or being the largest tuber,^14^—or in case of a larger region being affected such as hemimegalencephaly or large malformations of cortical development. If none of the tubers fulfilled these criteria, no lesion was marked as highly suspect. In the subgroup of patients who underwent epilepsy surgery, the authors evaluated the location and type of resection (or disconnection), and the patient's Engel outcome^15^ 1 year after surgery.

### Study End Points and Statistical Analyses

The primary end point of this study was the consistency of ictal epileptogenic foci on two or more EEGs over time per individual. With a team of experts the authors developed an “ictal focus consistency score” for all different scenarios, as presented in Fig. 1A, and assessed prevalence in each category. For each participant, a visual representation of the epileptogenic foci over time, on simplified brainmaps, was evaluated in a consensus meeting with three experts for scoring of the ictal focus consistency. Epileptogenic foci were considered consistent in case of a single consistent focus, and when two or more foci were identified, which were each consistent on two or more EEGs. When a focus was only seen on a single EEG, consistency could not be demonstrated or ruled out. These foci, together with all other scenarios, were pooled as changing.

A secondary end point was the concordance between ictal epileptogenic foci and localization of interictal epileptiform abnormalities. The authors developed an “ictal and interictal foci concordance score,” presented in Fig. 1B. Concordance scores were determined in the same consensus meeting by the three experts by reviewing the brainmaps of both interictal and ictal epileptogenic foci over time. Prevalence of each concordance score category was assessed. Ictal foci were considered concordant with localization of interictal epileptiform abnormalities when the region(s) of interictal epileptiform abnormalities was the same for all ictal foci. The other scenarios were considered discordant.

The third end point was determinants of consistent ictal epileptogenic foci. The determinants of interest were TSC mutation type (dichotomized to TSC 2 mutation type vs. tuberous sclerosis complex 1 mutation/no mutation identified/mutation not tested), age at onset of epilepsy, history of epileptic spasms, multiple seizures types with seizure semiology judged to localize to different epileptogenic zones, multifocality on interictal EEG during follow-up, and tuber load (dichotomized to intermediate and high tuber load [≥10 tubers, or a (multi)lobar dysplastic lesion] vs. low [<10 tubers]). The analyses of potential determinants were exploratory and hypothesis generating in nature and were not based on prespecified hypotheses. Given the limited sample size and number of outcome events, formal correction for multiple comparisons was not applied. Instead, effect size estimation with 95% confidence intervals was prioritized. Univariable logistic regression was used to study the association between the determinants and consistent epileptogenic foci. To address potential confounding by EEG acquisition characteristics, participant-level variables were created for electrode density and monitoring duration. Electrode density was classified as high density if at least 1 EEG was recorded with 85 electrodes, and as standard-density otherwise (21 electrodes). Monitoring duration was categorized as prolonged if at least one EEG was classified as prolonged, and as routine otherwise. Electrode density showed minimal variation (96.8% standard density) and was, therefore, not included as a covariate. Monitoring duration (81.4% routine vs. 18.6% prolonged) was considered in sensitivity analyses. Given the limited number of events and strong collinearity between several determinants (tuber load, multiple seizure types, and monitoring duration), a full multivariable model was deemed statistically inappropriate. Instead, each determinant was analyzed separately while adjusting for monitoring duration (univariable analyses adjusted for monitoring duration). Stratified analyses by monitoring duration were planned; however, owing to an insufficient number of events in one stratum (routine monitoring), formal stratified regression analyses were not feasible and findings are, therefore, presented descriptively. Odds ratios were converted to risk ratios.^16^ For all analyses, *p* < 0.05 was considered statistically significant. Baseline data were evaluated using descriptive statistics. To assess interrater agreement of ictal focus localization, a second experienced neurophysiologist, masked to the initial assessment, independently evaluated the ictal focus descriptions from the EEG reports in a random 20% sample of patients (9 of 45). Agreement between the two raters was quantified using Cohen κ for complete agreement and the Jaccard index for partial agreement, because some EEGs contained multiple ictal foci. All statistical analyses were performed using R Statistical software version 4.3.2.

## RESULTS

### Baseline Characteristics

The authors included 45 children with TSC in this retrospective, observational study. Median age at epilepsy onset was 4.0 months (interquartile range [IQR] 2.5–8.4) and 30 patients (66.7%) had a history of epileptic spasms. The median number of seizure types, over time, judged to localize to different epileptogenic zones was 3.0 (IQR 1.0–4.0). Tuber load was low in 13 patients (28.9%). Thirteen patients (28.9%) underwent epilepsy surgery. Baseline characteristics of the 45 TSC patients are presented in Table 1.

**TABLE 1.** Baseline Characteristics

Female sex	20 (44.4)
TSC mutation type	
TSC 1	8 (17.8)
TSC 2	31 (68.9)
Not tested/not identified	6 (13.3)
Epilepsy characteristics	
Age at epilepsy onset (months)	4.0 (2.5–8.4)
History of epileptic spasms	30 (66.7)
Seizure types*	3.0 (2.0–5.0)
Seizure types localizing to different epileptogenic zone†	3 (1.0–4.0)
Intellectual disability‡	31 (68.9)
Autism spectrum disorder (diagnosis or suspicion)*	20 (44.4)
MRI data	
Age at last (presurgical) MRI (months)§	123.8 (58.2–186.7)
Tuber load	
Low (<10 tubers)	13 (28.9)
Intermediate (10–20 tubers)	11 (24.4)
High (>20 tubers)	21 (46.7)
Radiologic suspect lesion identified	31 (68.9)
Number of antiseizure medications ever tried	5.0 (4.0–6.0)
Ketogenic diet*	13 (28.9)
mTOR inhibitor treatment*	11 (24.4)
Epilepsy surgery	
Number of patients with epilepsy surgery	13 (28.9)
Age at surgery (months)	53.6 (35.7–65.8)
Type of surgery	
Tuberectomy (plus)	11 (24.4)
(Multiple) lobectomy	0 (0)
(Sub)lobar disconnection	1 (2.2)
Hemispherotomy	1 (2.2)
Other type of brain surgery during study period	
Vagal nerve stimulation	6 (13.3)
Ventriculoperitoneal shunt placement	6 (13.3)

Continuous variables are depicted as means (±SD), or medians (IQR: 25th percentile–75th percentile) if not normally distributed, and count variables as *n* (%).

* During study period.

† Number of seizure types localizing to different epileptogenic zones based on seizure semiology during the study period.

‡ Composed of the developmental quotient, total intelligence quotient, and cognition index, at latest neuropsychological assessment depending on the type of neuropsychological assessment used, and solely for the patients with neuropsychological assessment available.

§ Latest MRI available during the study period.

IQR, interquartile range; mTOR, mammalian target of rapamycin; *n*, number of patients; TSC, tuberous sclerosis complex.

The authors collected data from 379 EEGs for this study. This included 163 ictal recordings and 363 EEGs for the localization of interictal epileptiform abnormalities, and the interictal pattern could not be assessed in 16 EEGs. The median number of ictal EEGs per patient was 3 (IQR 2–4). Median time between the first and last ictal EEG was 28.4 months (IQR 19.0–60.3). Median number of interictal EEGs was 8 (IQR 5–10), with a median of 63.7 months (IQR 35.5–123.7) between the first and last interictal EEG. Background pattern was normal in 176 of 379 EEGs (46.4%). Multifocality on any interictal EEG during the study period was present in 38 patients (84.4%). Baseline characteristics of EEG data are presented in Table 2.

**TABLE 2.** Baseline Characteristics of EEG Data

EEG Characteristics per Patient (*n* = 45)	
Total number of EEGs per patient	8 (6–10)
Number of ictal EEGs per patient	3 (2–4)
Number of interictal EEGs per patient	8 (5–10)
Time between first and last EEG (months)	63.7 (35.5–123.7)
Time between first and last ictal EEG (months)	28.4 (19.0–60.3)
Time between first and last interictal EEG (months)	63.7 (35.5–123.7)
Multifocality on interictal EEG*	38 (84.4)
Electrode density†	
21-channel EEGs only	32 (71.1)
85-channel EEG at 1 point during follow-up	13 (28.9)
Monitoring duration‡	
Routine recordings only	8 (17.8)
Prolonged recording at least once during follow-up	37 (82.2)

EEG Characteristics per EEG (*n* = 379)	
Total number of ictal EEGs	163 (43.0)
Total number of interictal EEGs	363 (95.8)
Number of antiseizure medications at time of EEG	2.0 (1–3)
Type of monitoring per EEG	
21-channel routine recording	297 (78.4)
21-channel prolonged recording	69 (18.2)
85-channel (high resolution)	13 (3.4)
Duration prolonged recording (hours)	24 (23.0–24.0)
Background pattern per EEG	
Normal	176 (46.4)
Focal slow rhythm	44 (11.6)
Diffuse slow rhythm	96 (25.3)
Epileptic encephalopathy/no physiologic background pattern recognizable	63 (16.6)

Continuous variables are depicted as means (±SD), or medians (IQR: 25th percentile–75th percentile) if not normally distributed, and count variables as *n* (%).

* Multifocality on interictal EEG ever during study period.

† Patients were categorized as “high electrode density” if during follow-up at least one EEG was performed with 85-channel electrodes.

‡ Patients were categorized as “prolonged monitoring” if during follow-up at least one EEG had prolonged monitoring.

IQR, interquartile range; *n*, number of patients or EEGs.

### Consistency of Ictal Epileptogenic Foci

In 11 of 45 patients (24.4%, 95% confidence interval [95% CI] 12.9% to 39.5%), ictal epileptogenic foci were considered consistent, while in 34 of 45 patients (75.6%, 95% CI 60.5% to 87.1%), ictal foci changed. In 9 of 45 patients (20.0%, 95% CI 9.6% to 34.6%), there was a single, consistent focus. Multifocal ictal foci were fully consistent in 2 of 45 patients (4.4%, 95% CI 0.5% to 15.2%) and consistent for at least one focus in 14 of 45 patients (31.1%, 95% CI 18.1% to 46.1%). Ictal foci started multifocal but changed to unifocal in 1 of 45 patients (2.2%, 95% CI 0.1% to 11.5%) or they were multifocal changing otherwise in 17 of 45 patients (37.8%, 95% CI 24.2% to 53.0%). In 3 of 45 patients (6.7%, 95% CI 1.4% to 18.3%), ictal epileptogenic foci went from unclear localization or diffuse generalized to unifocal. In 9 of 32 patients (28.1%, 95% CI 13.7% to 46.7%) with ictal multifocality, there was 1 consistent focus initially (on ≥2 or more EEGs) and an additional focus was recorded around the median age of 67.7 months (IQR 32.5–129.2), that is 44.1 months (IQR 18.4–75.5) after the first identified focus. Results of the ictal foci consistency score are presented in Table 3a. Figure 2 shows brainmaps of individual patients in three scenarios; in **Supplemental Digital Content 1** (see **Figure 1**, http://links.lww.com/JCNP/A374), the authors present the brainmaps of all included patients. The radiologic suspected lesion was concordant with (one of) the ictal epileptogenic foci in 3 of 11 patients with consistent epileptogenic foci; in 1 patient the authors did not identify a radiologic highly suspect lesion.

**TABLE 3.** Prevalence of Consistent Ictal Epileptogenic Foci Over time:

(a) Ictal Foci Consistency Score in All Patients	All Patients (*n* = 45)
	Consistency Category
Unifocal	
Single focus, consistent (on ≥2 EEGs)*	9 (20.0%, 95% CI 9.6–34.6%)
From unclear localization or diffuse generalized to unifocal (on ≥1 EEGs)	3 (6.7%, 95% CI 1.4–18.3%)
Multifocal	
Two or more foci, each consistent (on ≥2 EEGs)*	2 (4.4%, 95% CI 0.5–15.2%)
Two or more foci of which at least 1 is consistent (for 2 foci consistent on ≥2 EEGs; for ≥3 foci consistent on ≥3 EEGs)	14 (31.1%, 95% CI 18.1–46.1%)
Starting multifocal but at last EEG unifocal (on ≥1 EEGs)	0 (0.0%, 95% CI 0.0–7.9%)
Multifocal changing otherwise†	17 (37.8%, 95% CI 24.2–53.0%)

(b) Ictal Foci Consistency Score-Subgroup With Epilepsy Surgery	Operated (*n* = 13)	Not Operated (*n* = 32)
	Consistency Category	Consistency Category
Unifocal		
Single focus, consistent (on ≥2 EEGs)*	1 (7.7%, 95% CI 0.2–36.0%)	8 (25.0, 95% CI 11.5–43.4%)
From unclear localization or diffuse generalized to unifocal (on ≥1 EEGs)	2 (15.4%, 95% CI 1.9–45.5%)	1 (3.1, 95% CI 0.1–15.8%)
Multifocal		
Two or more foci, each consistent (on ≥2 EEGs)*	1 (7.7%, 95% CI 0.2–36.0%)	1 (3.1, 95% CI 0.1–15.8%)
Two or more foci of which at least 1 is consistent (for 2 foci consistent on ≥2 EEGs; for ≥3 foci consistent on ≥3 EEGs)	4 (30.8%, 95% CI 9.1–61.4%)	10 (31.2, 95% CI 15.3–50.8%)
Starting multifocal but at last EEG unifocal (on ≥1 EEGs)	1 (7.7%, 95% CI 0.2–36.0%)	0 (0.0%, 95% CI 0.0–10.9%)
Multifocal changing otherwise†	5 (38.5%, 95% CI 13.9–68.4%)	12 (37.5%, 95% CI 21.1–56.3%)

(c) Ictal Foci Consistency Score in Subgroup of Operated Patients–By Postoperative Seizure Outcome	Seizure Freedom (*n* = 7)	Seizure Recurrence (*n* = 6)
	Consistency Category	Consistency Category
Unifocal		
Single focus, consistent (on ≥2 EEGs)*	1 (14.3%, 95% CI 0.4–57.9%)	0 (0.0%, 95% CI 0.0–45.9%)
From unclear localization or diffuse generalized to unifocal (on ≥1 EEGs)	2 (28.6%, 95% CI 3.7–71.0%)	0 (0.0%, 95% CI 11.8–88.2%)
Multifocal		
Two or more foci, each consistent (on ≥2 EEGs)*	1 (14.3%, 95% CI 0.4–57.9%)	0 (0.0%, 95% CI 0.0–45.9%)
Two or more foci of which at least 1 is consistent (for 2 foci consistent on ≥2 EEGs; for ≥3 foci consistent on ≥3 EEGs)	1 (14.3%, 95% CI 0.4–57.9%)	3 (50.0%, 95% CI 11.8–88.2%)
Starting multifocal but at last EEG unifocal (on ≥1 EEGs)	0 (0.0%, 95% CI 0.0–41.0)	0 (0.0%, 95% CI 0.0–45.9%)
Multifocal changing otherwise†	2 (28.6%, 95% CI 3.7–71.0%)	3 (50.0%, 95% CI 11.8–88.2%)

These tables show the prevalence per consistency category of ictal epileptogenic foci over time presented as count variables (%).

* The categories that were considered “consistent” for logistic regression analyses.

† Ictal epileptogenic foci were considered “multifocal changing otherwise” in each of the following: (1) a focus is inconsistent, (2) the focus location is fluctuating, (3) unclear localization to two foci.

CI, confidence interval; *n*, number of patients.

FIG. 2.Three examples (patient A, patient B, patient C) of brainmaps with the evolution of ictal and interictal epileptogenic foci over time.

**Figure:**
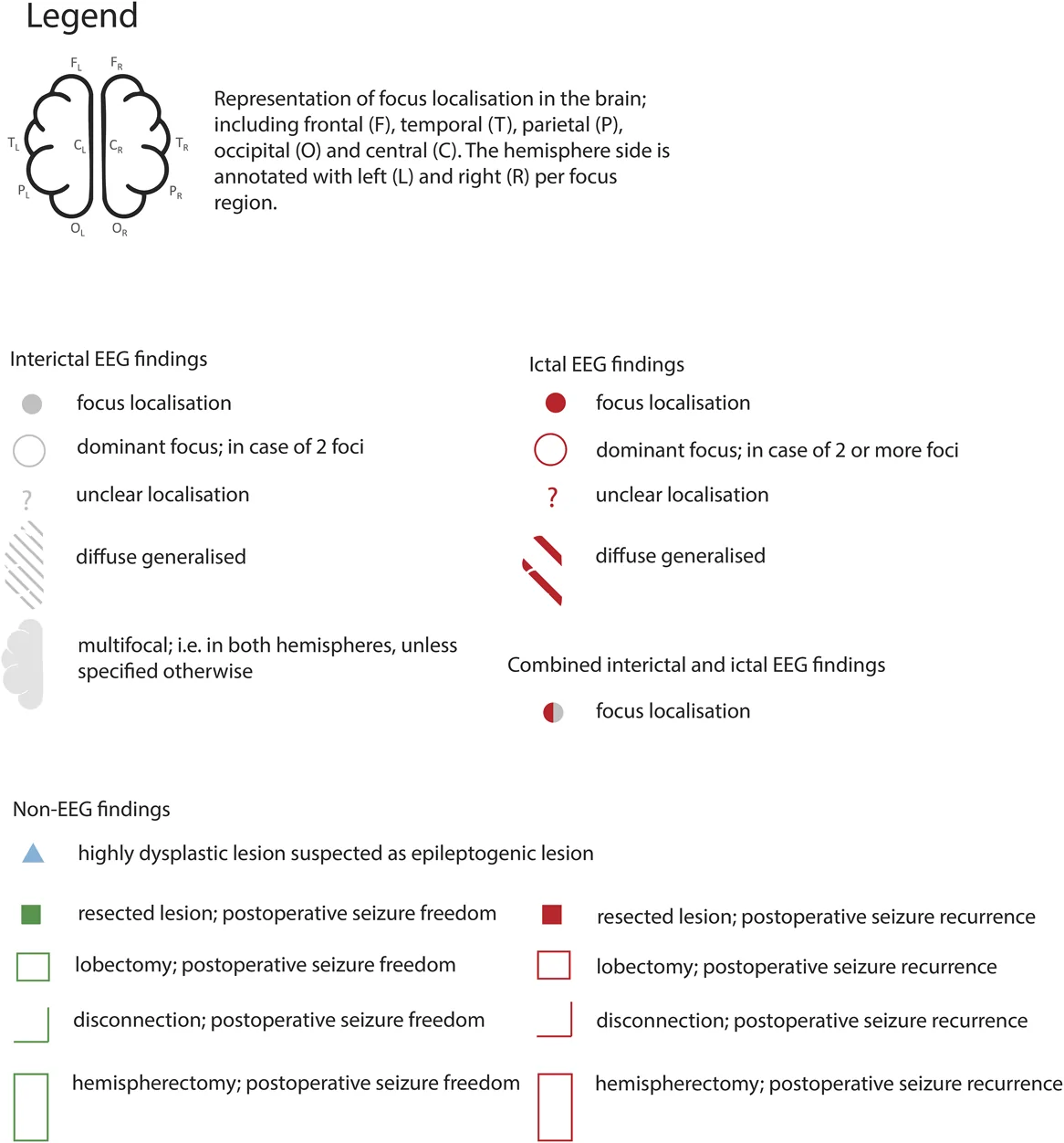

**Figure:**
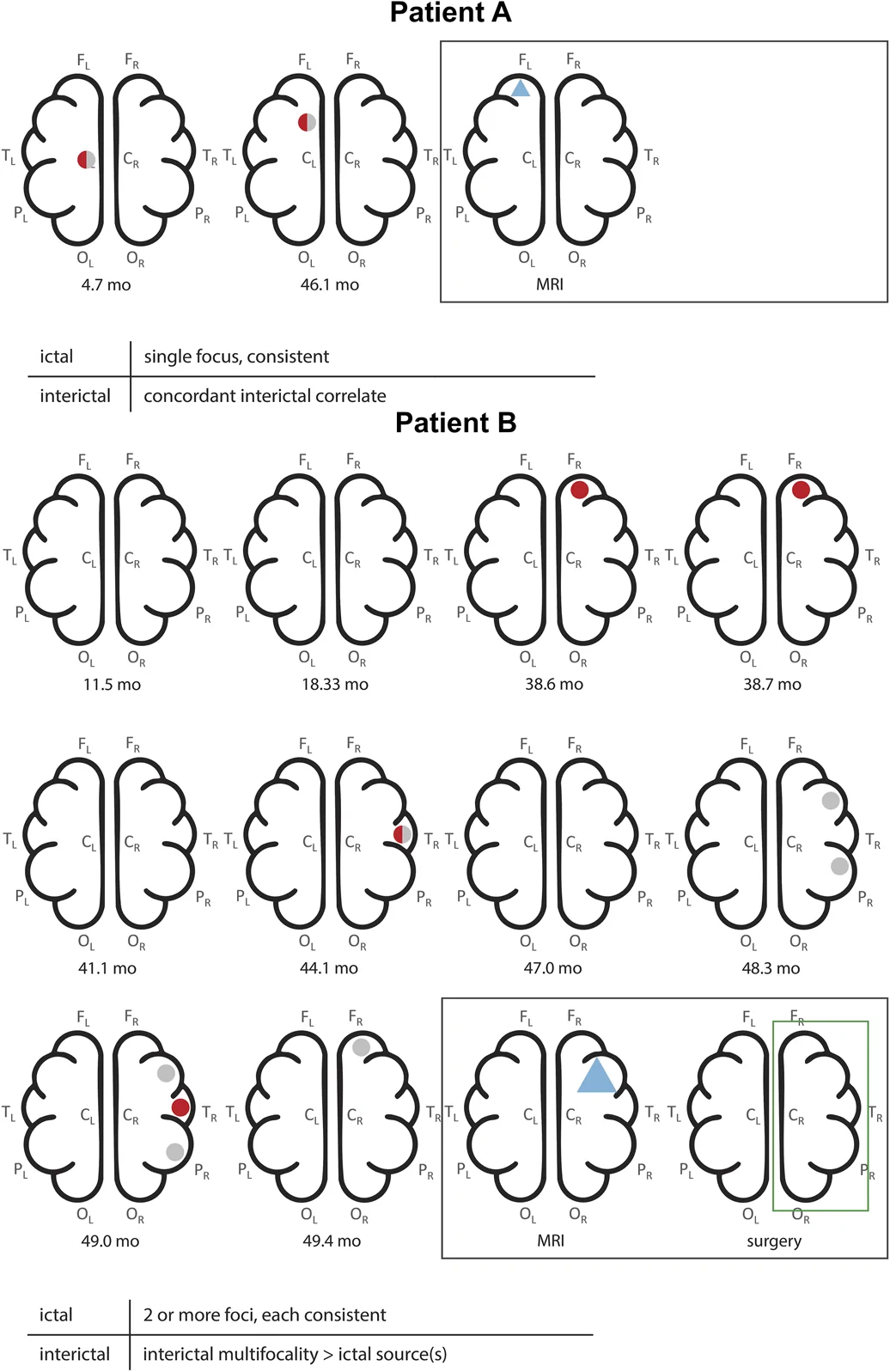

**Figure:**
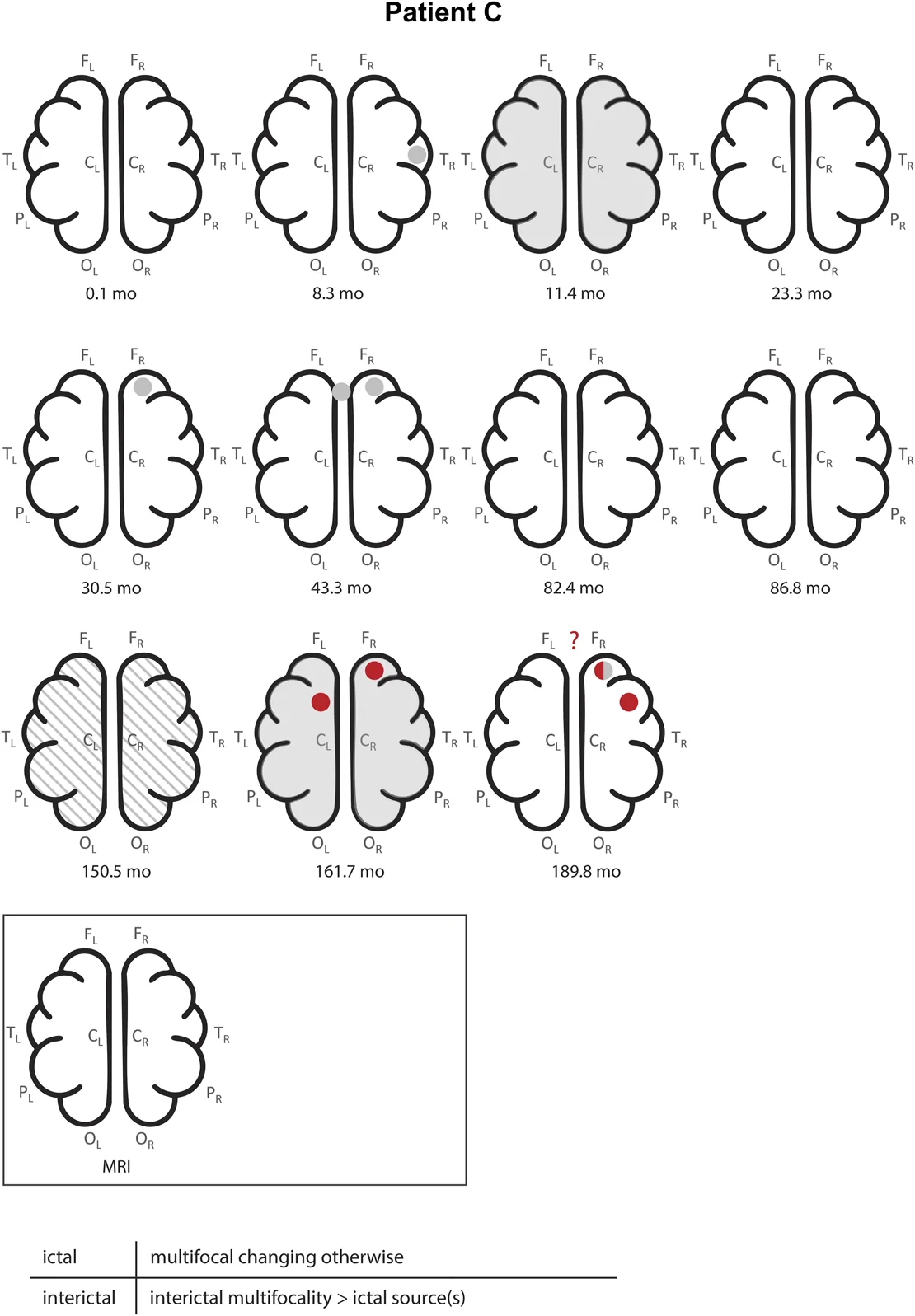

Inter-rater agreement for ictal focus localization, assessed in a random 20% sample of patients (9 of 45), demonstrated substantial agreement between raters (Cohen *κ* = 0.69) and a Jaccard index of 0.81, indicating good concordance in the classification of ictal foci, including cases with multiple foci.

In the subgroup of 13 patients who underwent epilepsy surgery, the authors assessed a single consistent ictal focus in 3 patients (23.1%, 95% CI 5.0% to 53.8%), and a dominant consistent focus in 2 of 13 patients (15.4%, 95% CI 1.9% to 45.5%); these patients reached postoperative seizure freedom (Table 3b). All six nonseizure-free children had ictal multifocality while surgery involved a single region. Prevalence for each ictal foci consistency score in the epilepsy surgery group is presented in Table 3c.

### Concordance Between Ictal and Interictal Epileptogenic Foci

Of nine patients with a single ictal focus, only one (2.2%, 95% CI 0.1% to 11.5%) showed a concordant interictal localization. Only 2 of 45 patients (4.4%, 95% CI 0.5% to 15.2%) with multiple ictal foci showed concordant interictal findings. In 42 of 45 (93.3%, 95% CI 81.7% to 98.6%) patients, interictal epileptiform abnormalities localized to more regions than ictal foci, and were, therefore, discordant. Interictal concordance scores are presented in Table 4.

**TABLE 4.** Concordance Between Interictal and Ictal Epileptogenic Foci Over Time

	All Patients (*n* = 45)
	Concordance Category
Concordant	
If single ictal focus: concordant interictal correlate	1 (2.2%, 95% CI 0.1–11.5%)
If multiple ictal foci: all interictal localizations of epileptiform abnormalities are concordant with ictal foci (at least on one EEG)	2 (4.4%, 95% CI 0.5–15.2%)
Discordant	
Interictal multifocality > ictal foci	42 (93.3%, 95% CI 81.7–98.6%)
Interictal single focus is different from ictal focus	0 (0.0%, 95% CI 0.0–7.9%)

This table shows the prevalence per ictal and interictal concordance score category presented as count variables (%).

CI, confidence interval; *n*, number of patients.

### Determinants of Ictal Consistency

Patients with multifocality on interictal EEG during the study period had a 68% lower likelihood of having consistent ictal foci (risk ratios 0.32; 95% CI 0.06–0.97, *p* = 0.04). The authors did not identify other determinants of ictal consistency. Results of the univariable regression analysis are presented in Table 5. Univariable analyses adjusted for monitoring duration yielded similar point estimates, although the previously significant association for multifocality on interictal EEG did not persist (adjusted risk ratio 0.51; 95% CI 0.11–1.08, *p* = 0.10) (Table 5). This attenuation suggests that the observed effect is unstable, particularly in the context of sampling variability and limited power. Stratified analyses by monitoring duration were limited by the small number of events in one stratum (routine monitoring); therefore, formal regression analyses were not feasible. Descriptive comparison showed that the prevalence of consistent ictal foci was 50.0% in participants with routine monitoring and 18.9% in those with prolonged monitoring. Given the small number of events, no formal statistical inference can be drawn from these stratified comparisons. Full descriptive stratified results are provided in **Supplemental Digital Content 1** (see **Table S1**, http://links.lww.com/JCNP/A374).

**TABLE 5.** Association Between Determinants and Consistent Ictal Epileptogenic Foci

**(a) **Univariable Analyses of Determinants and Consistent Ictal Epileptogenic Foci	RR (95% CI)	*P*
TSC 2 mutation*	0.54 (0.15–1.41)	0.24
Age at onset of epilepsy (months)	1.00 (0.97–1.02)	0.91
History of epileptic spasms	0.87 (0.26–2.16)	0.81
Multiple seizure types†	0.60 (0.17–1.53)	0.33
Multifocality on interictal EEG‡	0.32 (0.06–0.97)	0.04
Intermediate or high tuber load§	1.08 (0.31–2.76)	0.89

(b) Adjusted Univariable Analyses for Monitoring Duration	aRR (95% CI)	*P*
TSC 2 mutation*	0.64 (0.20–1.14)	0.18
Age at onset of epilepsy (months)	1.00 (0.96–1.02)	0.80
History of epileptic spasms	1.13 (0.42–1.83)	0.76
Multiple seizure types†	0.77 (0.27–1.38)	0.48
Multifocality on interictal EEG‡	0.51 (0.11–1.08)	0.10
Intermediate or high tuber load§	1.03 (0.37–1.73)	0.95

(a) The results of the univariable regression analyses of determinants and consistent ictal epileptogenic foci. (b) The results of the univariable regression analyses adjusted for monitoring duration. *P*-value <0.05 was considered statistically significant.

* TSC mutation type was dichotomized to TSC 2 mutation type, and TSC 1 mutation type/mutation type not identified/not tested.

† Dichotomous variable defined as multiple seizure types with seizure semiology localizing to different epileptogenic zones.

‡ Multifocality on interictal EEG ever during study period.

§ Intermediate and high tuber loads were defined as, respectively, 10 to 20 and >20 tubers.

95% CI, 95% confidence interval; aRR, adjusted risk ratio; TSC, tuberous sclerosis complex.

## DISCUSSION

In this study, the authors evaluated the longitudinal consistency of ictal epileptogenic foci in patients with TSC using repeated ictal EEG recordings. The authors found that ictal foci were consistent over time in approximately one quarter of TSC patients who have not (yet) undergone epilepsy surgery, whereas the majority showed changing localization patterns. Interictal epileptiform abnormalities were discordant with ictal foci in most patients. Exploratory analyses suggested a nominal association between interictal multifocality and ictal inconsistency, but this finding was not robust after adjustment and should be interpreted with caution. All six patients who underwent epilepsy surgery but had postoperative seizure recurrence showed preoperative ictal multifocality.

The evolution of epileptogenic foci over time in TSC has not been extensively studied. One study evaluated clinical epilepsy and EEG characteristics in 38 patients with TSC. Of the 15 patients with focal seizures and ictal EEGs available, 9 had seizures originating from a consistent single focus over time, 4 had seizures arising from 2 or 3 foci independently, and 2 had shifting foci during disease course.^10,17^ Although Ohtsuka et al. described predominantly consistent ictal seizure onset patterns in a small subset of patients, methodological differences—including limited ictal EEG sampling, absence of a formal longitudinal consistency framework, and differences in patient age and EEG technology—likely explain the more dynamic ictal focus patterns observed in this study. Another study reported on consistency of interictal epileptiform activity in 21 patients with 3 or more EEGs available, with a median interval between the first and last EEG of 9 years (range 3–45 years) by reviewing the original EEGs.^11^ In 8 patients, interictal epileptic activity was consistent in one or two localizations, and in 13 patients interictal activity was detected in ≥3 regions, with 1 dominant consistent region of interictal epileptiform activity in 90% of patients. Jansen et al. reported a high degree of consistency of interictal epileptiform activity during long-term follow-up; however, their study focused exclusively on interictal EEG findings and emphasized the presence of a dominant focus, even in the context of multifocal activity. Electroencephalogram recordings were obtained at relatively sparse, clinically driven time points, favoring identification of long-term stable patterns rather than short-term dynamics. In contrast, this study evaluated ictal epileptogenic foci using repeated ictal EEGs with higher temporal resolution, which increases sensitivity to evolving or alternating seizure onset patterns. Finally, concordance between the dominant interictal localization and the ictal foci was studied by reviewing the EEGs of 19 TSC patients, revealing concordance in 11 of 14 patients (79%).^18^ The markedly lower interictal–ictal concordance observed in this study is likely explained by differences in study design, including longitudinal versus cross-sectional assessment, a stricter definition of concordance, and differences in patient selection.

The analyses of potential determinants of ictal focus consistency were exploratory and intended to generate hypotheses rather than establish definitive associations. Although interictal multifocality showed a nominal inverse association with ictal consistency in unadjusted analyses, this association was attenuated and no longer statistically significant after adjustment for monitoring duration. In the context of multiple testing, limited sample size, and a small number of outcome events, this finding should be interpreted with caution and may reflect chance rather than a true underlying relationship. Interictal multifocality should, therefore, not be considered a reliable or independent predictor of ictal focus consistency based on the present data.

In the surgical subgroup, patients with unifocal or dominant ictal epileptogenic foci more often achieved postoperative seizure freedom than those with multifocal ictal patterns. These findings are based on a small and selected cohort and do not allow for formal statistical inference. The analyses were, therefore, intended to be descriptive and hypothesis generating, illustrating how longitudinal ictal EEG patterns may relate to surgical outcome rather than establishing a definitive predictive relationship. Potential confounding factors, such as imaging characteristics, extent of resection, and other established prognostic variables, could not be accounted for. Confirmation of these observations requires larger cohorts with sufficient power and multivariable analyses.

Previous studies have proposed the concept of a network epileptogenic zone organization, because epileptogenicity in some patients might result from a connection of more than one epileptogenic lesions or foci.^19^ There is evidence favoring the network paradigm in TSC, showing the involvement of more than one tuber or dysplastic lesion in seizure origin, although it remains unclear which patient characteristics or tuber properties are associated with this pathophysiology.^20–23^ The concept of epileptogenic networks could help in the understanding of epileptogenic foci dynamics. In the patients with changing ictal epileptogenic foci, without change in semiology, the authors could hypothesize that these foci are all part of the same epileptogenic network, but over time the “most active” epileptogenic core changes. For those with single or multiple consistent ictal foci, the epileptogenic zone may be organized as simple focal lesional site(s) and not as part of an organized network.^19^ If an epileptogenic network drives the shifting of epileptogenic foci over time in patients with TSC, this might increase susceptibility to the activation of a new epileptogenic center after the surgical resection if the network is not resected completely. The influence of brain maturation on the consistency of ictal epileptogenic foci should, however, be confirmed in a larger cohort of patients with TSC, as well as its relation to surgical outcome. In addition to the network maturation hypothesis, alternative explanations for apparent changes in ictal focus over time should be considered. Such variability may partly reflect sampling error related to the limited spatial resolution and coverage of scalp EEG, particularly for deeper or mesial regions or when electrode placement differs across recordings. Furthermore, changes in antiseizure medication may transiently modify cortical excitability or seizure propagation patterns, thereby altering the electrographic manifestation of seizures. These medication-related effects are unlikely to produce a genuine shift in the underlying epileptogenic focus but may influence how seizure activity is represented or detected on scalp EEG.

A concern that arises from this study is the diagnostic accuracy of the scalp EEG in localizing the epileptogenic zone. Accuracy in localizing the epileptogenic zone with ictal EEG was previously compared with high-resolution electric source imaging, revealing that the ictal EEG was localizing in 54% of patients in contrast to 67% for high-resolution electrical source imaging. The ictal EEG showed concordance with the resected area in only 33% of postoperative seizure-free cases, compared with 67% for high-resolution electric source imaging.^24^ Another study revealed that epileptogenic sources identified on magnetoencephalography are even closer to the presumed epileptogenic tuber than similar sources on high resolution EEG.^25^ That scalp EEG has its limitations in detecting the epileptogenic zone was already established in previous studies including patients with epilepsy of various etiology,^26–28^ and there is general consensus that the identification of the epileptogenic zone in presurgical evaluation benefits from the use of multimodal imaging,^29–31^ or intracranial monitoring.^20,32^ Despite its limitations, ictal scalp EEG monitoring remains the first presurgical evaluation technique to identify a possible epileptogenic region. Source imaging—with high-resolution electric source imaging or magnetoencephalography—can have additional value, and multimodality fusion of presurgical findings is often required to generate a reliable hypothesis on the epileptogenic zone. Thereafter, in patients with TSC, pre- or perioperative invasive monitoring is often needed to further delineate the epileptic zone and tailor the resection, to control the seizures of target.^33^

This study examines consistency of ictal epileptogenic foci over time in patients with TSC using a scoring system that is applicable in clinical practice. A limitation of this study is that foci were interpreted by EEG reports without reviewing the EEG recordings themselves. Intra- and interrater observer variability for scalp EEG was previously reported as moderate.^34^ However, all presurgical investigations in this center are discussed in a multidisciplinary team meeting, including a team of three experienced pediatric neurophysiologists/epileptologists. To further assess reliability, the authors conducted an interrater agreement analysis on a random 20% sample of patients, which demonstrated substantial agreement between two independent neurophysiologists (Cohen *κ* = 0.69). This level of agreement supports the reproducibility of ictal focus classification based on EEG reports, which are routinely used in clinical practice to inform hypotheses regarding seizure onset localization. At the same time, it does not imply excellent diagnostic accuracy or warrant interpretation in isolation. The observed variability likely reflects intrinsic limitations of scalp EEG, including limited spatial resolution and sampling effects, as well as reliance on retrospective report interpretation. Accordingly, the interrater analysis should be interpreted as supporting the reliability of scalp EEG-based localization within a multimodel presurgical evaluation framework, rather than as validation of scalp EEG as a standalone localizing tool. Second, duration of recording varied from “standard EEG recording (30–60 minutes)” to “prolonged recording” (median duration 24 hours, IQR 23.0–24.0). Longer monitoring will likely result in capturing of more seizures and increase the odds of finding multiple ictal foci. This cohort consisted for 78.4% of “standard EEGs,” indicating that the results could present an underestimation of multifocality. In addition, for the ictal EEGs, the authors did not differentiate between clinical and electrographical seizures that may be relevant in presurgical work-up. In young or disabled children, it is sometimes difficult to distinguish the two, because clinical symptoms may be subtle and subjective leading to underreporting of clinical symptoms during a seizure, and, therefore, a seizure might be incorrectly classified as electrographical. Moreover, the authors included patients with at least two ictal EEGs available. Electroencephalograms are often performed in response to change in clinical presentation, to explore the possibility of shift or evolution in epileptogenic foci, implicating that this cohort is selected. This selection criterion may preferentially include patients with evolving or refractory epilepsy, thereby limiting generalizability. However, these features are common in TSC, and the cohort, therefore, represents a clinically relevant part of the broader TSC population. The reported proportion of patients with consistent ictal epileptogenic foci should accordingly be interpreted as descriptive of this selected cohort rather than as a population-level prevalence estimate. Finally, the relatively small sample size and the limited number of patients with consistent ictal epileptogenic foci restricted the statistical power of the analyses, particularly for the assessment of potential determinants and surgical outcomes. This is reflected by wide confidence intervals and imprecise effect estimates. As a result, both false negative and false positive findings are possible, and the absence of statistically significant associations should not be interpreted as evidence of no effect. The analyses of potential determinants were, therefore, exploratory and hypothesis generating. Larger, prospectively designed studies with sufficient power, prespecified hypotheses, and appropriate adjustment for multiple testing are required to confirm these findings and to further clarify the clinical relevance of ictal focus consistency.

Consistency of the epileptogenic zone has important implications for presurgical evaluation. Patients with a unifocal regionally consistent ictal focus seem the most attractive surgery candidates beforehand. However, in cases of ictal multifocality with one dominant consistent focus, epilepsy surgery for targeted seizures needs to be considered, because improved quality of life or comorbidities are seen in patients free of targeted seizures. For the other consistency subcategories, epilepsy surgery might be optional as well, dependent on multiple factors such as patient's age, comorbidities, and results of other presurgical investigations. The authors could assume that with brain maturation, seizures become more localized and, therefore, epileptogenic foci more refined and consistent, because brain structures and connectivity patterns stabilize during development.^35^ The influence of brain maturation on consistency of ictal epileptogenic foci should, however, be confirmed in a larger cohort of patients with TSC, as well as the relationship between consistency and surgical outcome.

## CONCLUSIONS

In this relatively young cohort of patients with TSC, approximately 25% were found to have consistent ictal epileptogenic foci over time. The observed instability in ictal localization may reflect underlying biologic processes, such as maturation or reorganization of epileptogenic networks, while also being influenced by factors related to EEG sampling and study design. In light of presurgical evaluation and understanding of risk of postoperative seizure recurrence, future research should further explore how ictal consistency affects surgical outcomes.

## Supplementary Material
