# Supplementary material for: Consistency of Ictal Epileptogenic Foci in Children With Tuberous Sclerosis Complex and Its Clinical Predictors
Source: J Clin Neurophysiol. 2026 May 20;43(7):584–96. doi: 10.1097/WNP.0000000000001260 (PMC13716295; doi:10.1097/WNP.0000000000001260)
Supplement: Supplementary file 1 [file jcnp-43-584-s001.pdf]

Supplementary Figure S1. Individual brainmaps with the evolution of ictal and interictal epileptogenic foci over time

This figure shows the brainmaps with the evolution of ictal and interictal epileptogenic foci over time for all study participants. Below we presented the legend for interpretation of the figures.

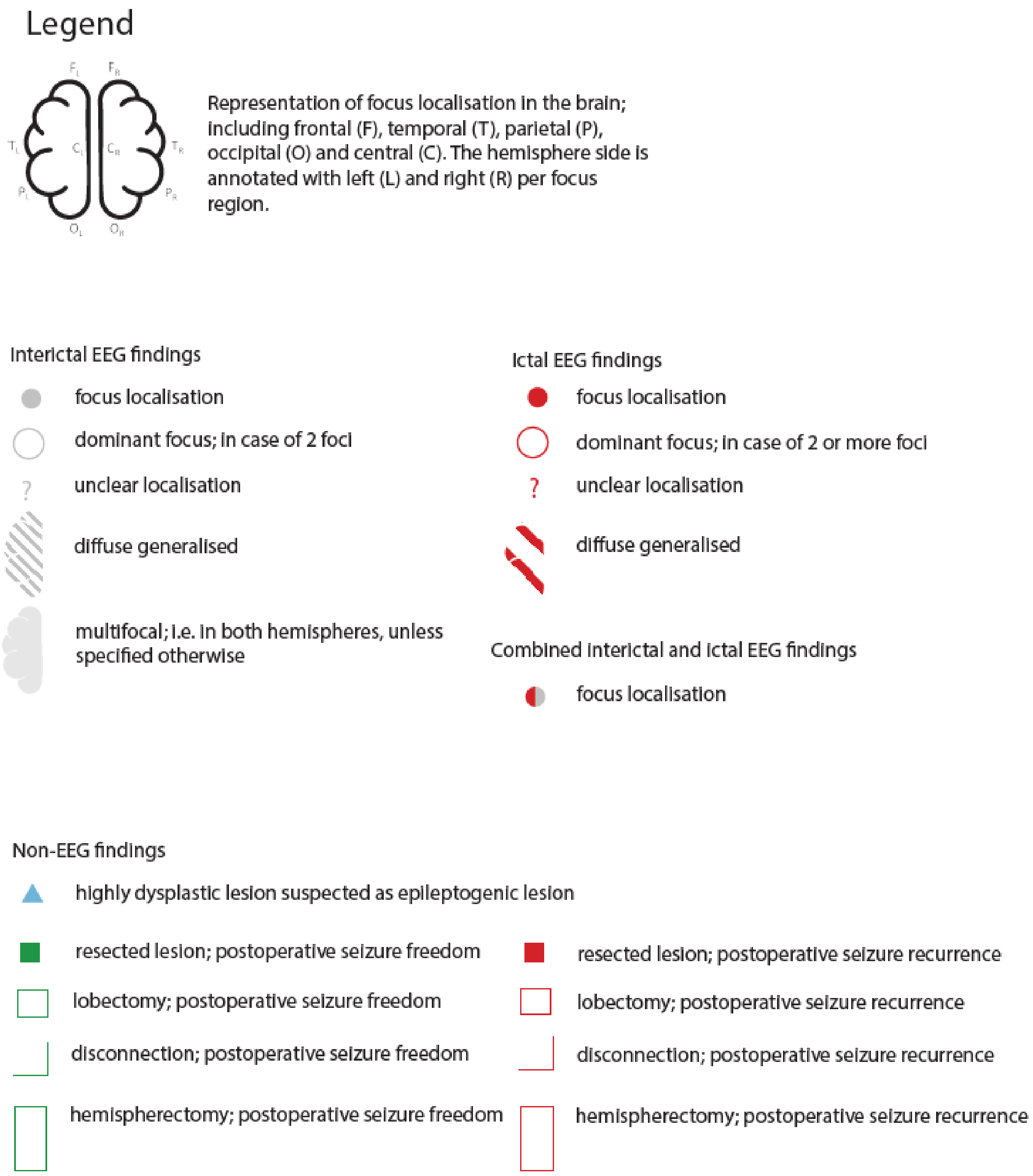

patient 1

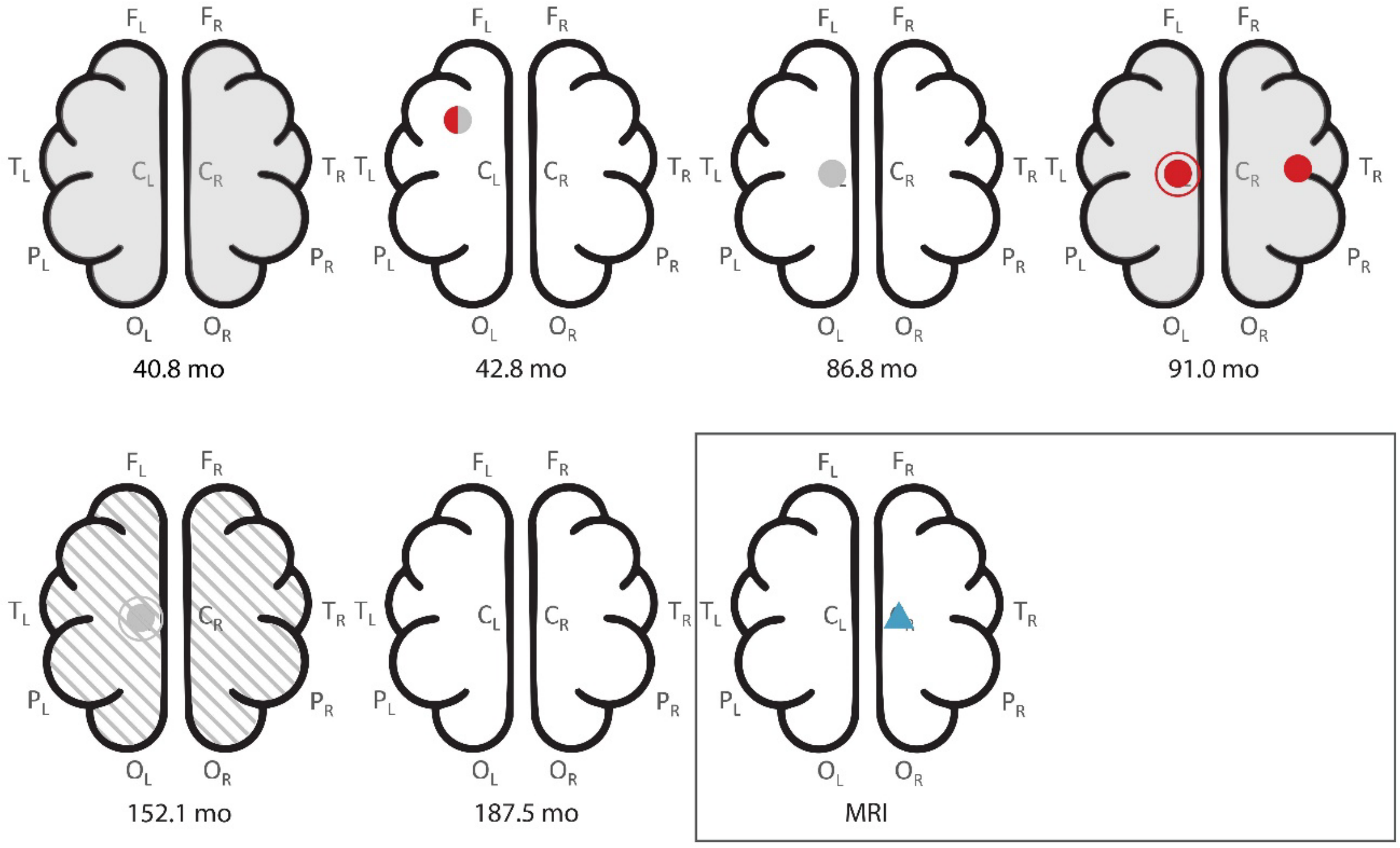

ictal	2 or more foci of which at least 1 is consistent
interictal	interictal multifocality > ictal source(s)

patient 2

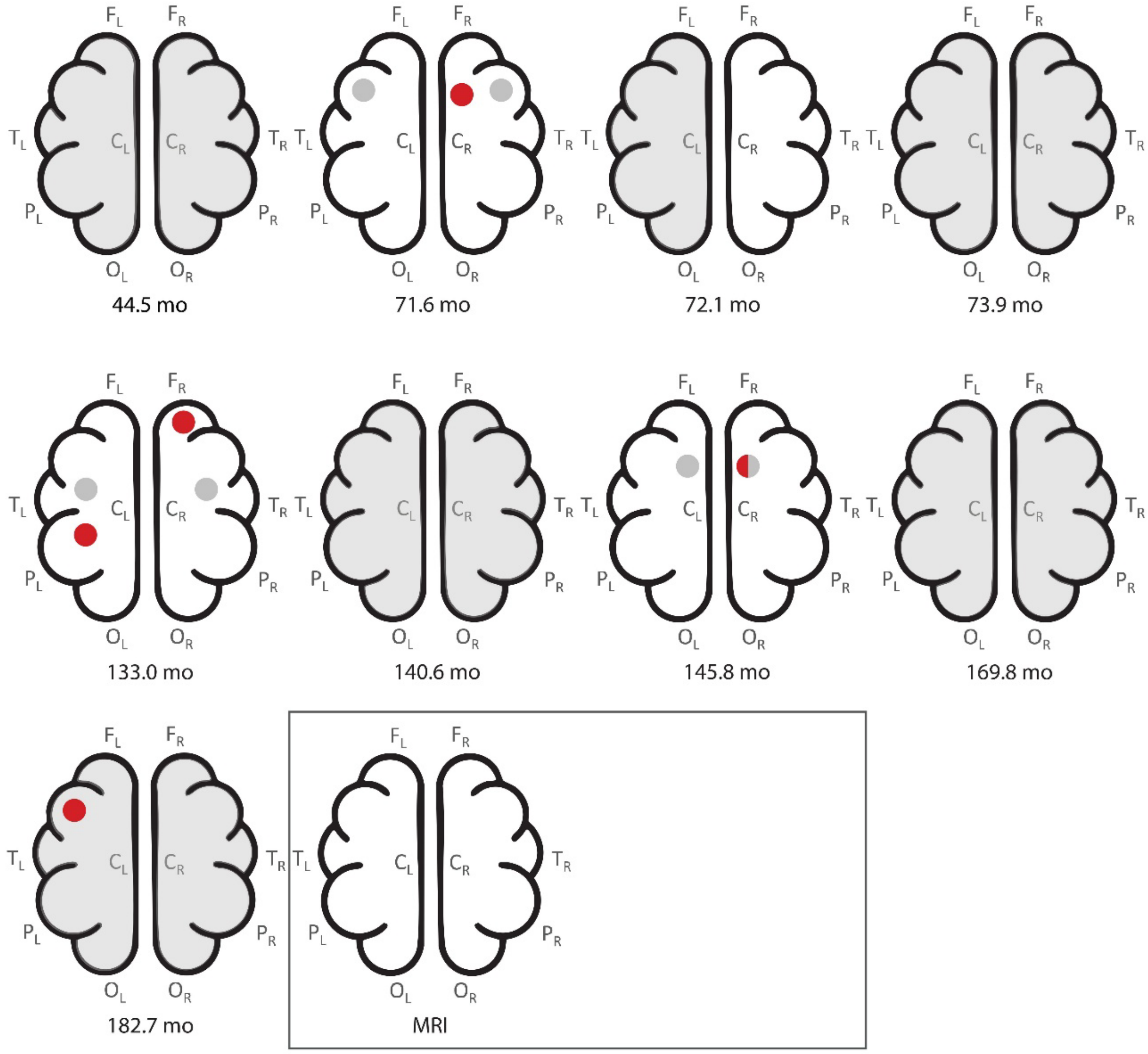

ictal	multifocal changing otherwise
interictal	interictal multifocality > ictal source(s)

patient 3

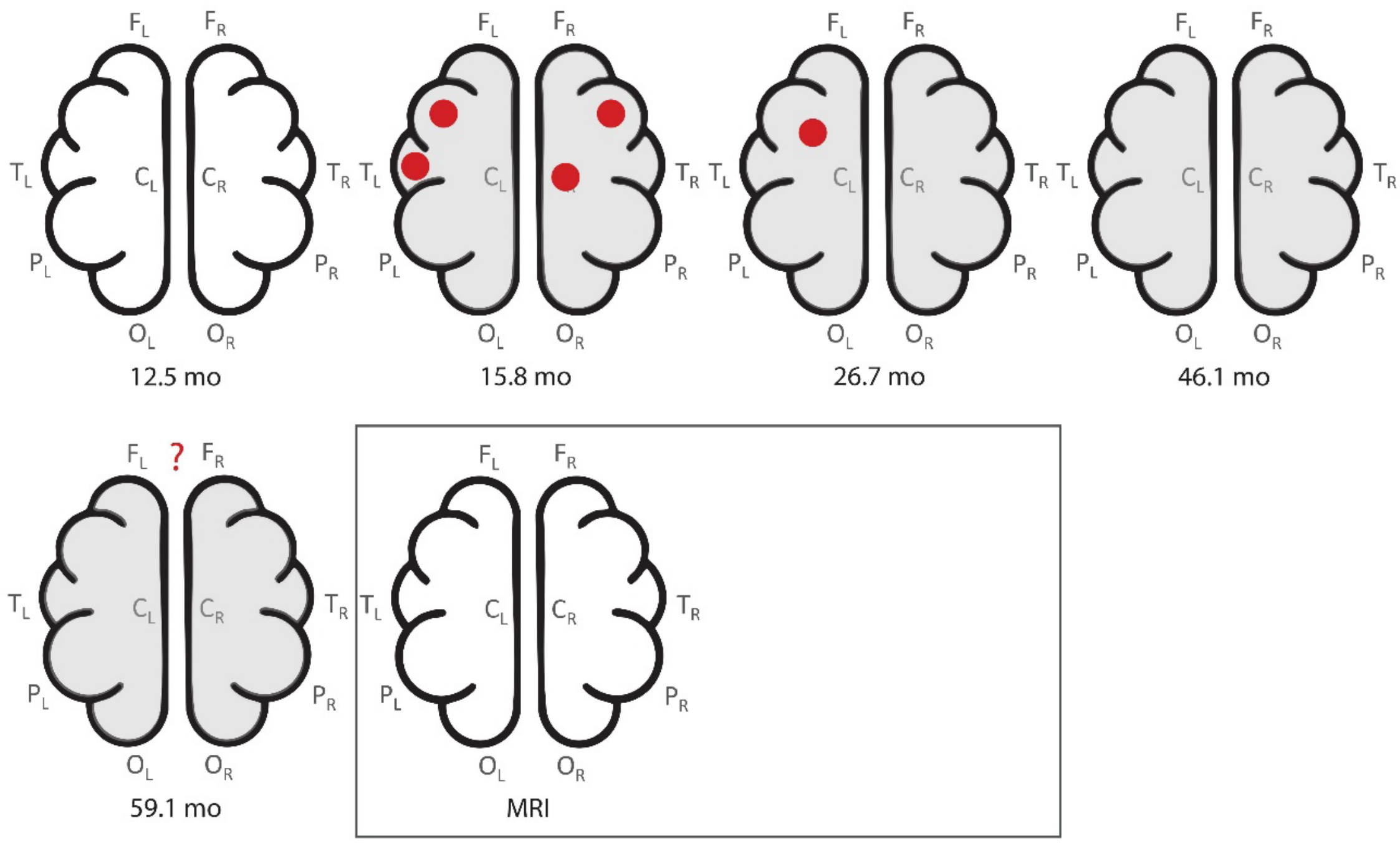

ictal	multifocal changing otherwise
interictal	interictal multifocality > ictal source(s)

patient 4

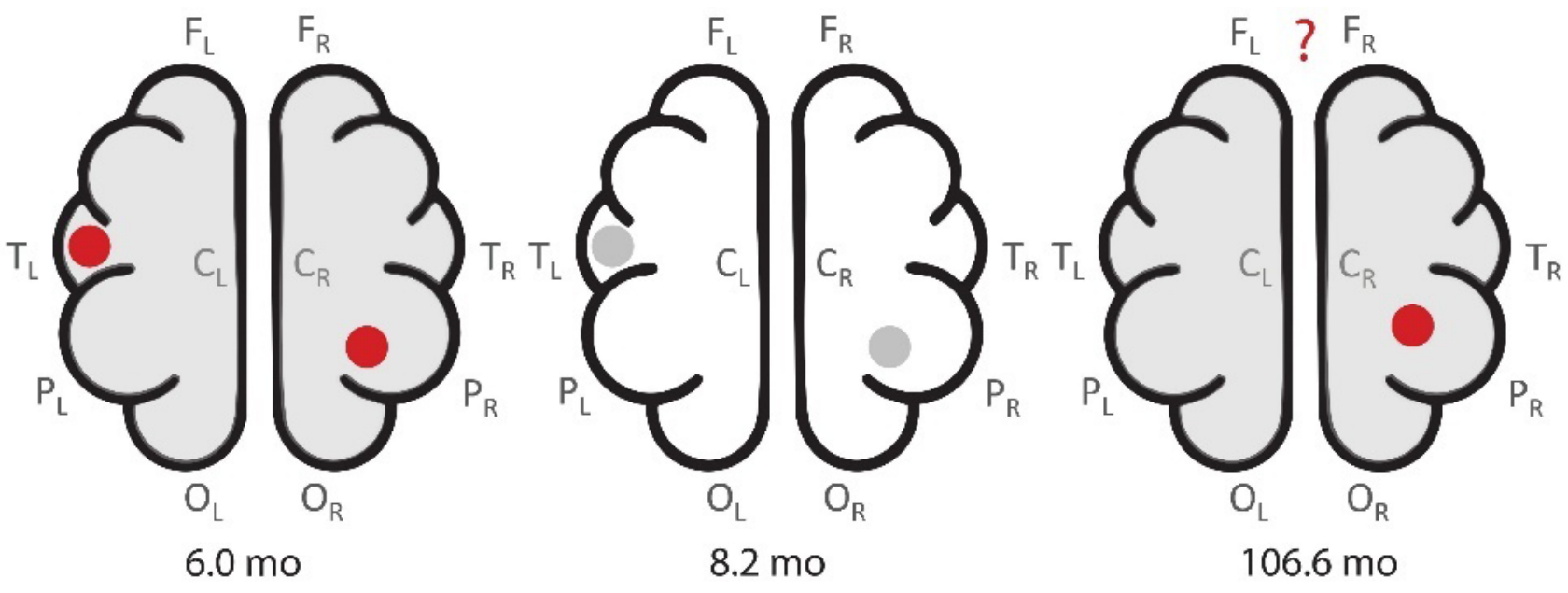

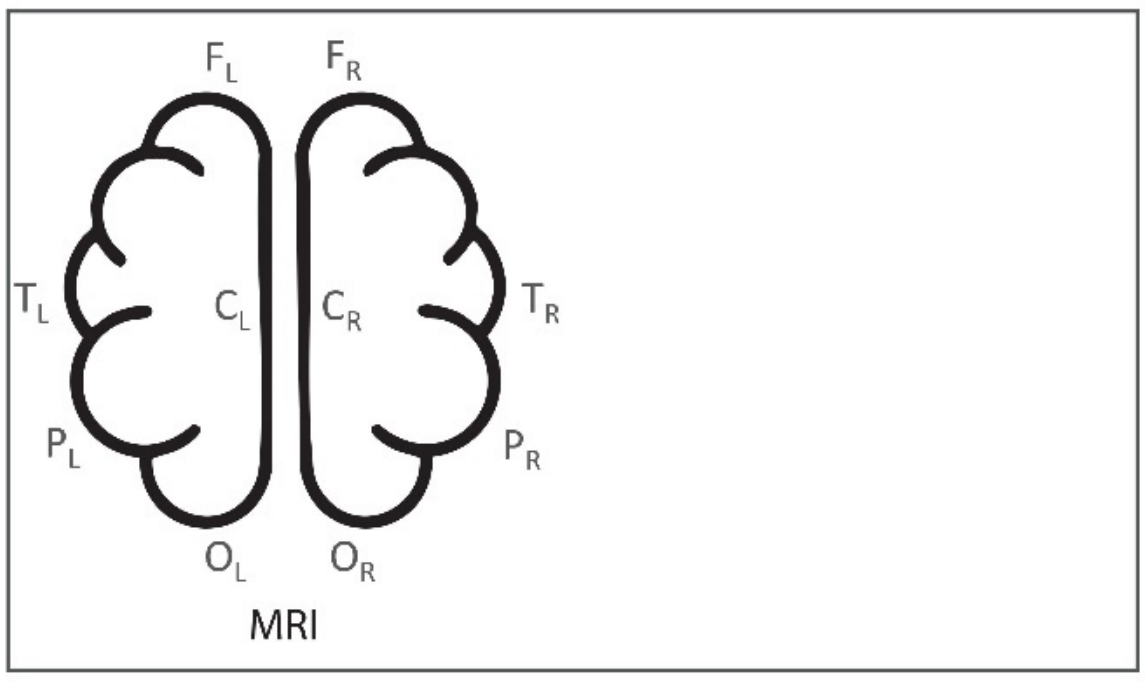

ictal	2 or more foci of which at least 1 is consistent
interictal	interictal multifocality > ictal source(s)

patient 5

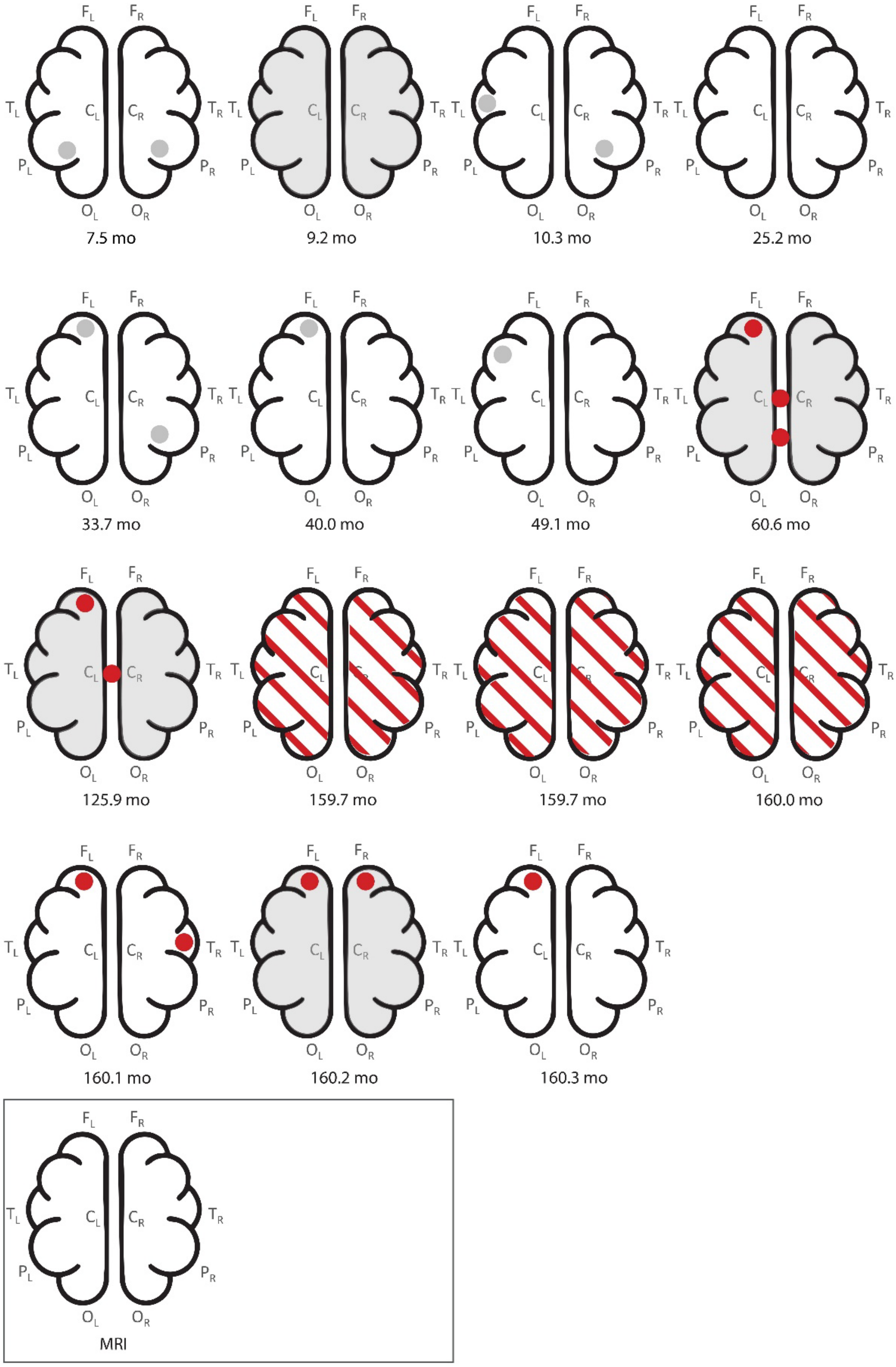

ictal	multifocal changing otherwise
interictal	interictal multifocality > ictal source(s)

patient 6

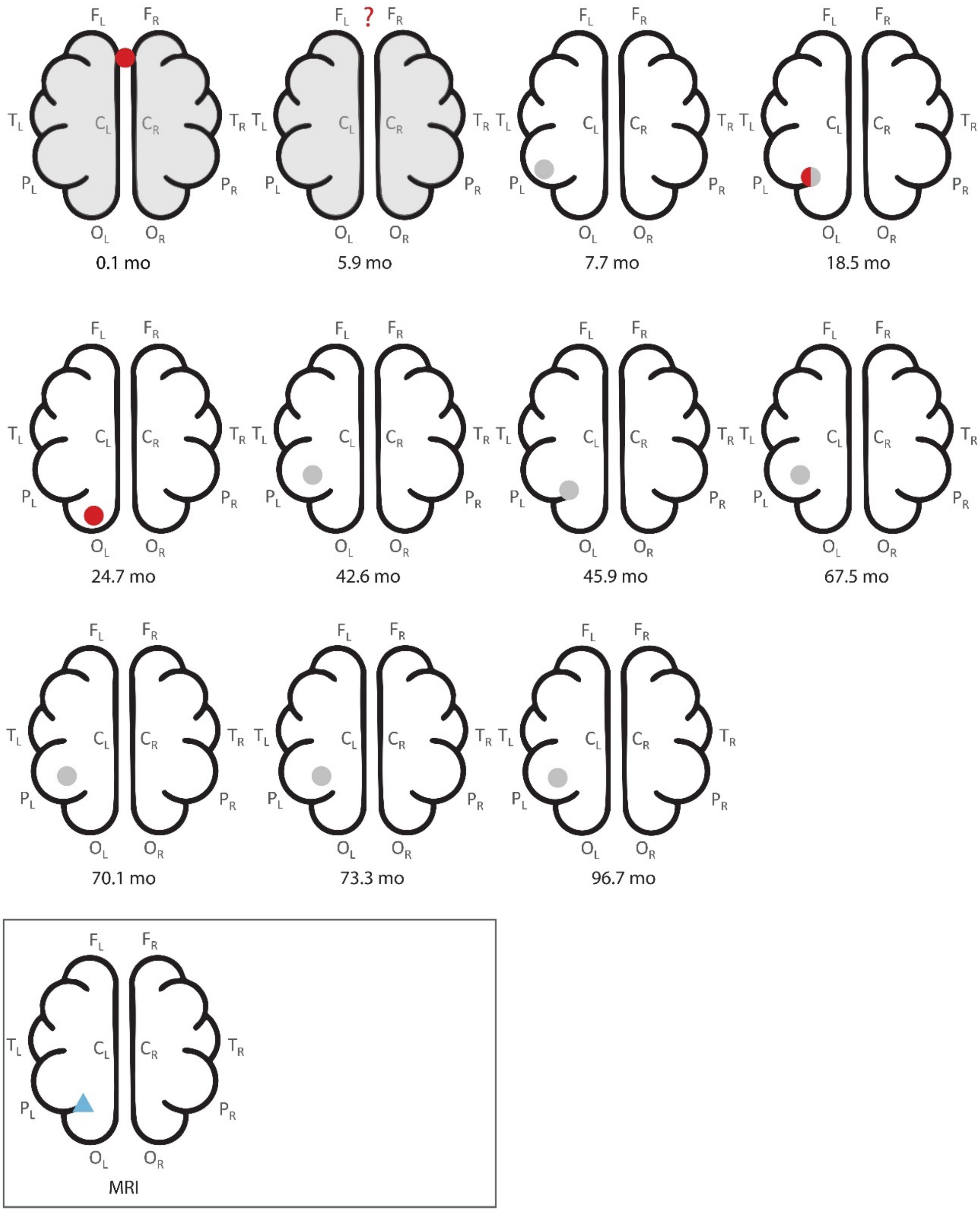

ictal	2 or more foci of at least 1 is consistent
interictal	interictal multifocality > ictal source(s)

patient 7

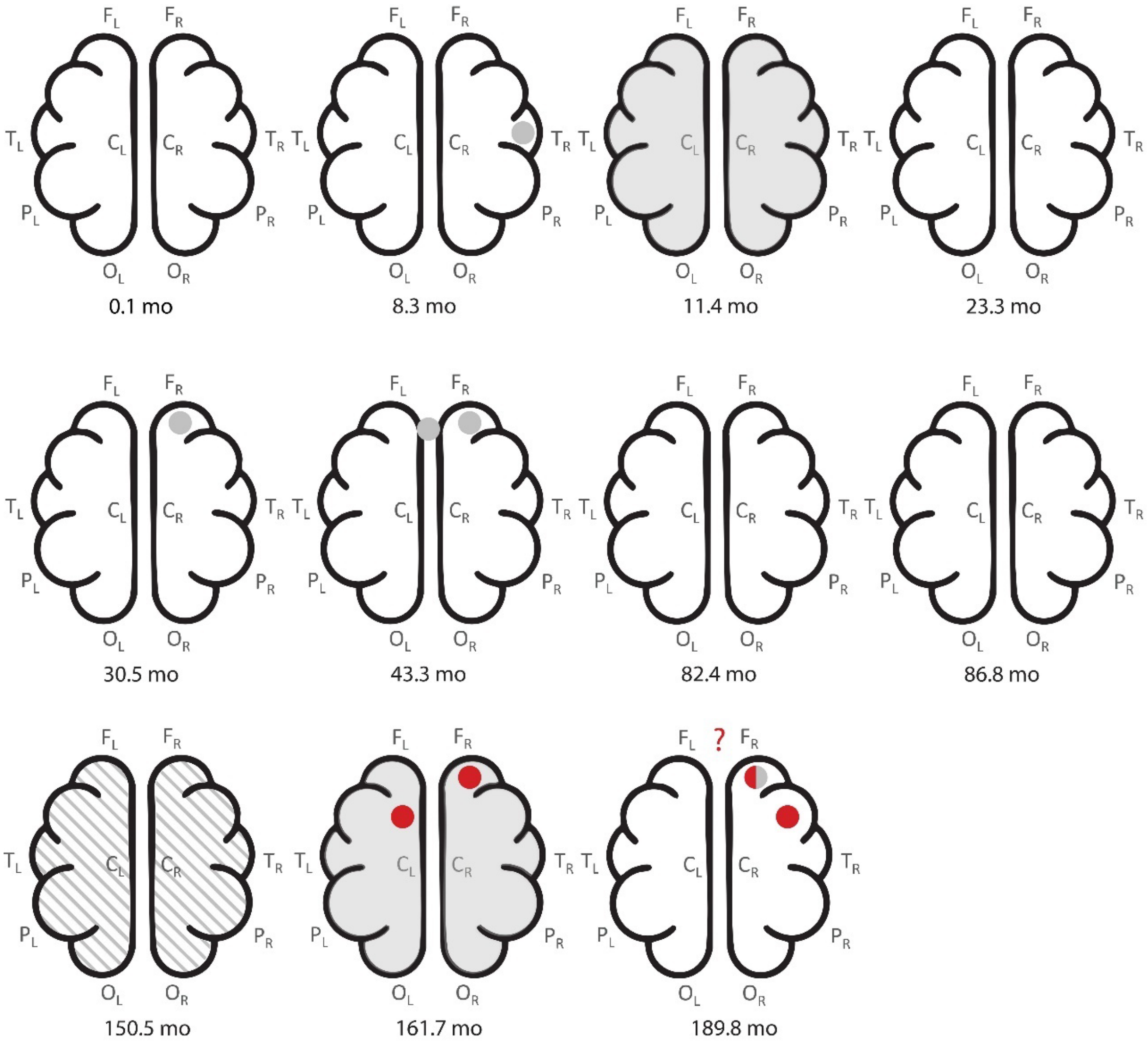

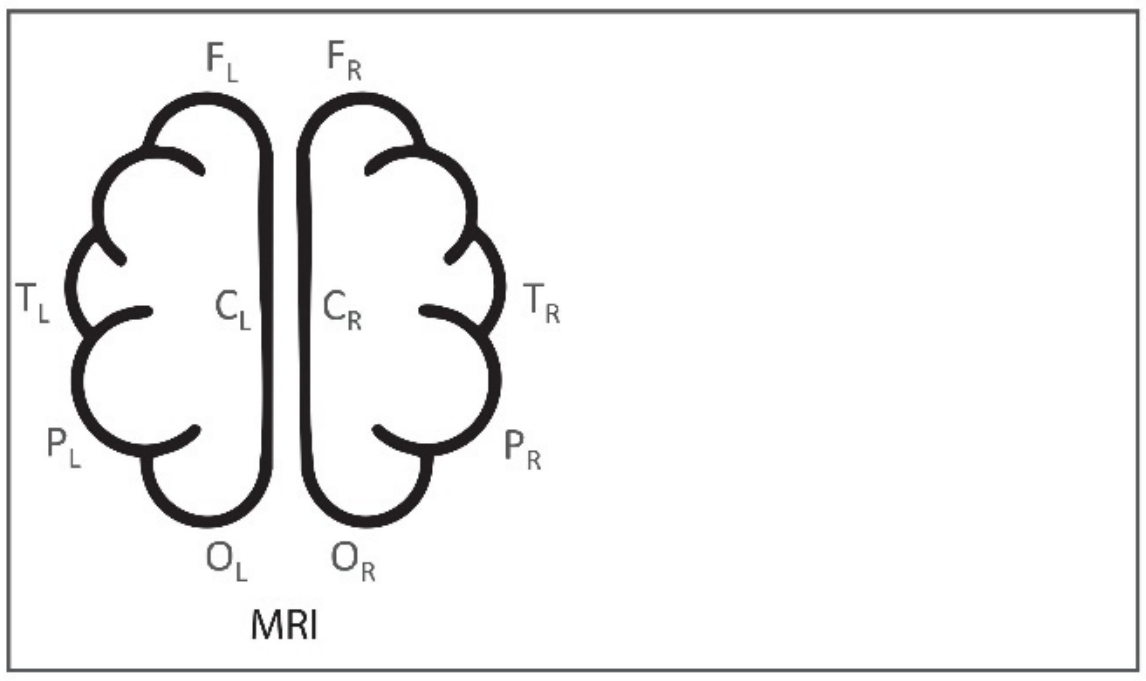

ictal	multifocal changing otherwise
interictal	interictal multifocality > ictal source(s)

patient 8

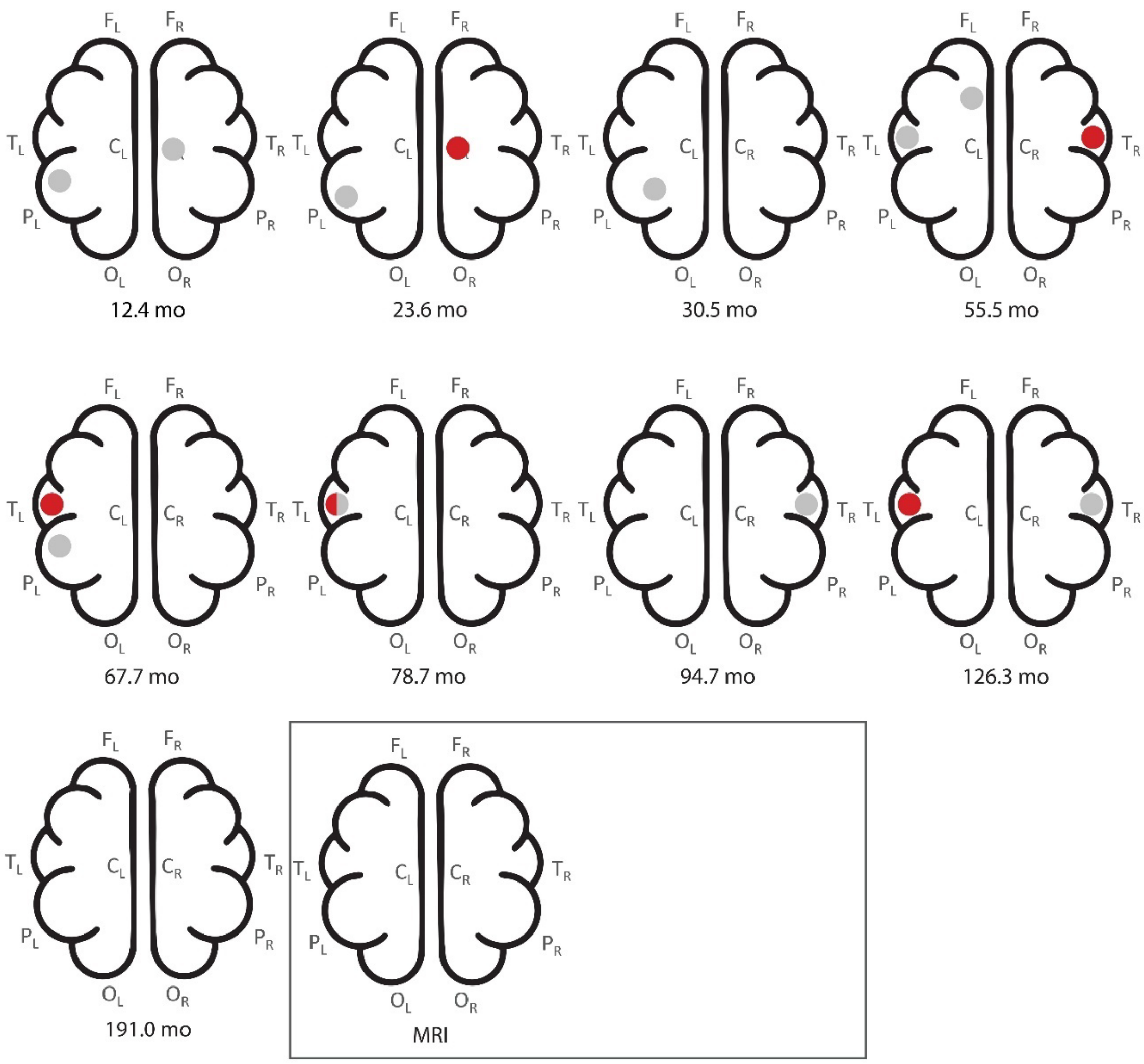

ictal	2 or more foci of at least 1 is consistent
interictal	interictal multifocality > ictal source(s)

patient 9

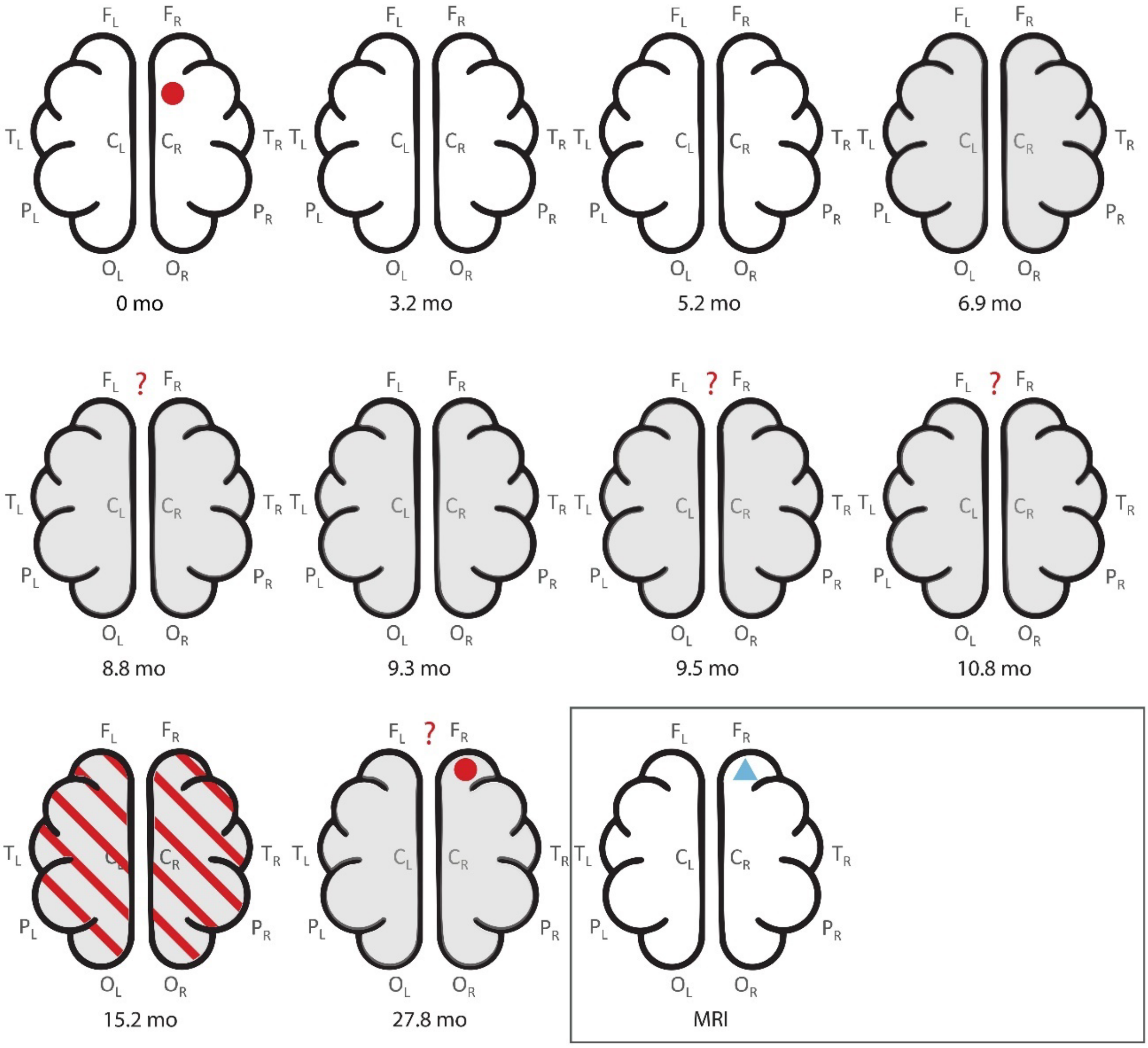

ictal	multifocal changing otherwise
interictal	interictal multifocality > ictal source(s)

patient 10

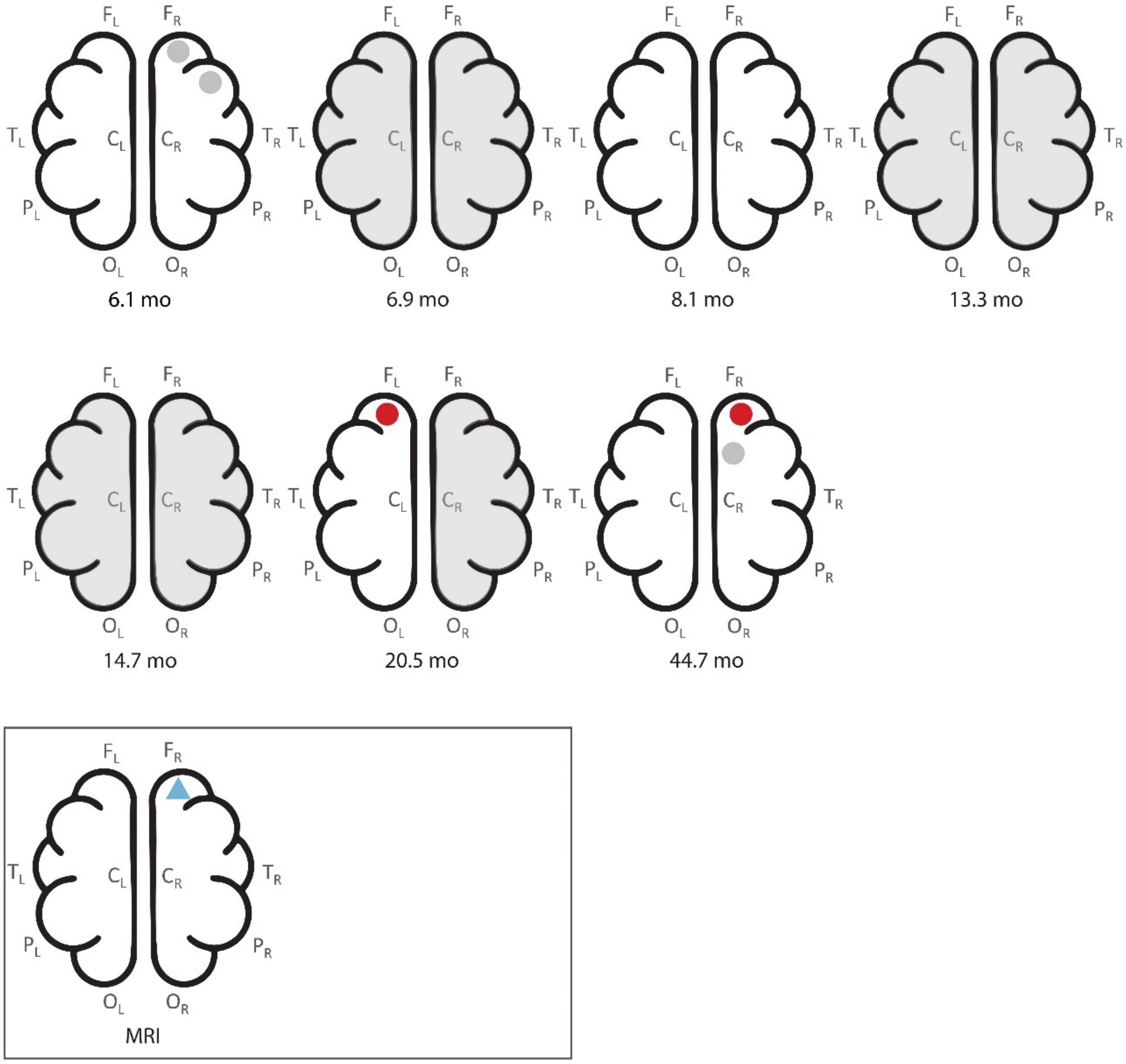

ictal	multifocal changing otherwise
interictal	interictal multifocality > ictal source(s)

patient 11

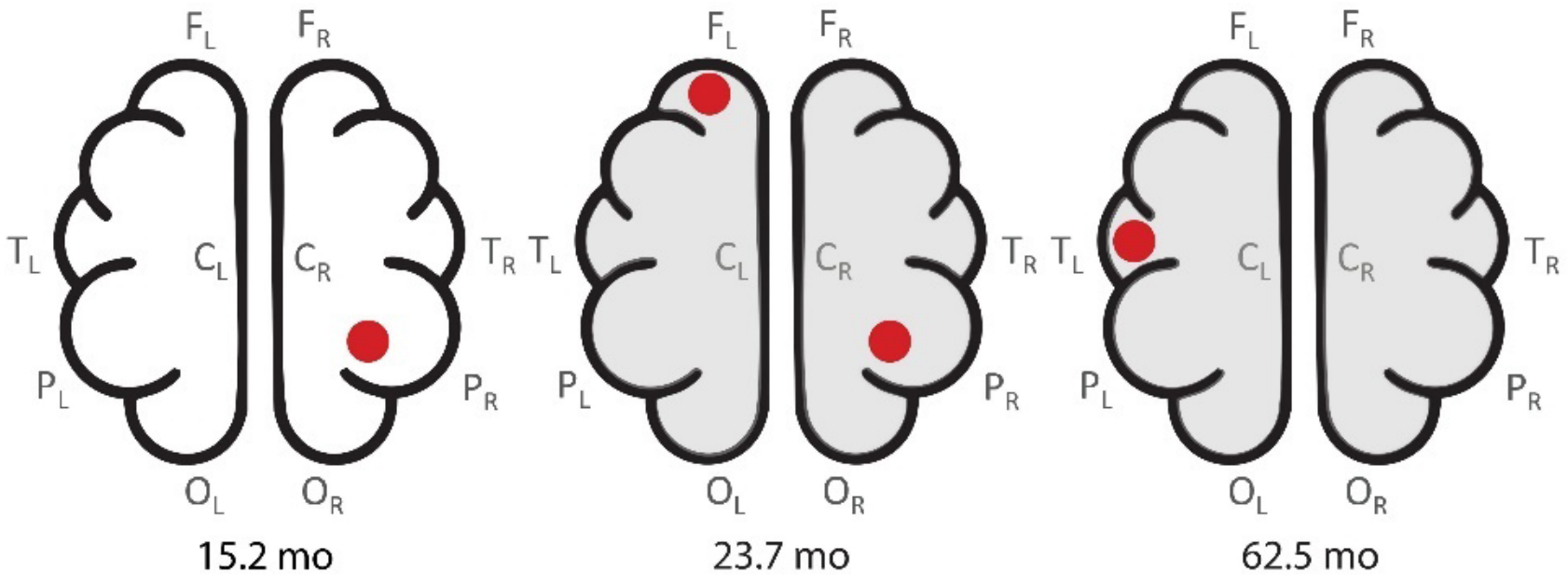

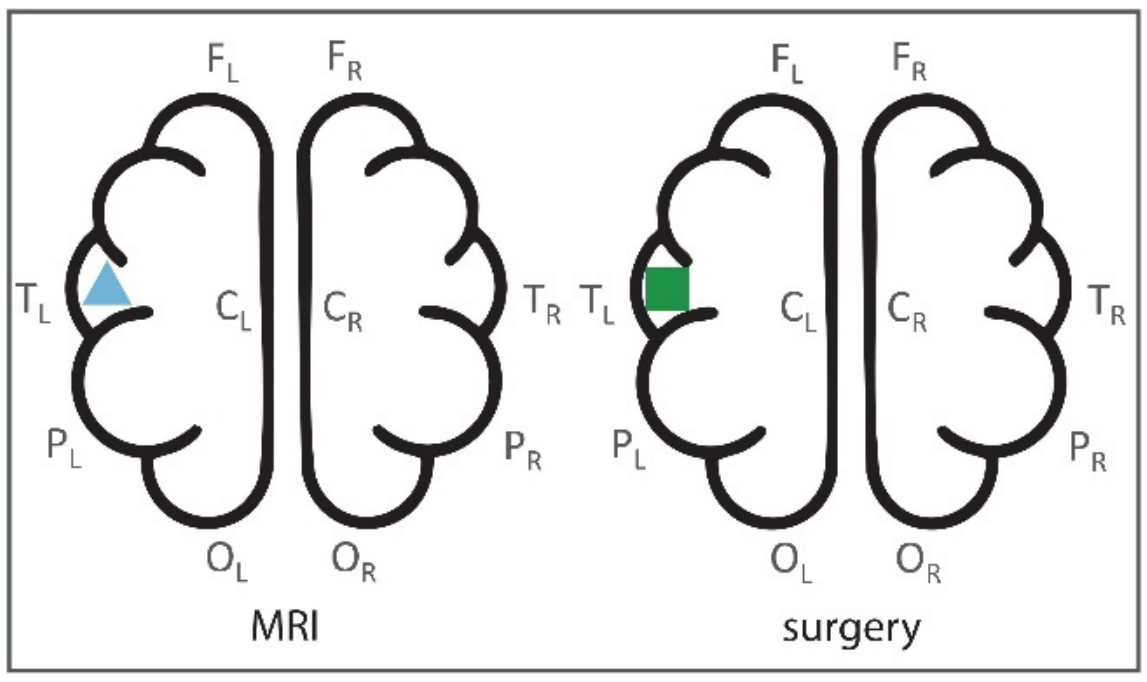

ictal	multifocal changing otherwise
interictal	interictal multifocality > ictal source(s)

patient 12

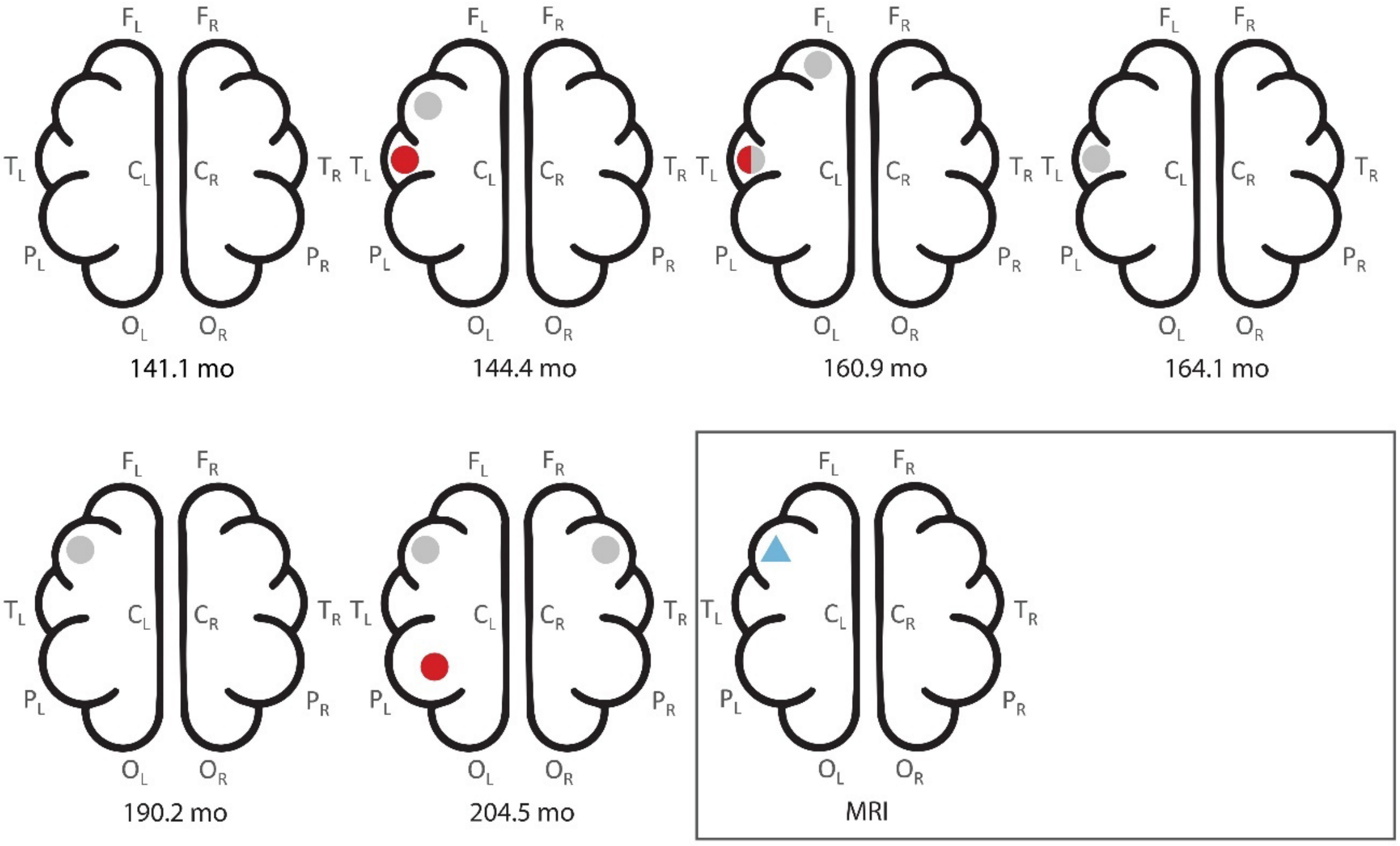

ictal	2 or more foci of which at least 1 is consistent
interictal	interictal multifocality > ictal source(s)

patient 13

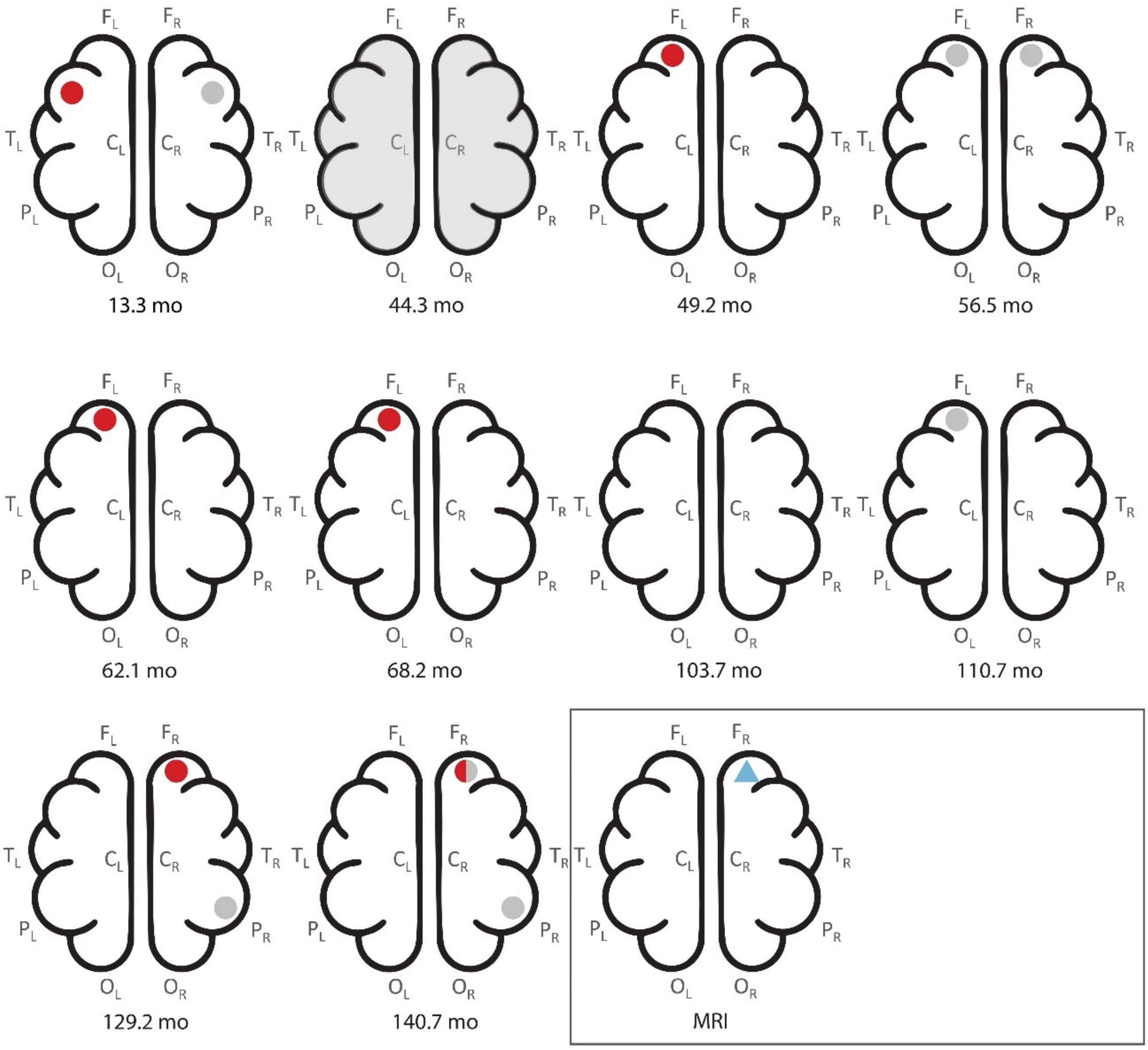

ictal	2 or more foci, each consistent
interictal	interictal multifocality > ictal source(s)

patient 14

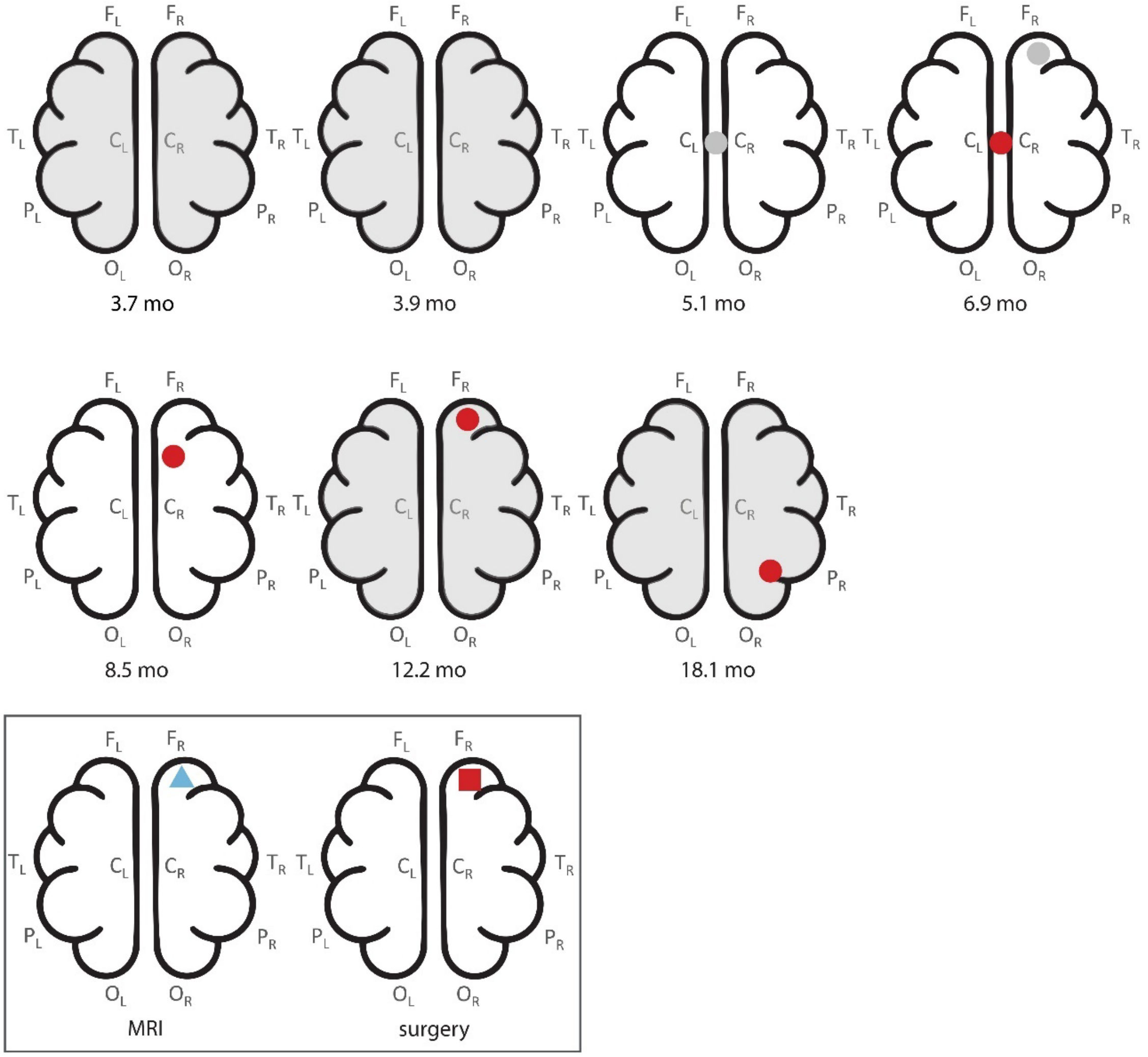

ictal	multifocal changing otherwise
interictal	interictal multifocality > ictal source(s)

patient 15

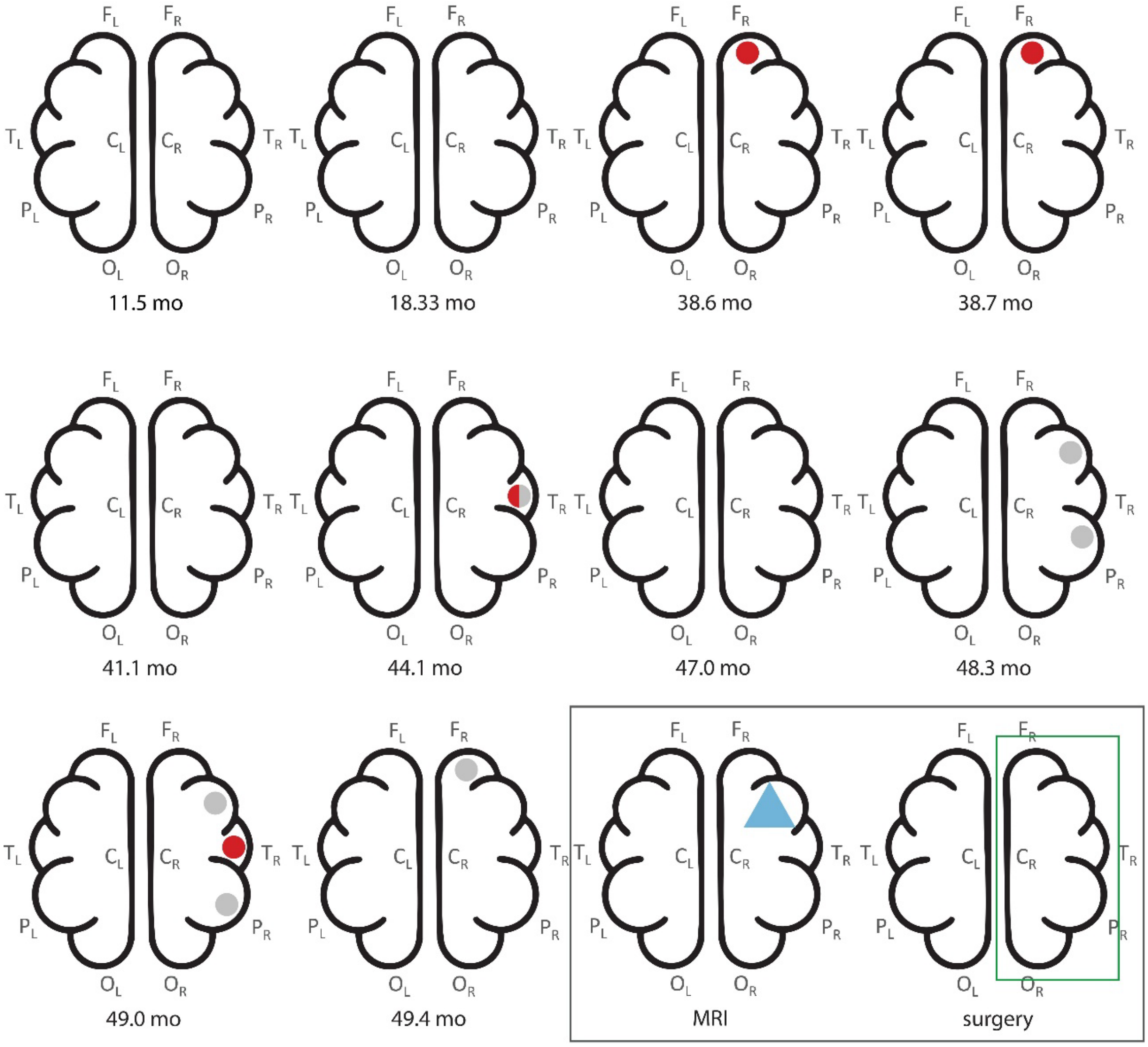

ictal	2 or more foci, each consistent
interictal	interictal multifocality > ictal source(s)

patient 16

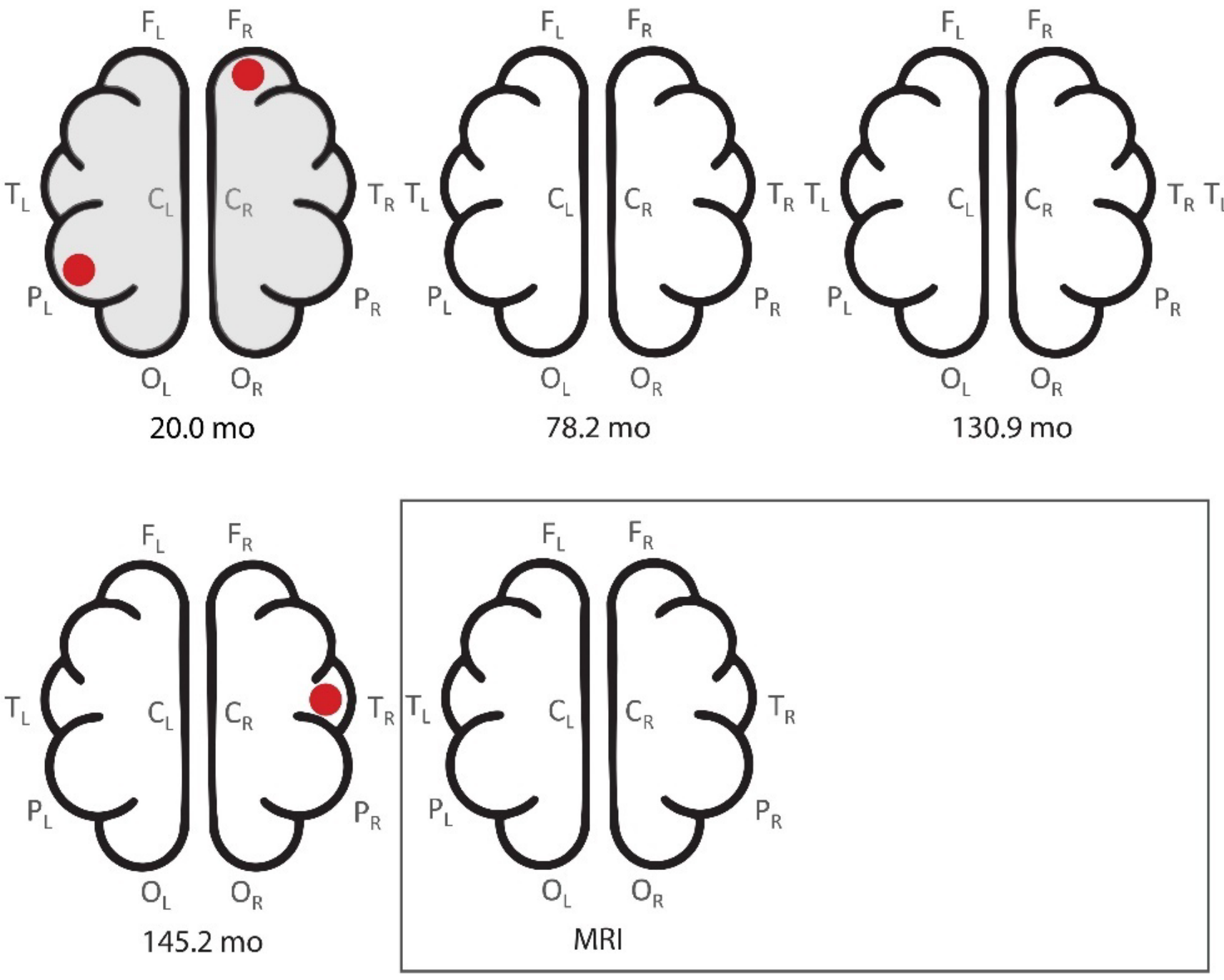

ictal	multifocal changing otherwise
interictal	interictal multifocality > ictal source(s)

patient 17

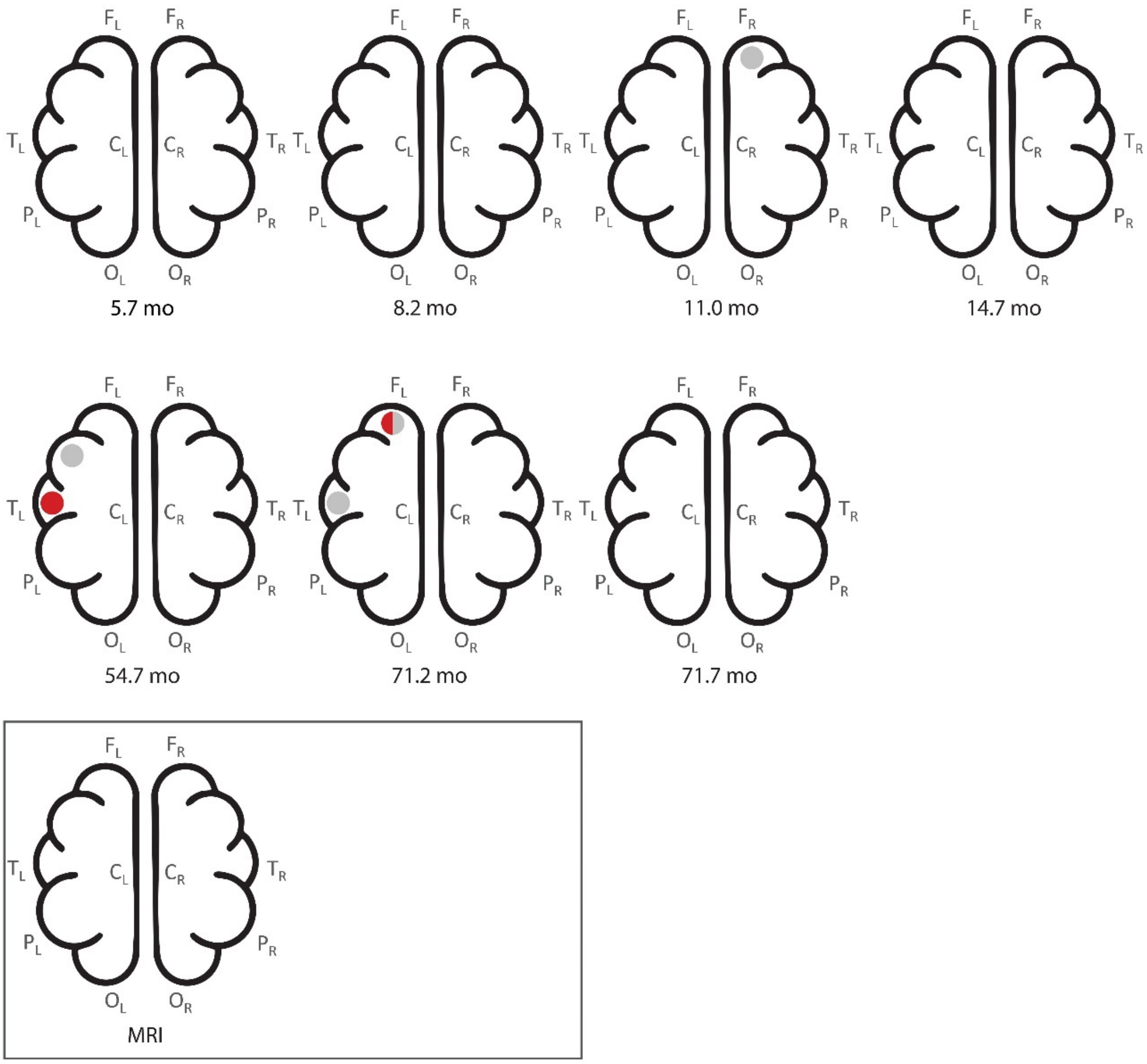

ictal	multifocal changing otherwise
interictal	interictal multifocality > ictal source(s)

patient 18

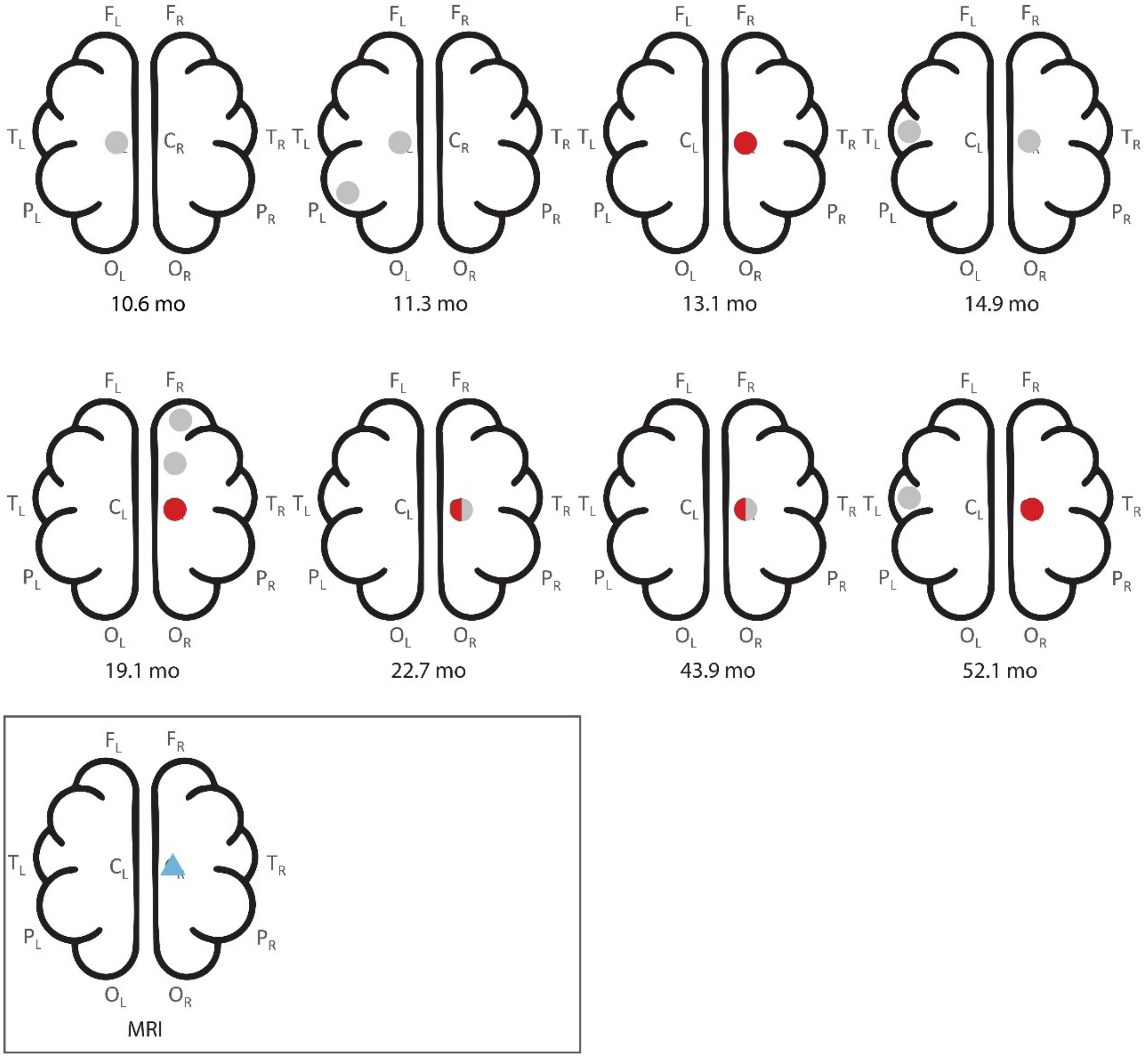

ictal	single focus, consistent
interictal	interictal multifocality > ictal source(s)

patient 19

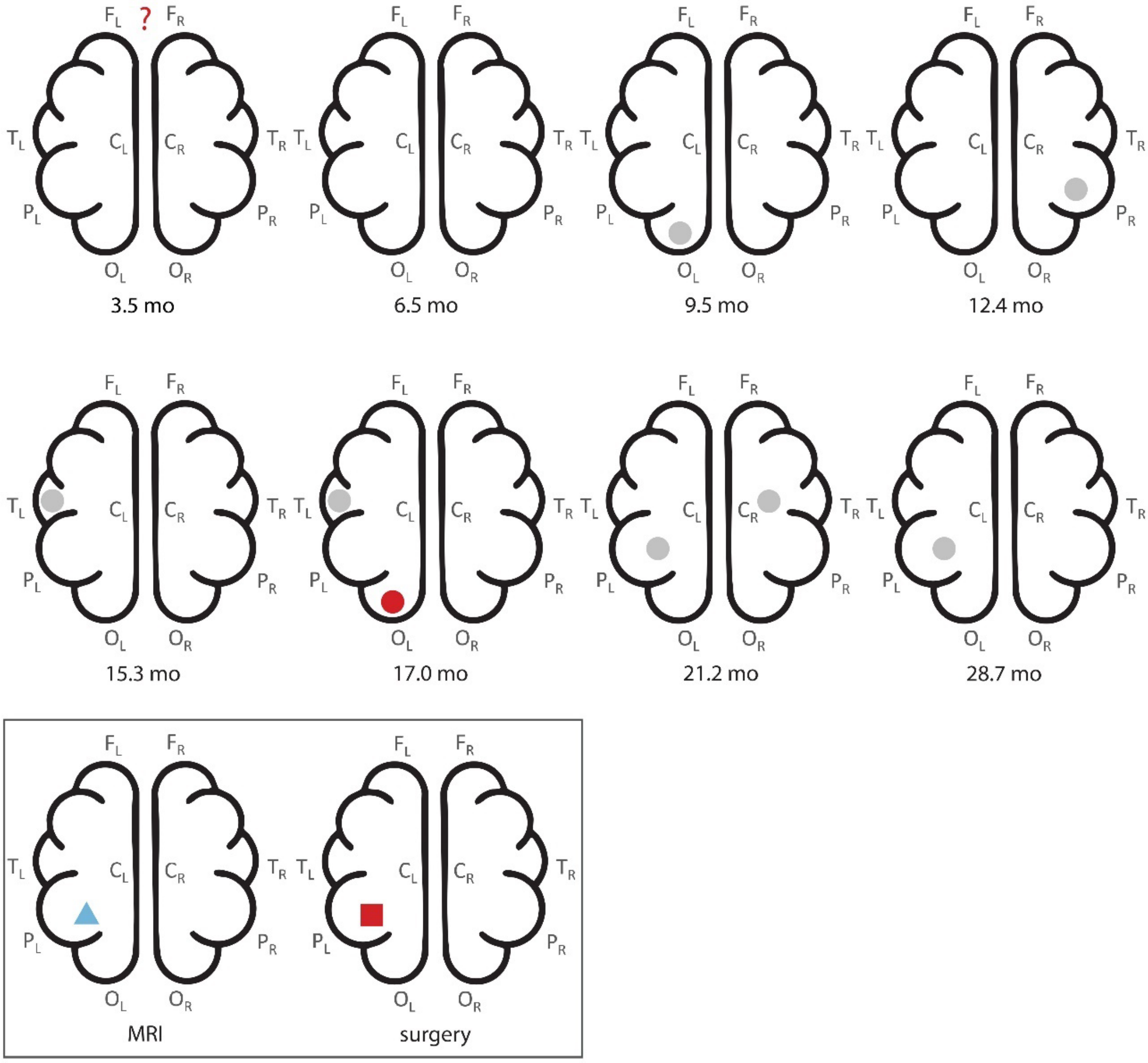

ictal	starting unclear/diffuse generalised to unifocal
interictal	interictal multifocality > ictal source(s)

patient 20

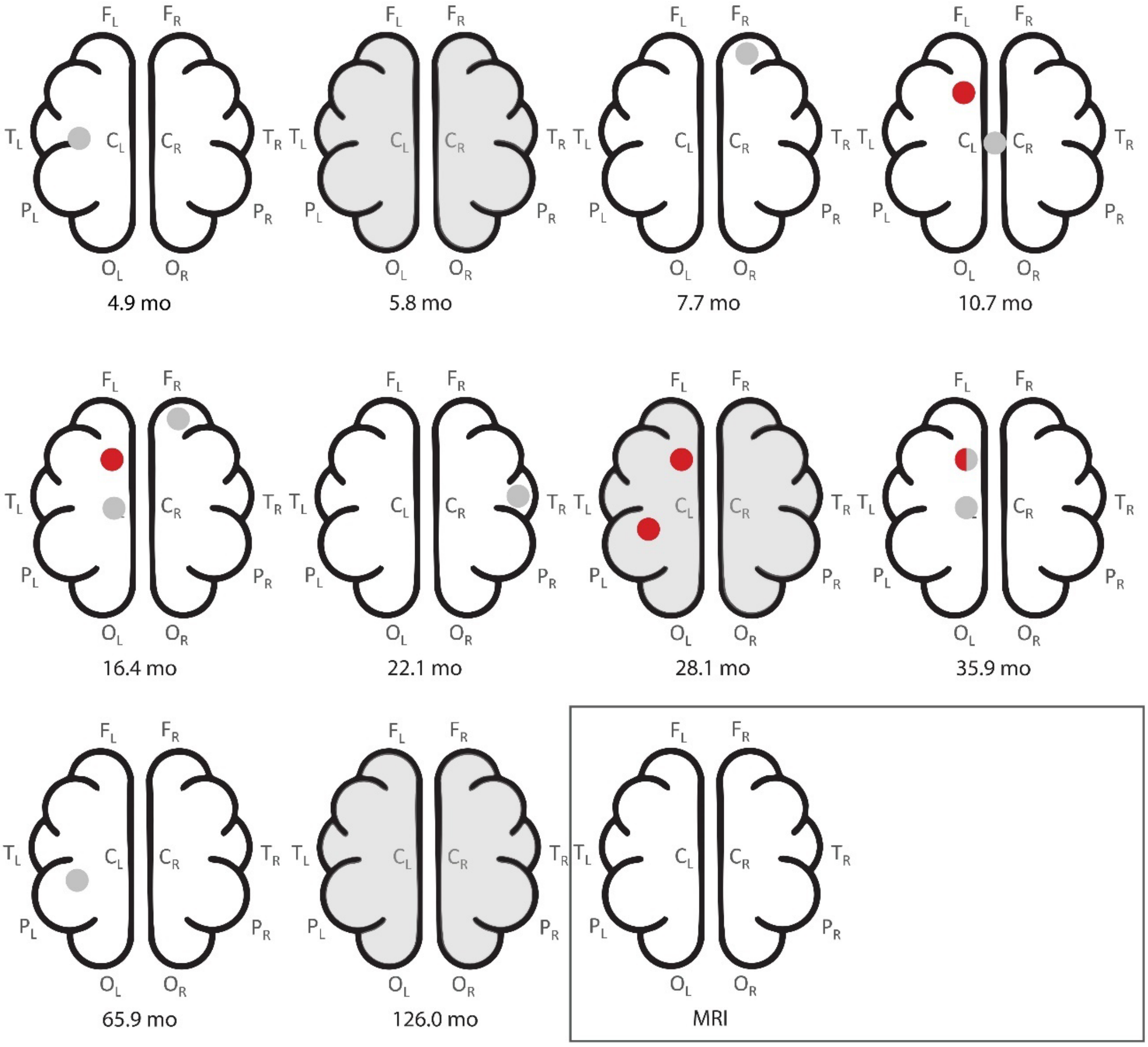

ictal	2 or more foci of which at least 1 is consistent
interictal	interictal multifocality > ictal source(s)

patient 21

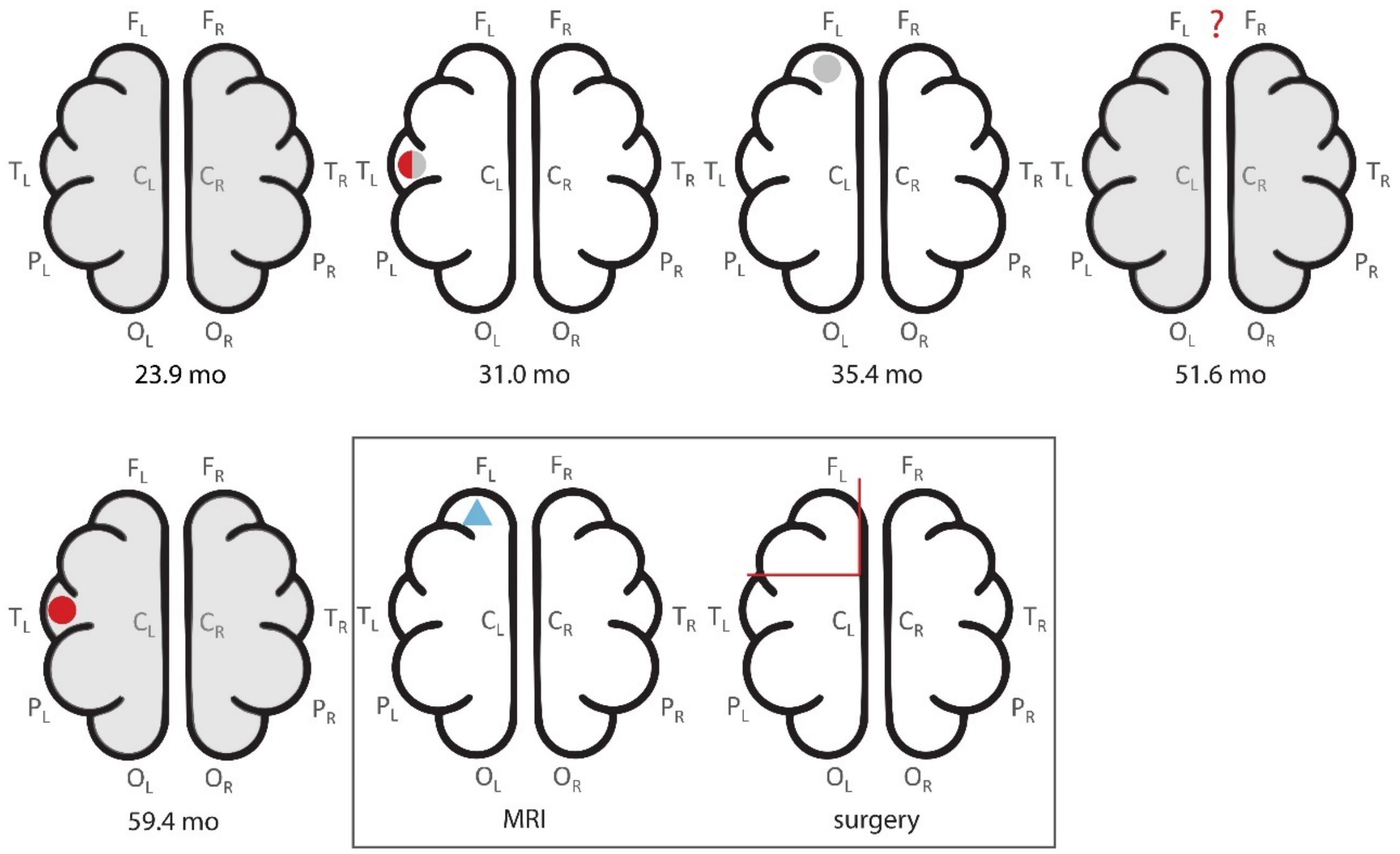

ictal	2 or more foci of which at least 1 is consistent
interictal	interictal multifocality > ictal source(s)

patient 22

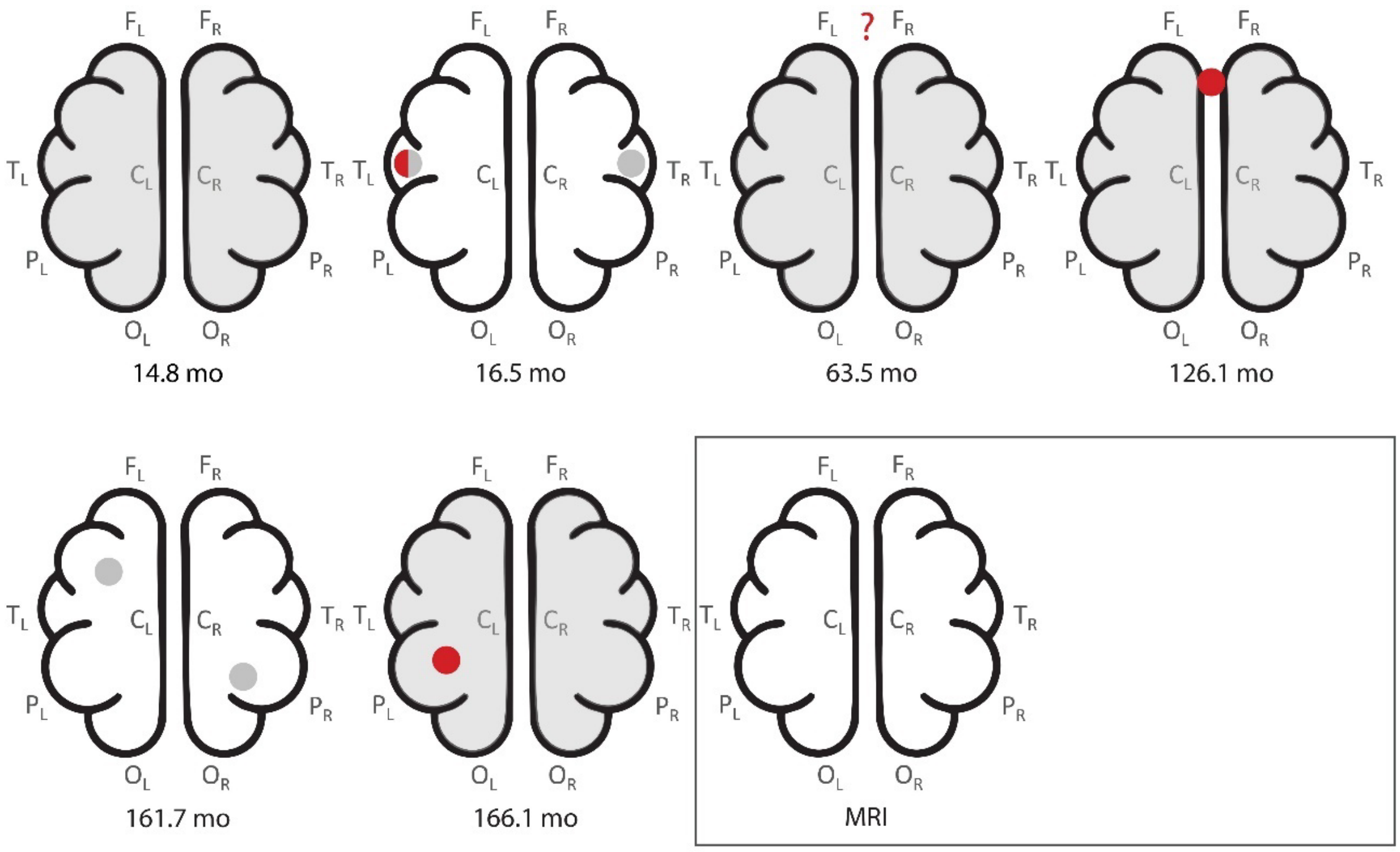

ictal	multifocal changing otherwise
interictal	interictal multifocality > ictal source(s)

patient 23

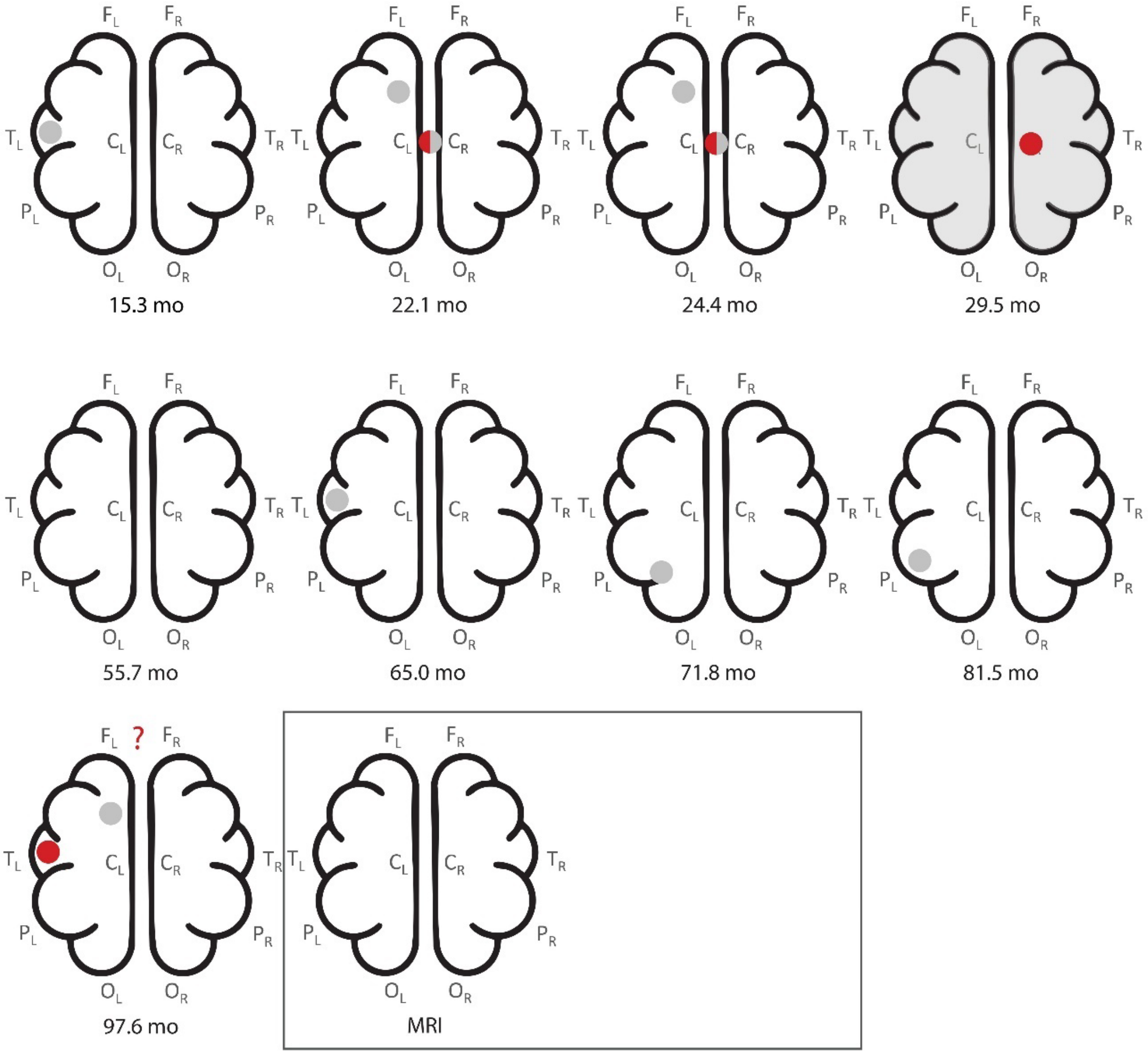

ictal	2 or more foci of which at least 1 is consistent
interictal	interictal multifocality > ictal source(s)

patient 24

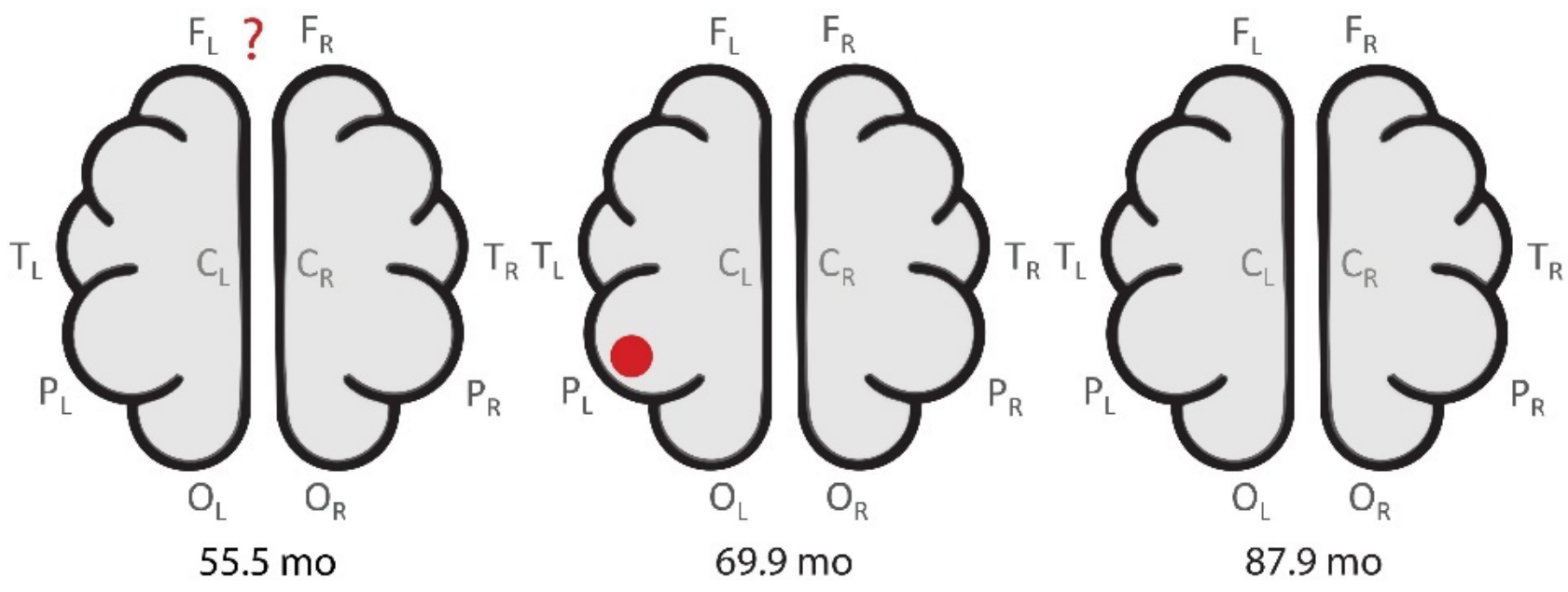

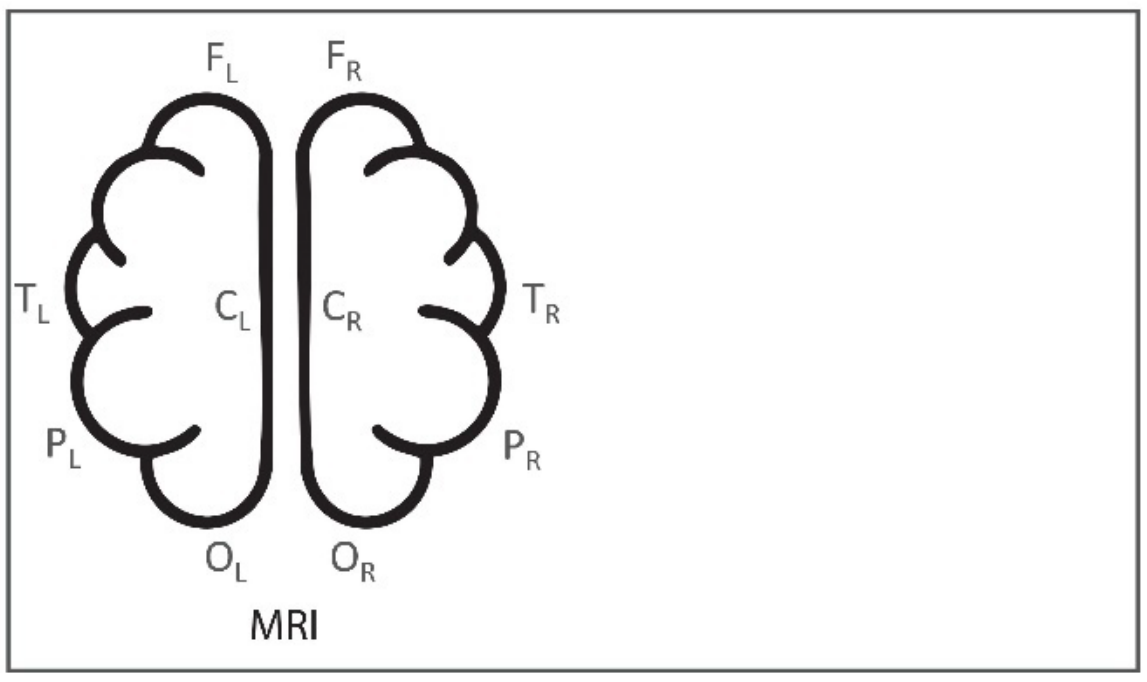

ictal	starting unclear/diffuse generalised to unifocal
interictal	interictal multifocality > ictal source(s)

patient 25

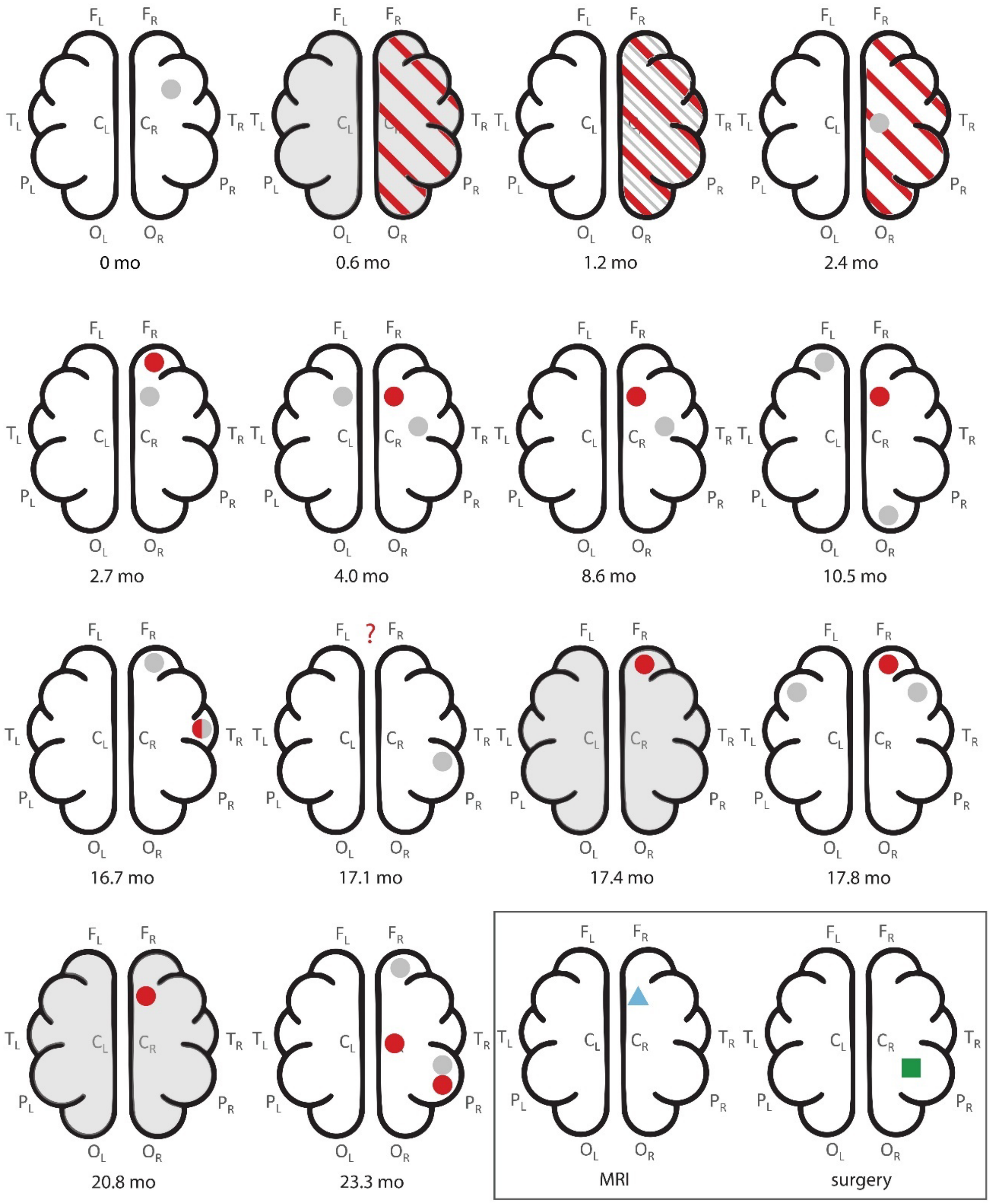

ictal	multifocal changing otherwise
interictal	interictal multifocality > ictal source(s)

patient 26

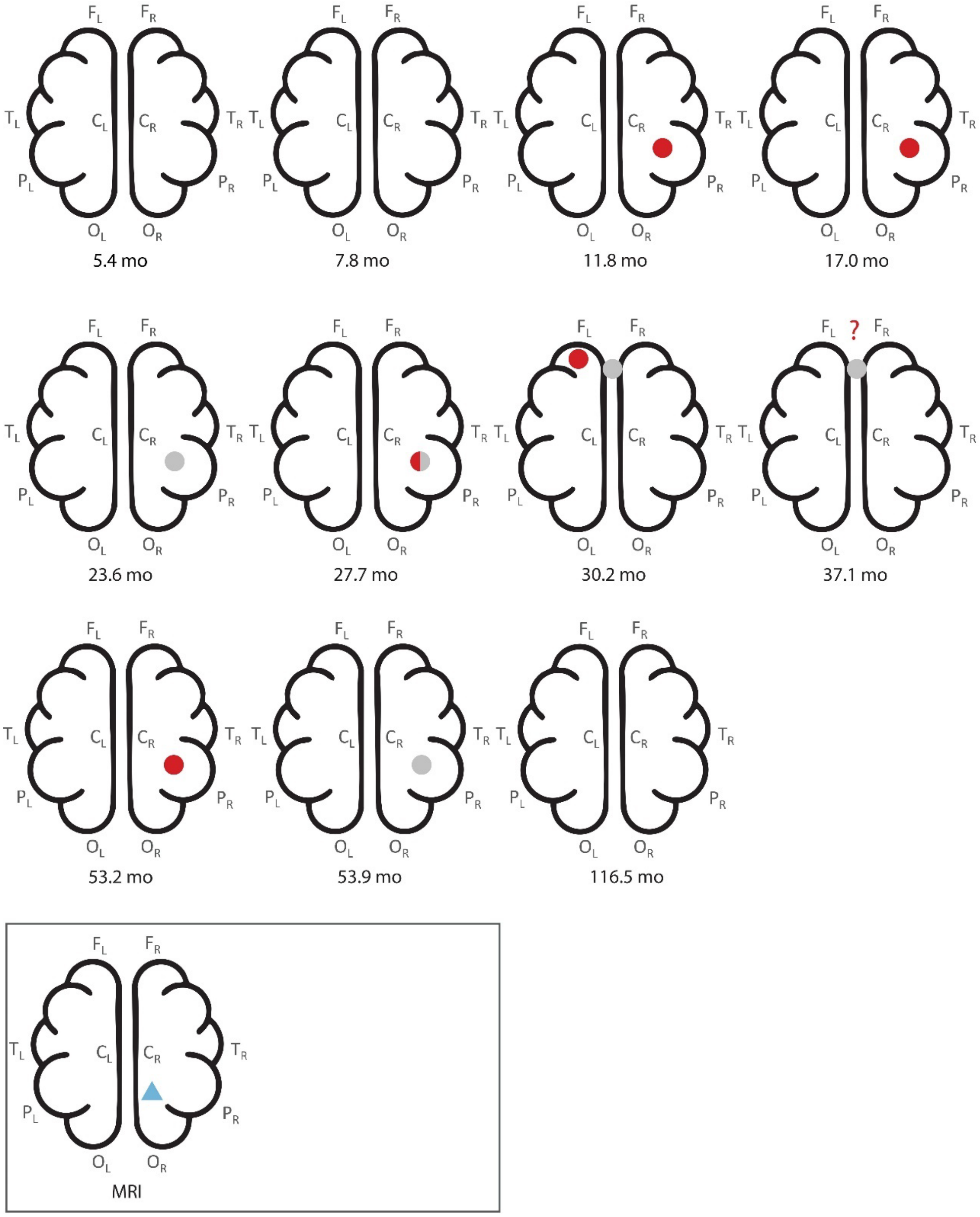

ictal	2 or more foci of which at least 1 is consistent
interictal	all interictal sources concordant with ictals

patient 27

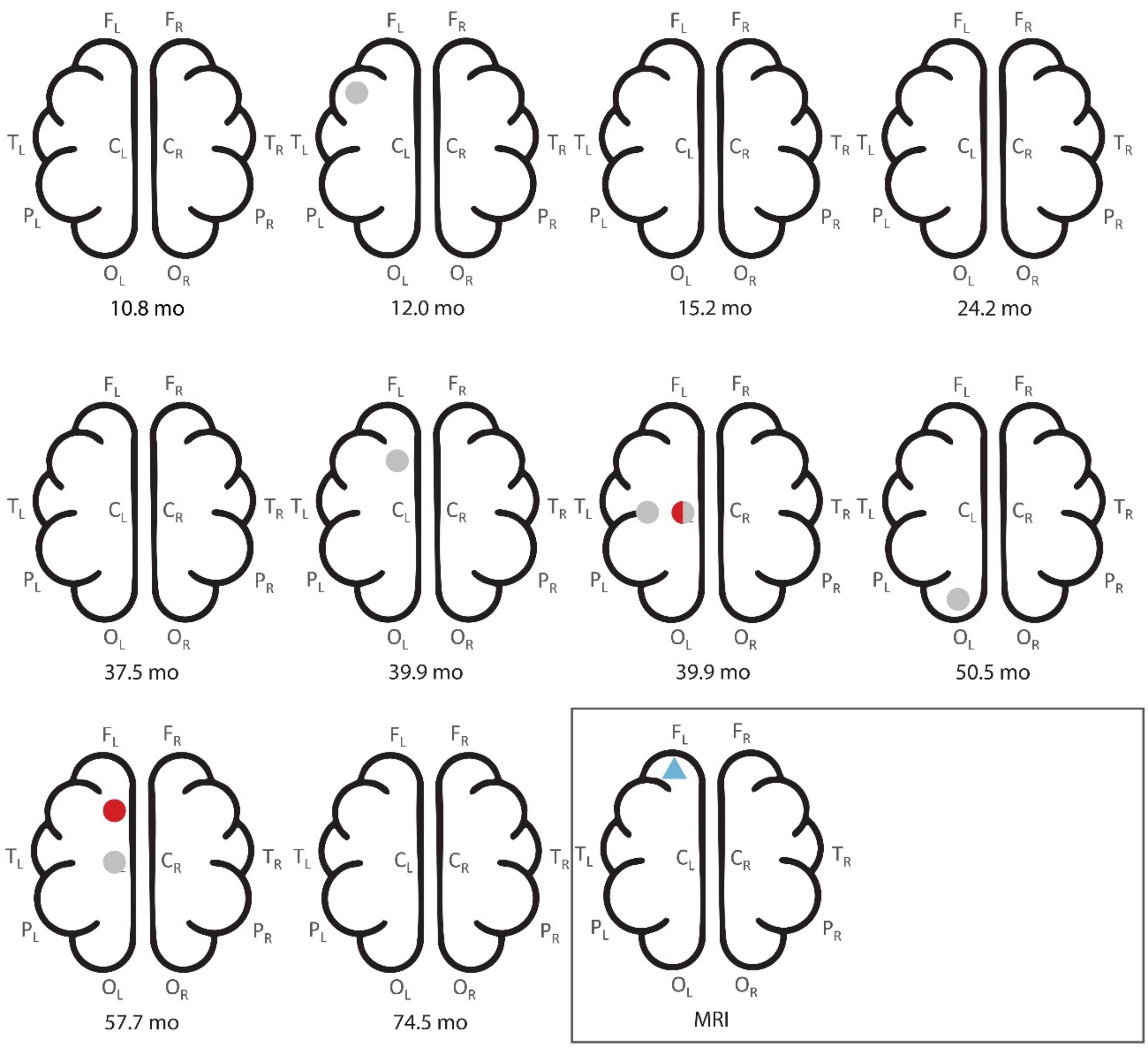

ictal	single focus, consistent
interictal	interictal multifocality > ictal source(s)

patient 28

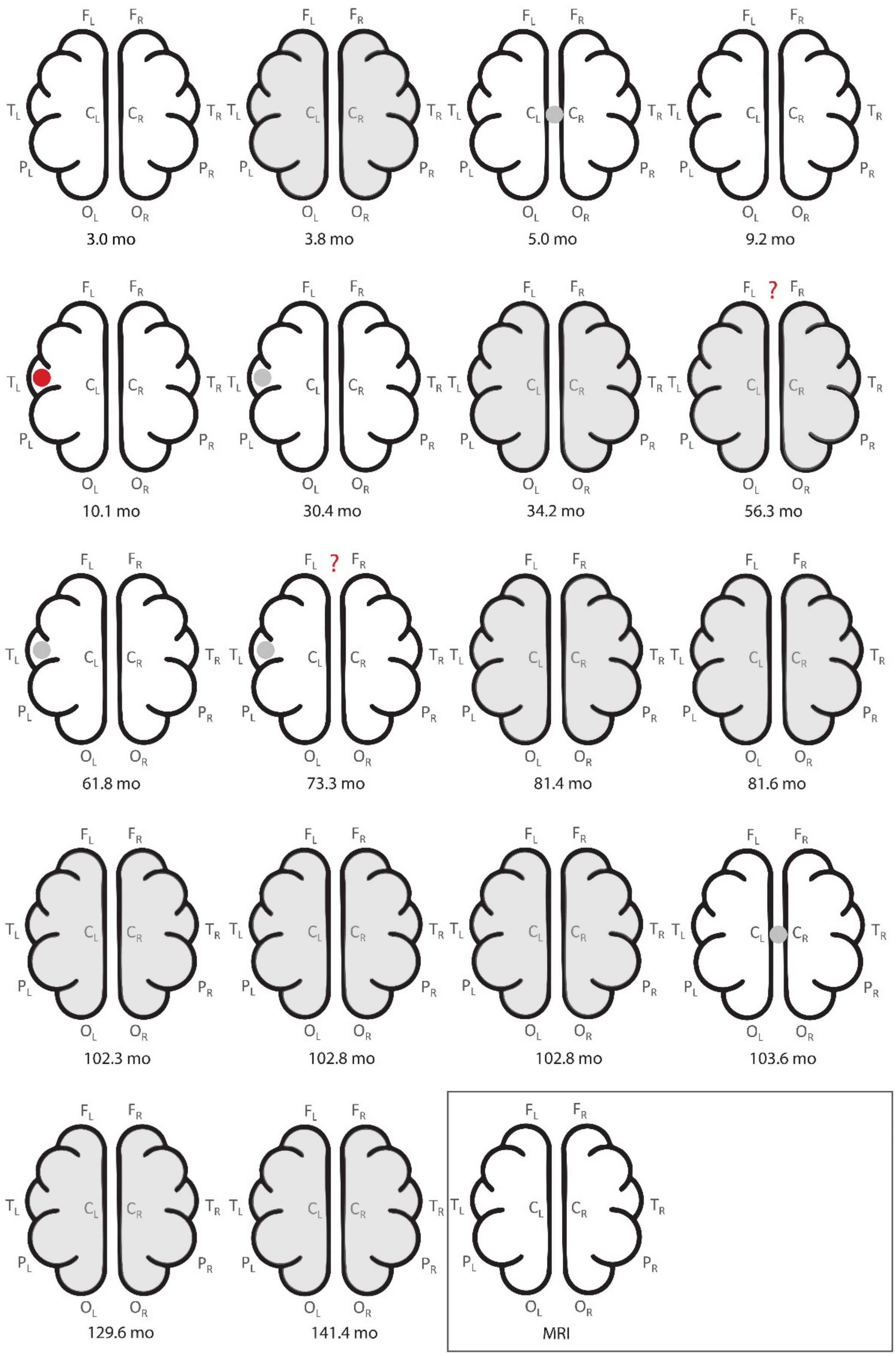

ictal	multifocal changing otherwise
interictal	interictal multifocality > ictal source(s)

patient 29

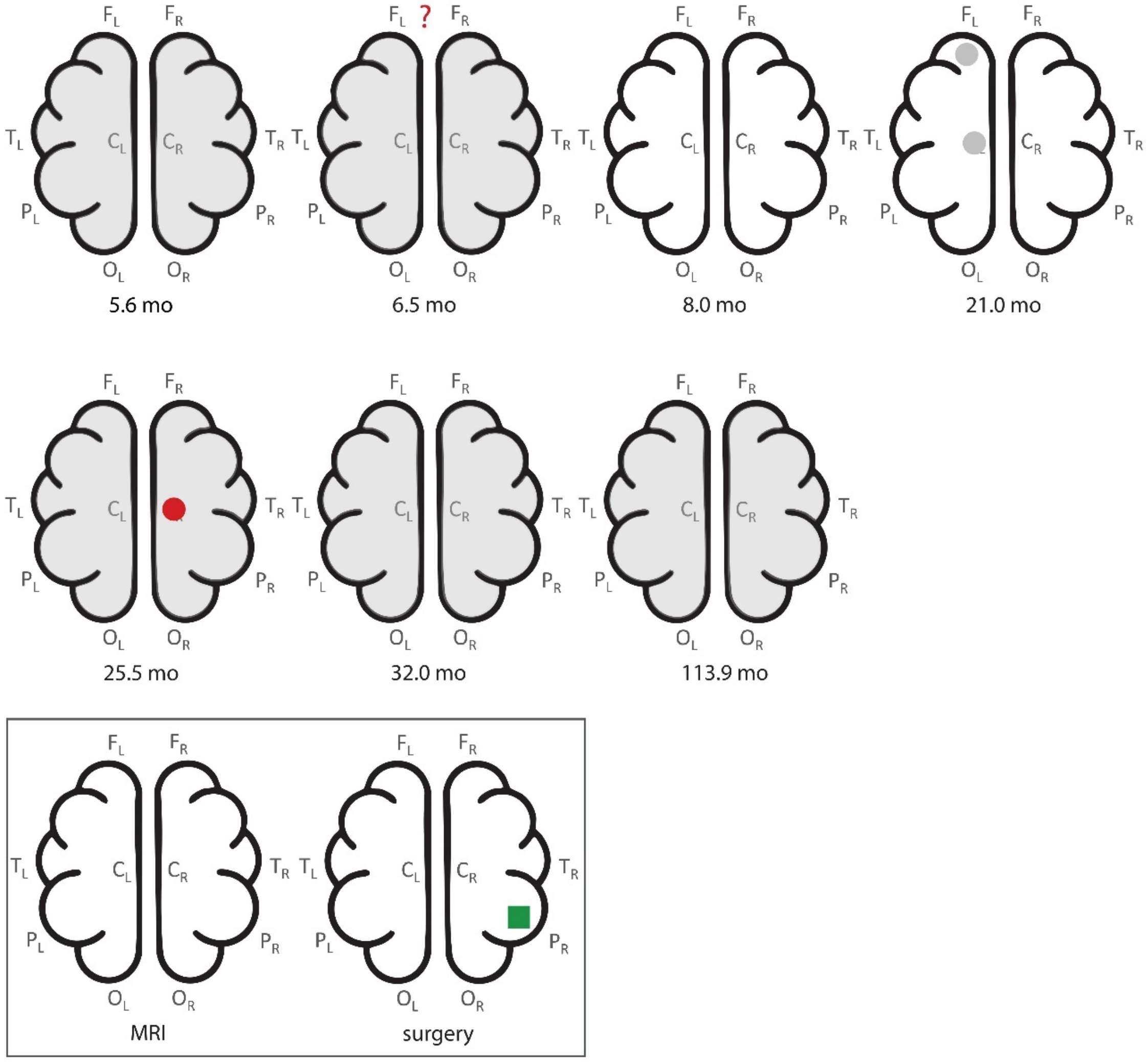

ictal	starting unclear/diffuse generalised to unifocal
interictal	interictal multifocality > ictal source(s)

patient 30

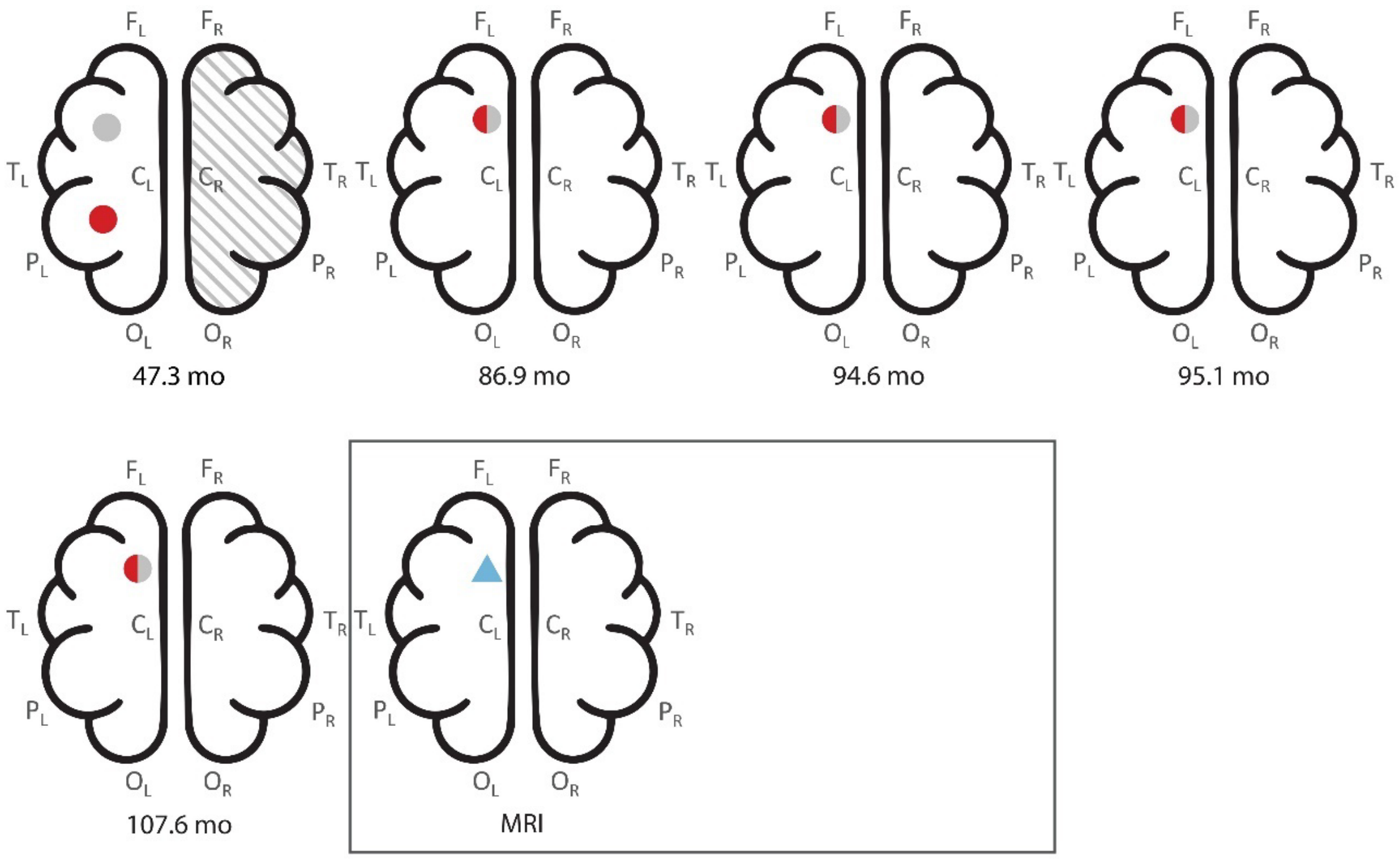

ictal	2 or more foci, each consistent
interictal	interictal multifocality > ictal source(s)

patient 31

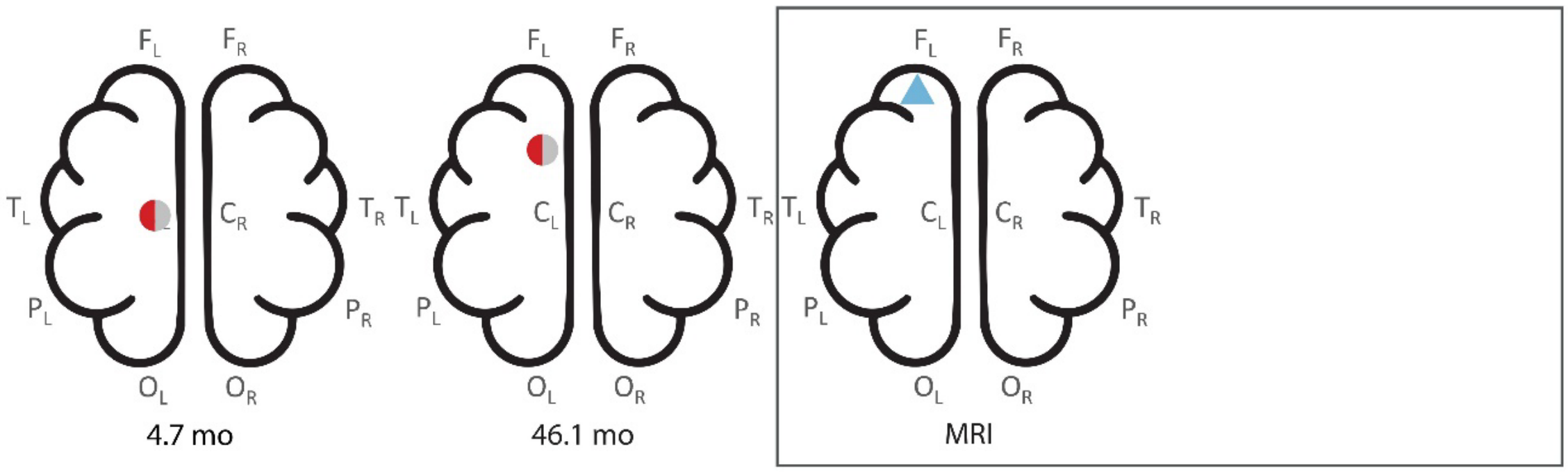

ictal	single focus, consistent
interictal	concordant interictal correlate

patient 32

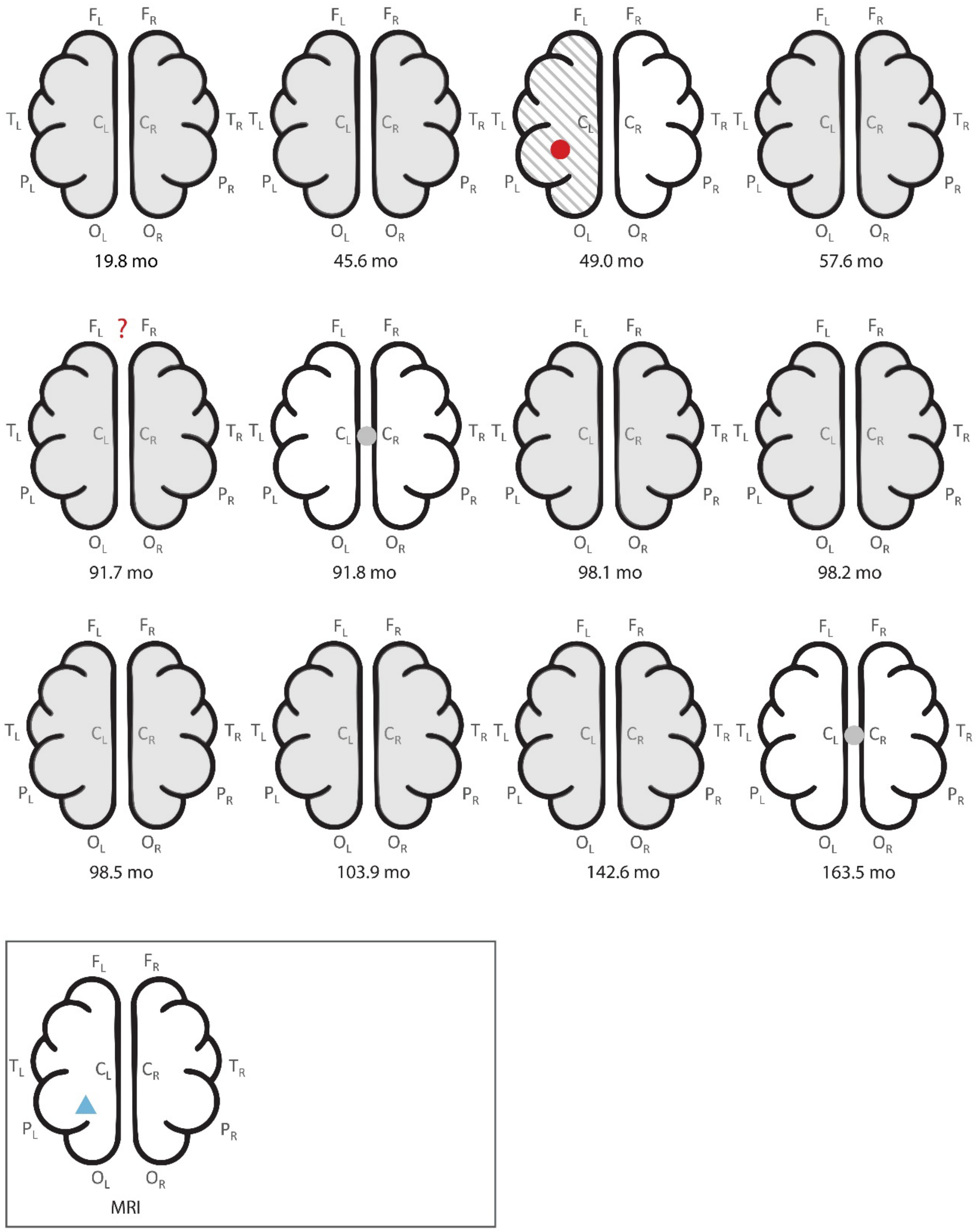

ictal	multifocal changing otherwise
interictal	interictal multifocality > ictal source(s)

patient 33

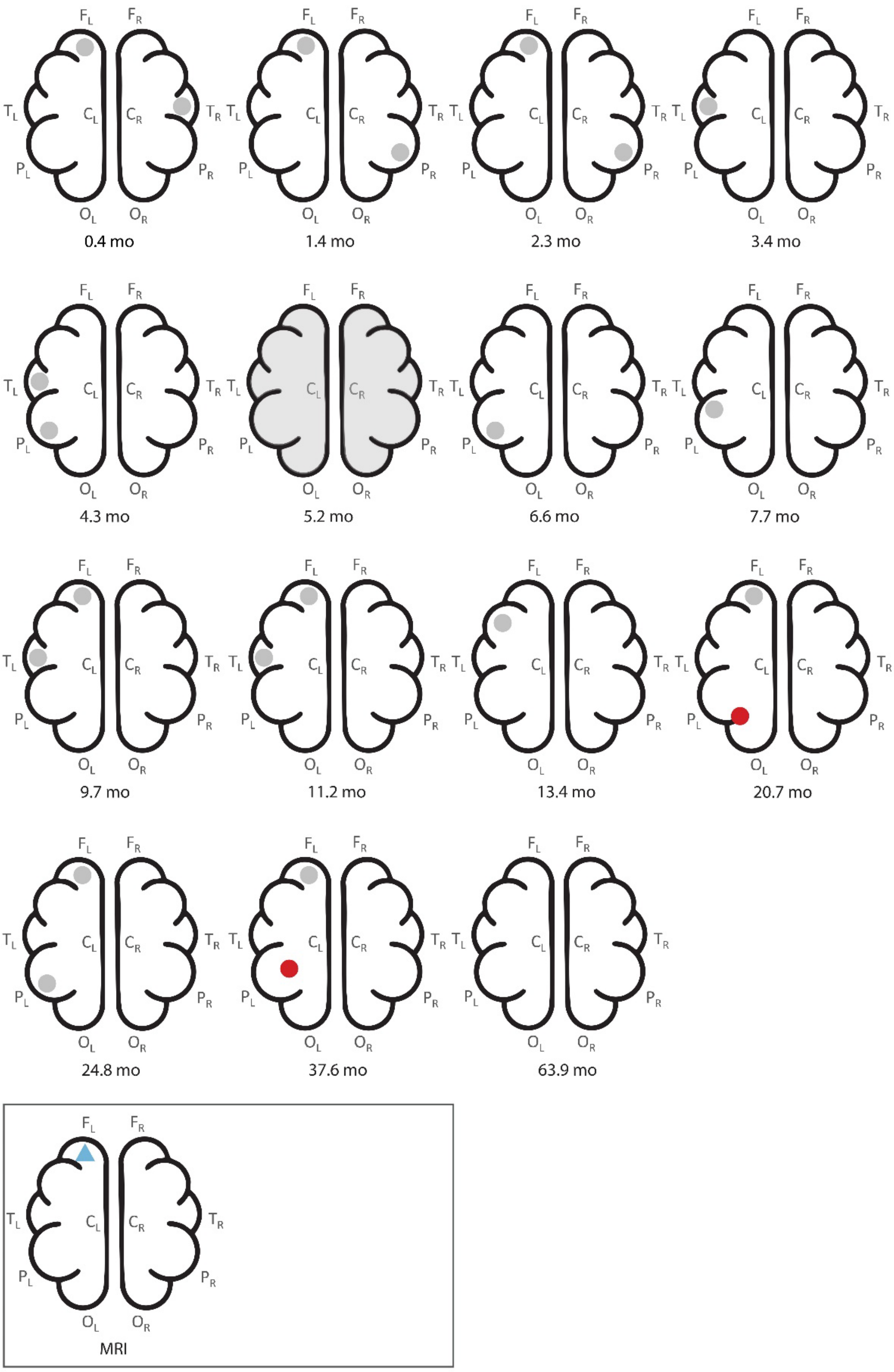

ictal	single focus, consistent
interictal	interictal multifocality > ictal source(s)

patient 34

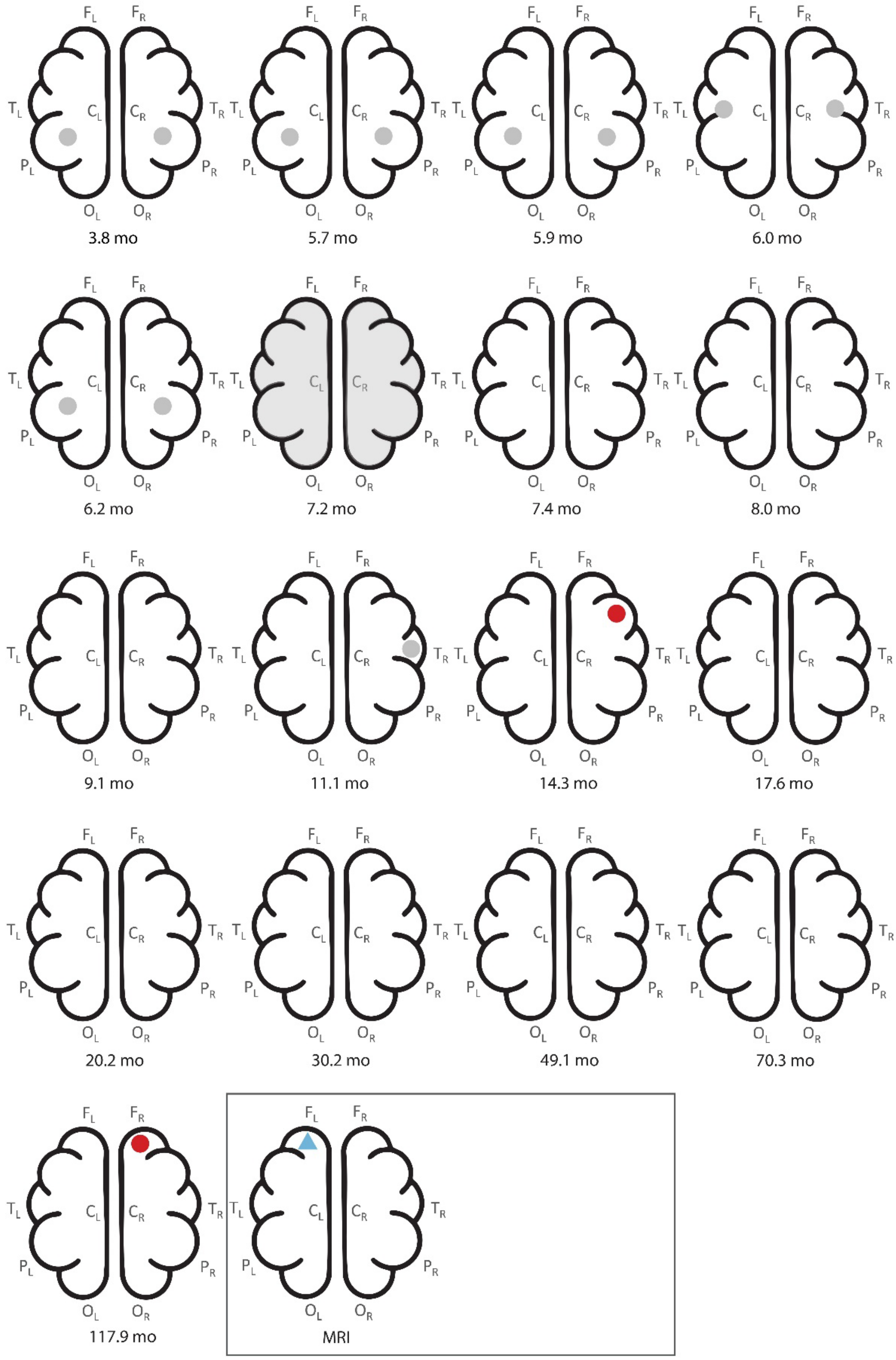

ictal	single focus, consistent
interictal	interictal multifocality > ictal source(s)

patient 35

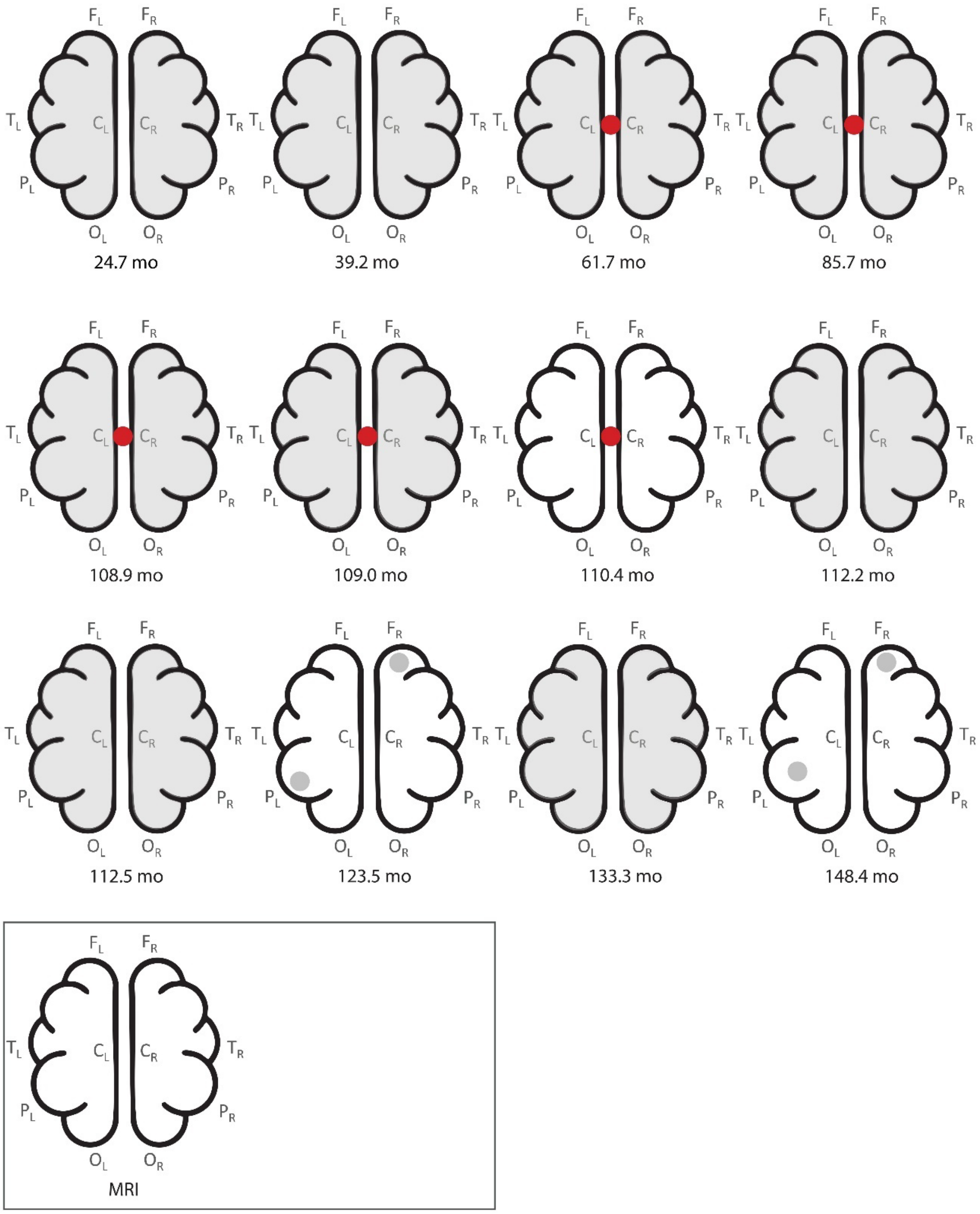

ictal	single focus, consistent
interictal	interictal multifocality > ictal source(s)

patient 36

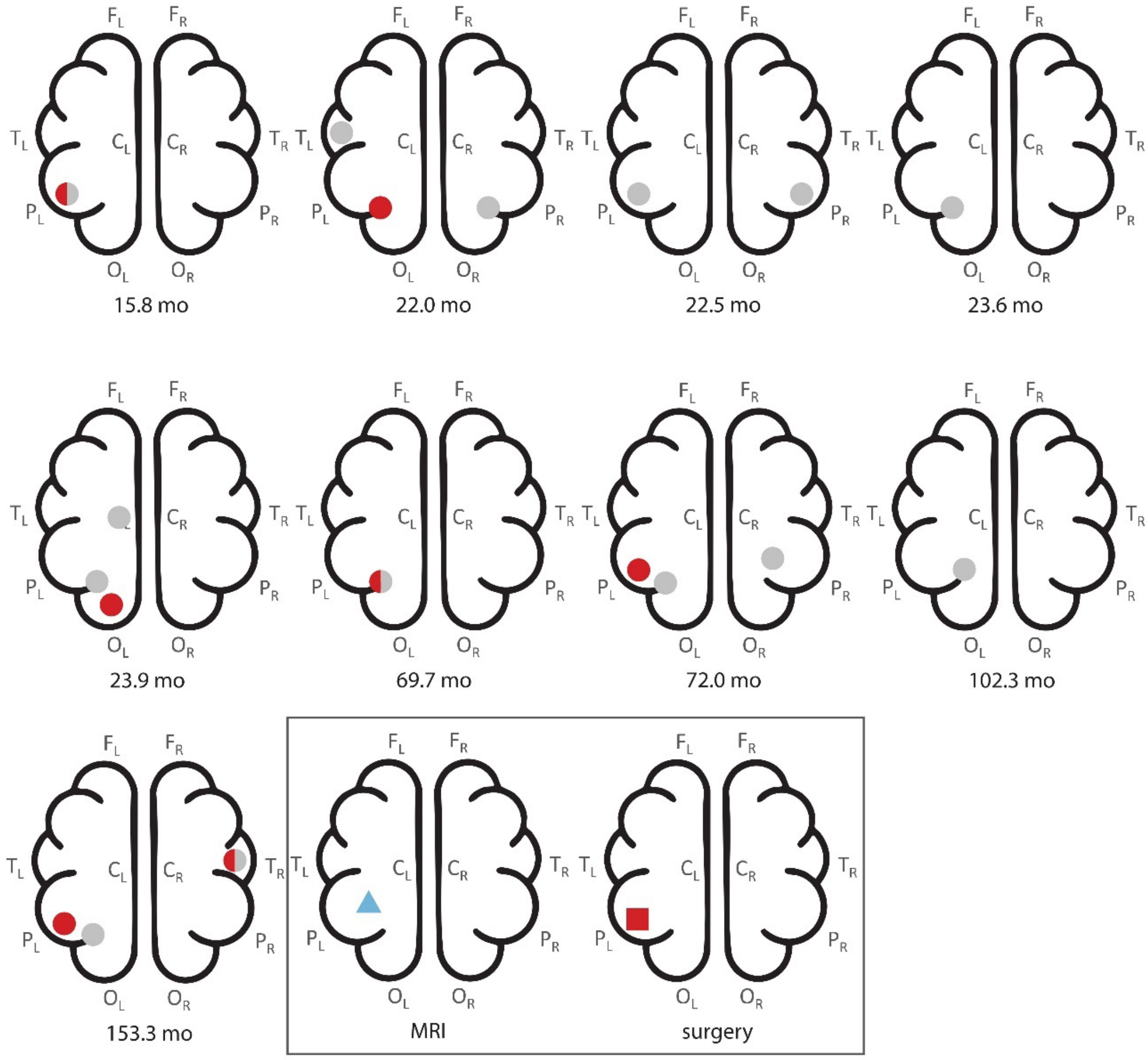

ictal	2 or more foci of which at least 1 is consistent
interictal	interictal multifocality > ictal source(s)

patient 37

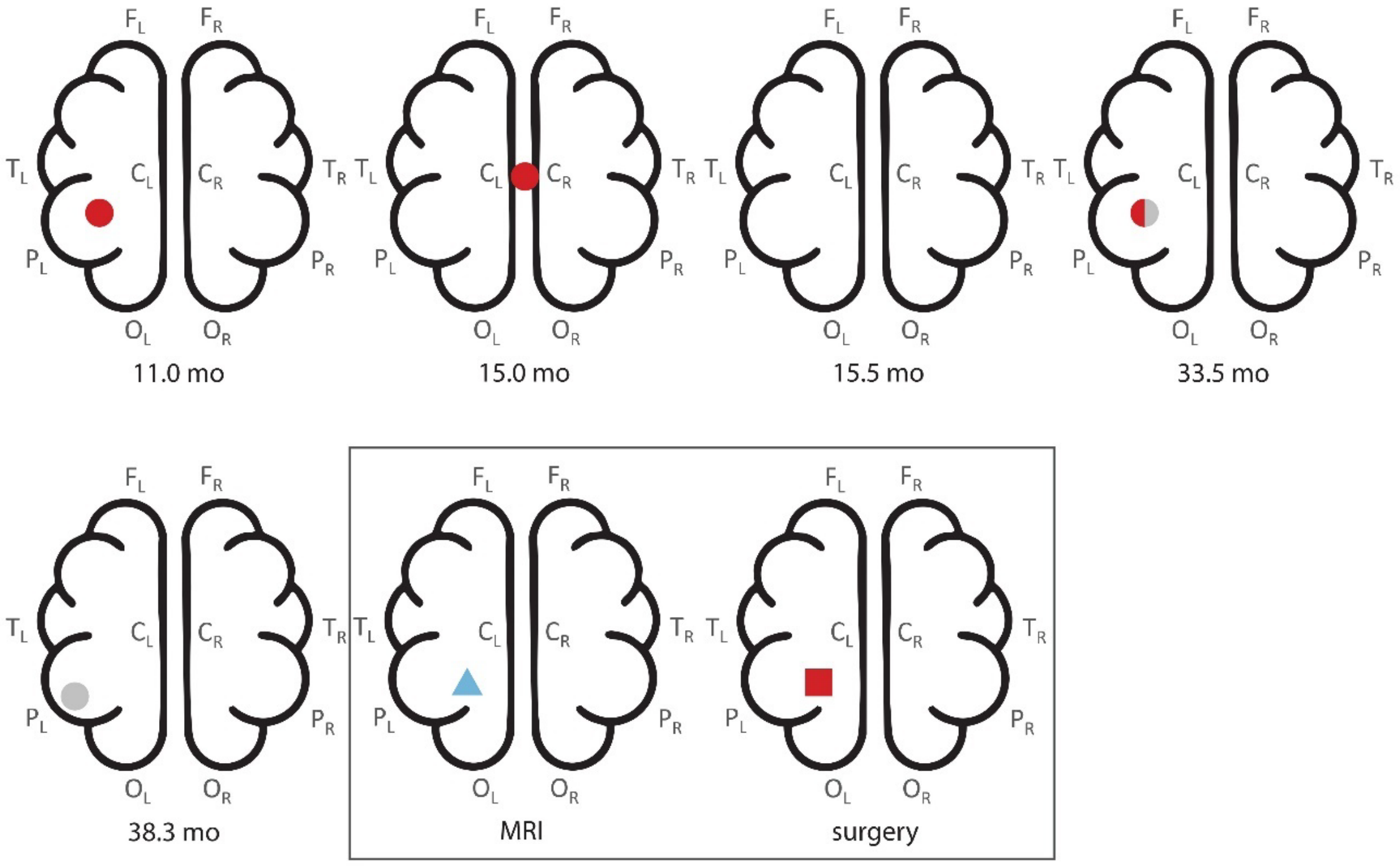

ictal	2 or more foci of which at least 1 is consistent
interictal	all interictal sources concordant with ictals

patient 38

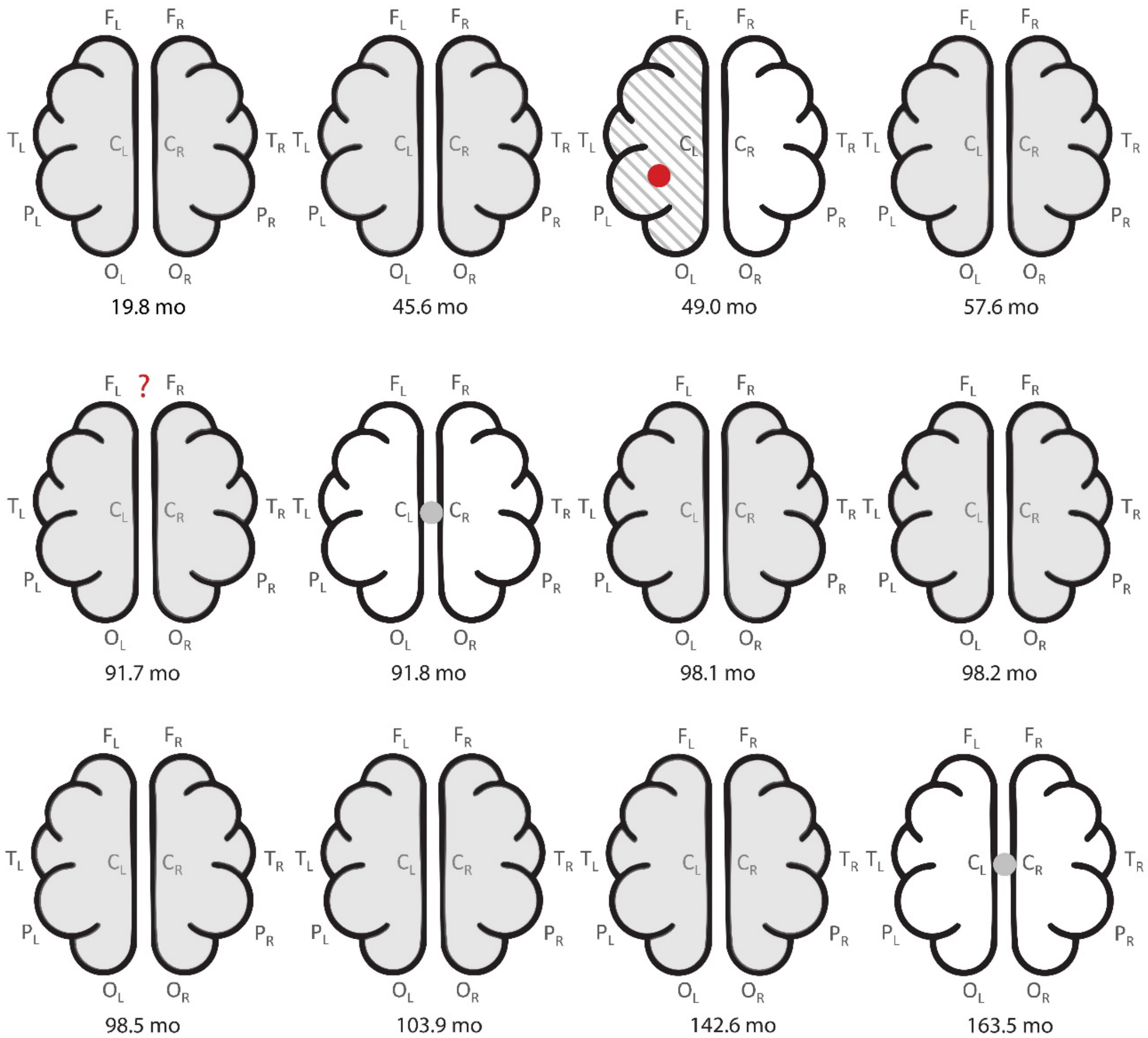

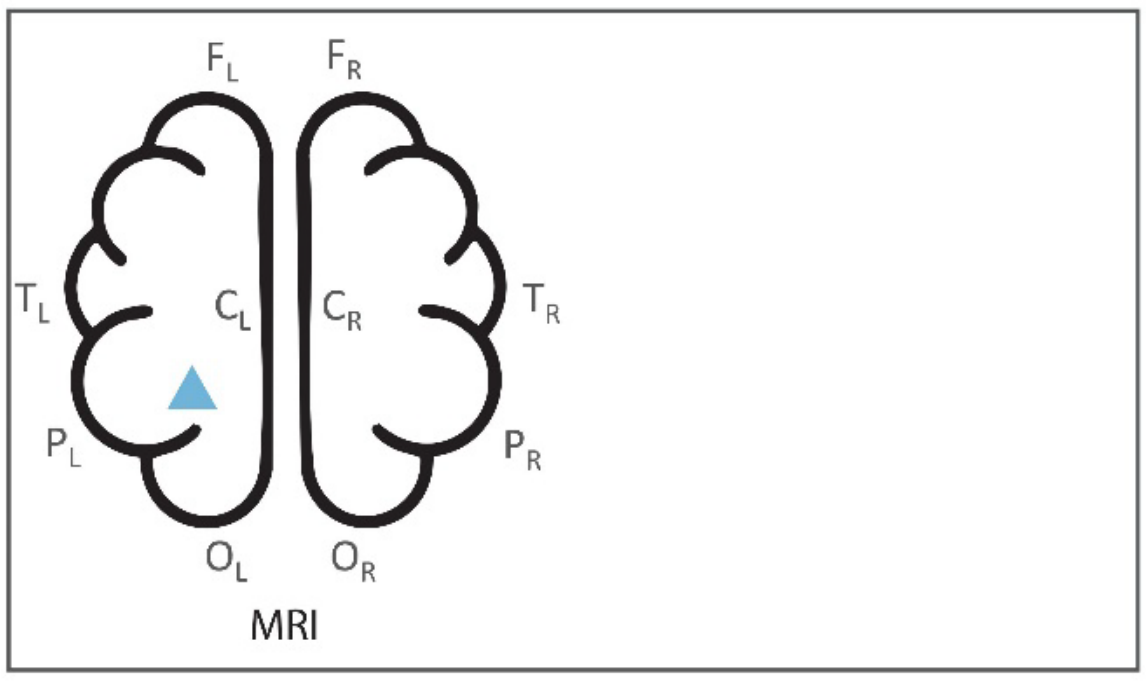

ictal	multifocal changing otherwise
interictal	interictal multifocality > ictal source(s)

patient 39

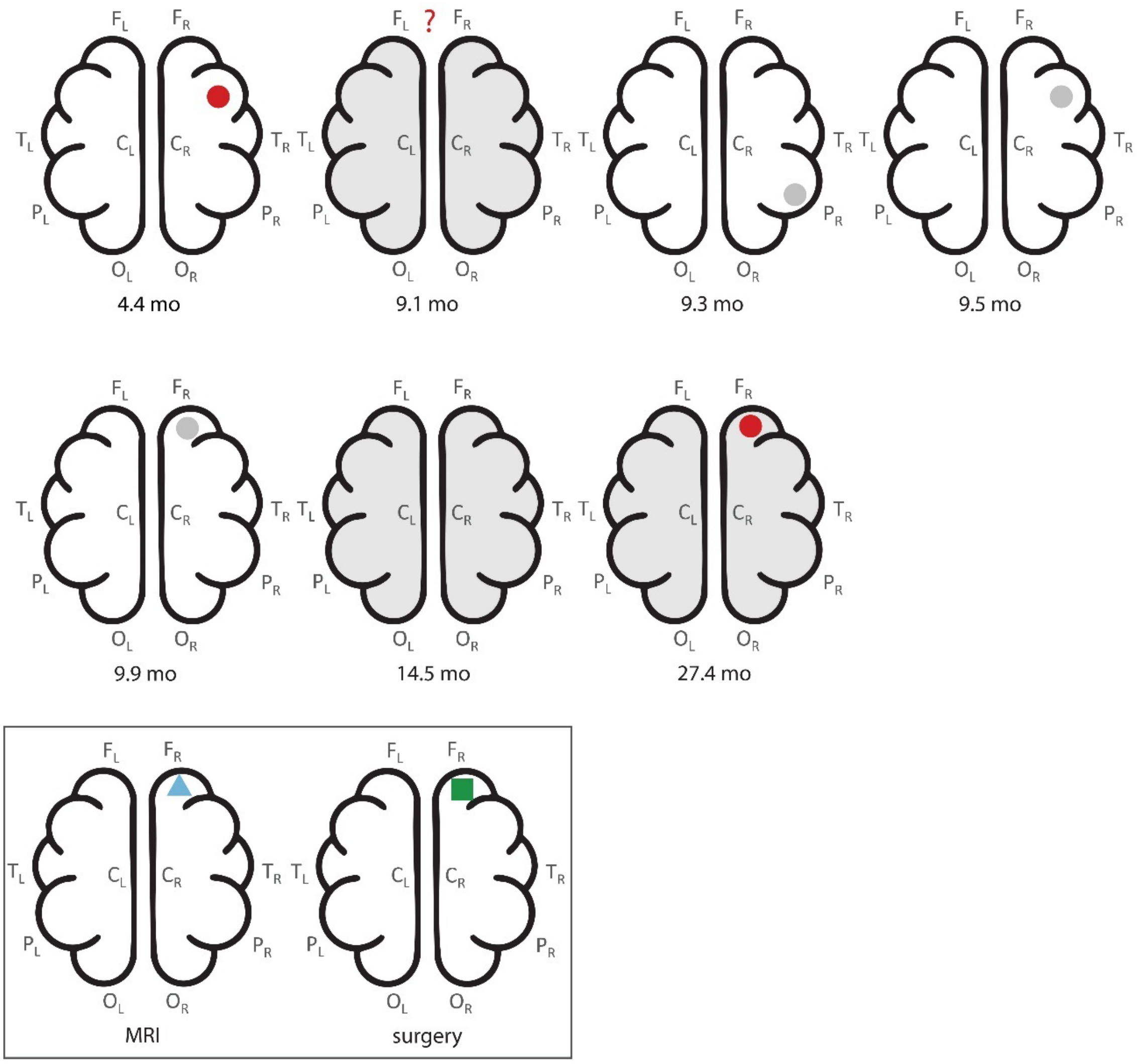

ictal	2 or more foci of which at least 1 is consistent
interictal	interictal multifocality > ictal source(s)

patient 40

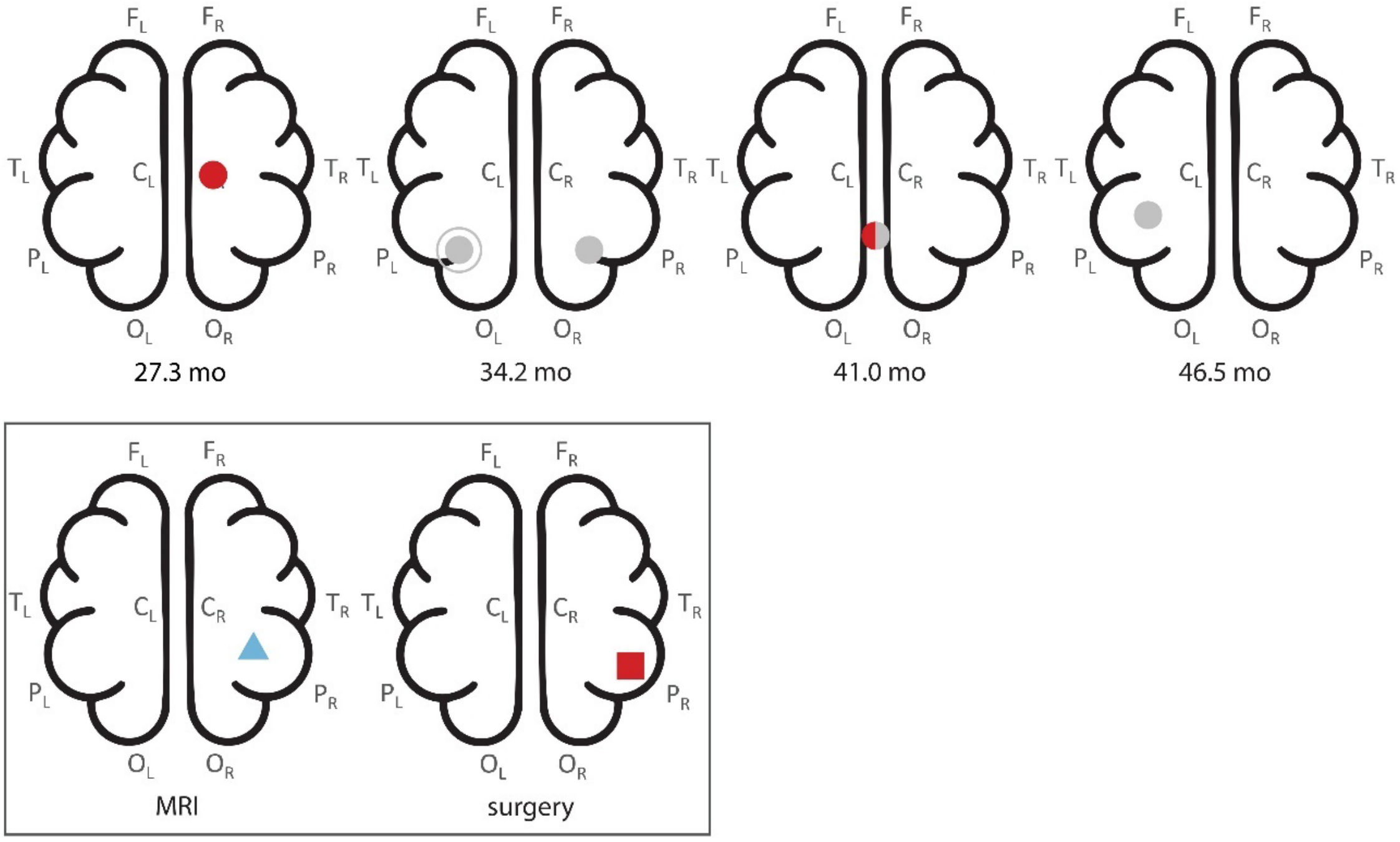

ictal	multifocal changing otherwise
interictal	interictal multifocality > ictal source(s)

patient 41

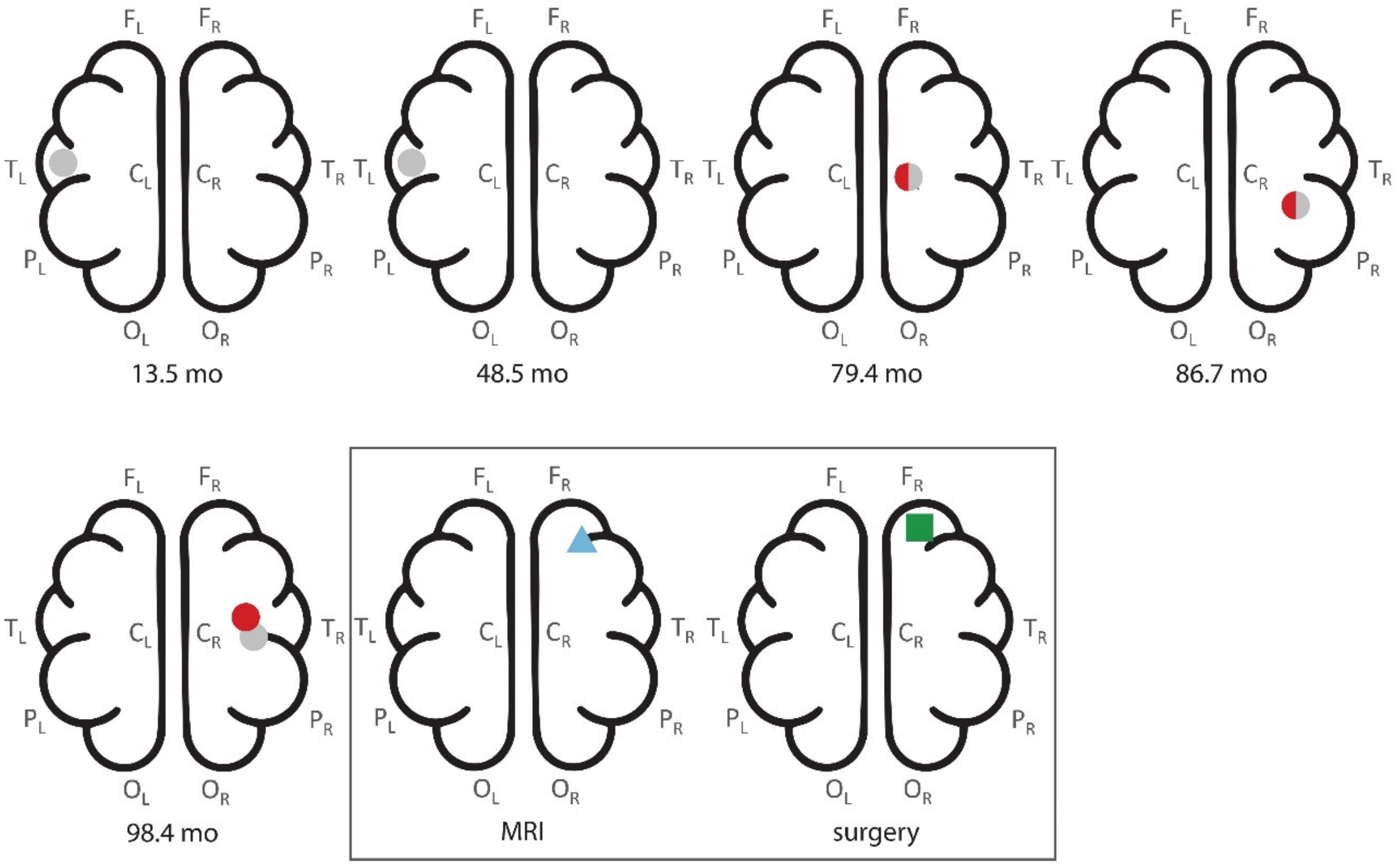

ictal	single focus, consistent
interictal	interictal multifocality > ictal source(s)

patient 42

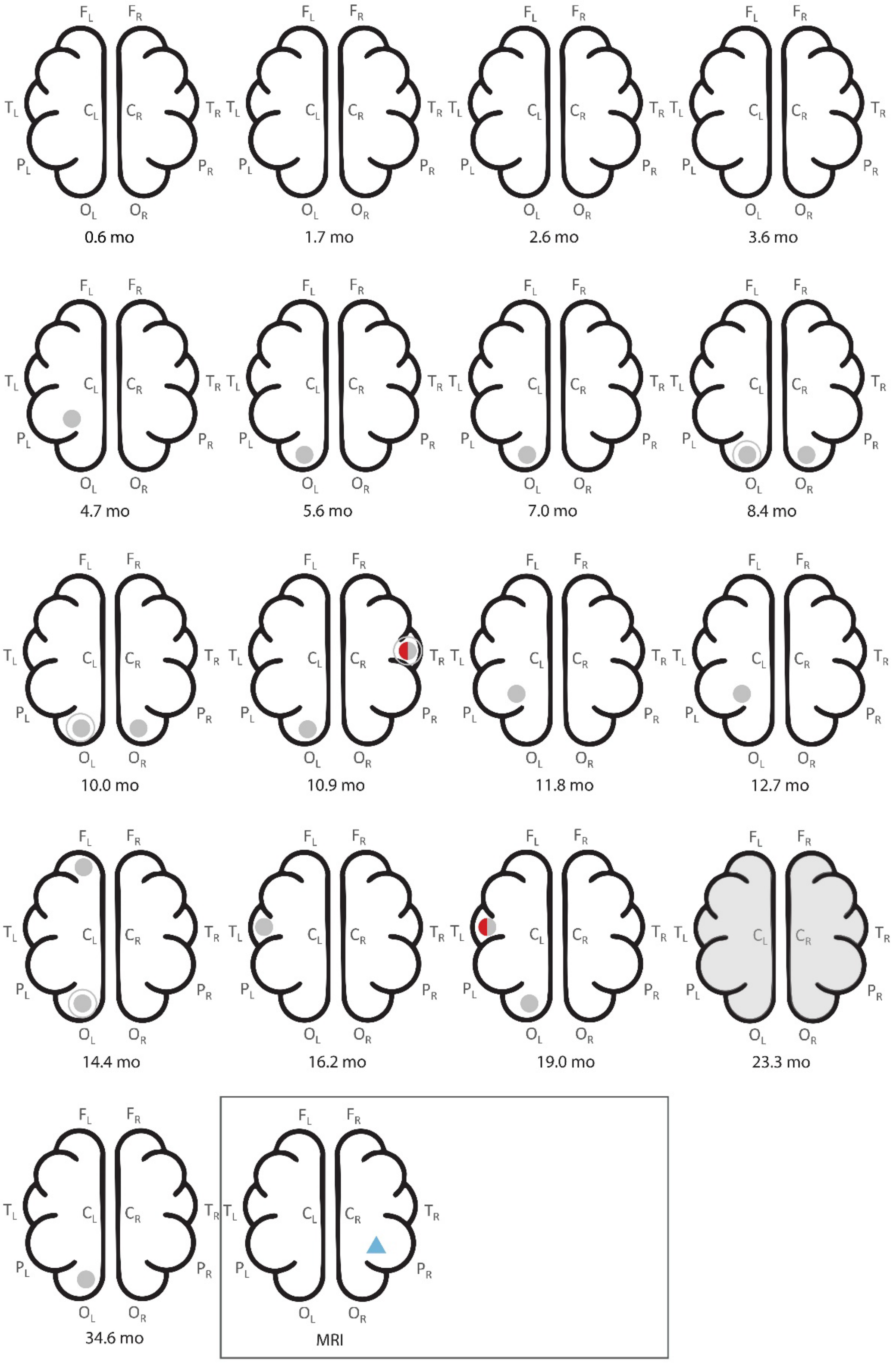

ictal	multifocal changing otherwise
interictal	interictal multifocality > ictal source(s)

patient 43

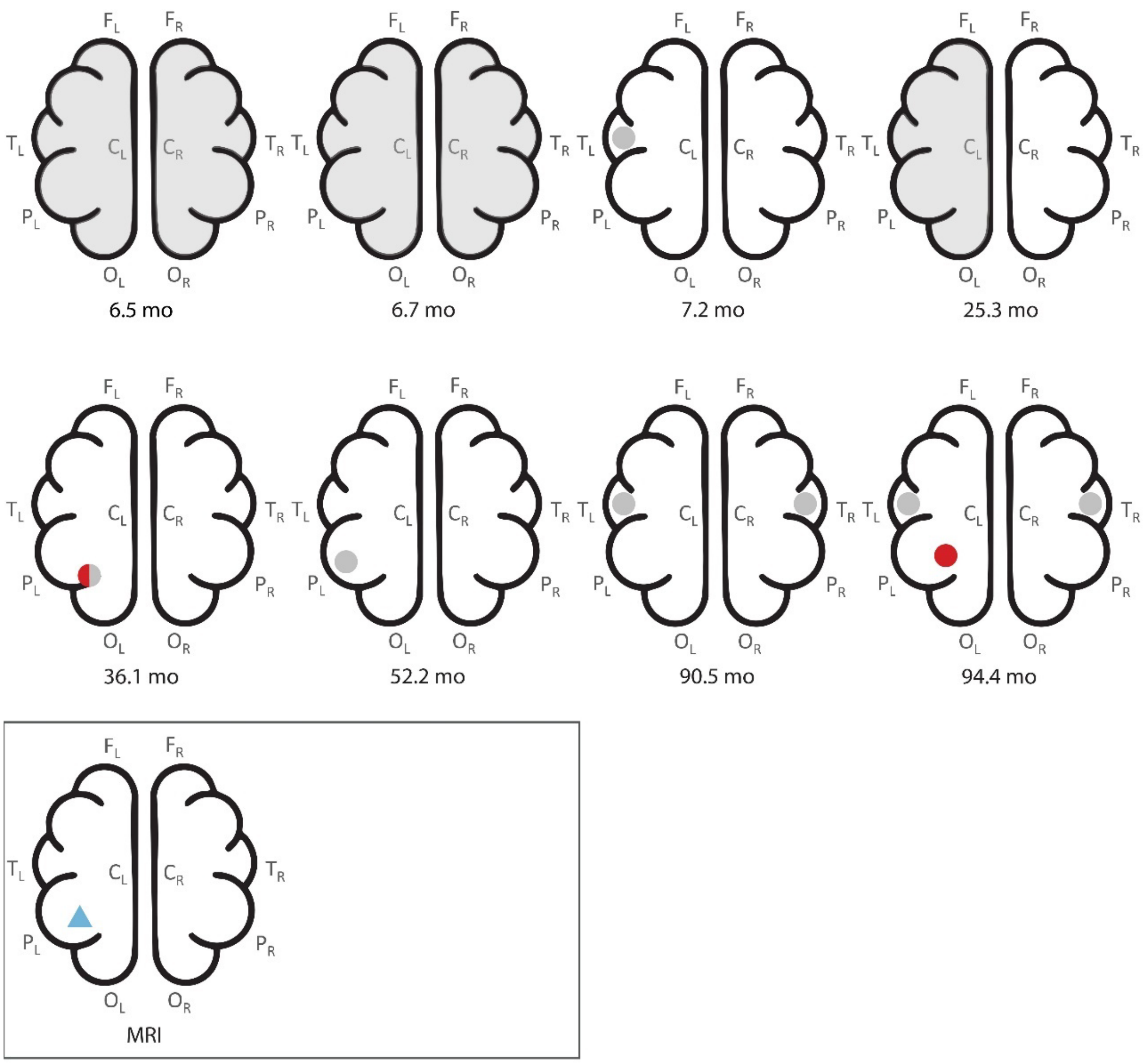

ictal	single focus, consistent
interictal	interictal multifocality > ictal source(s)

patient 44

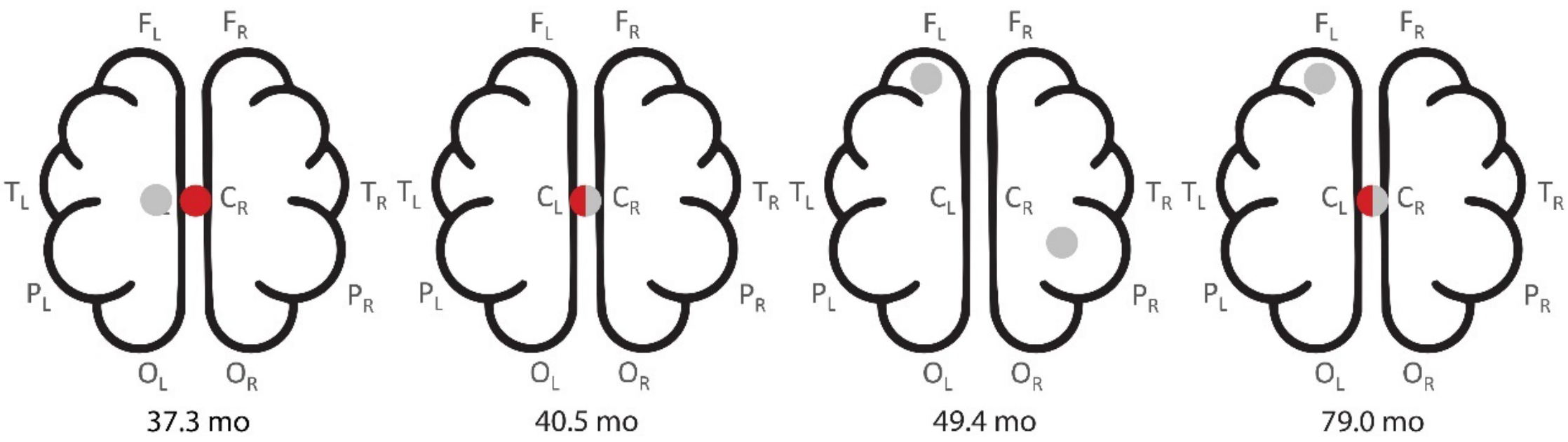

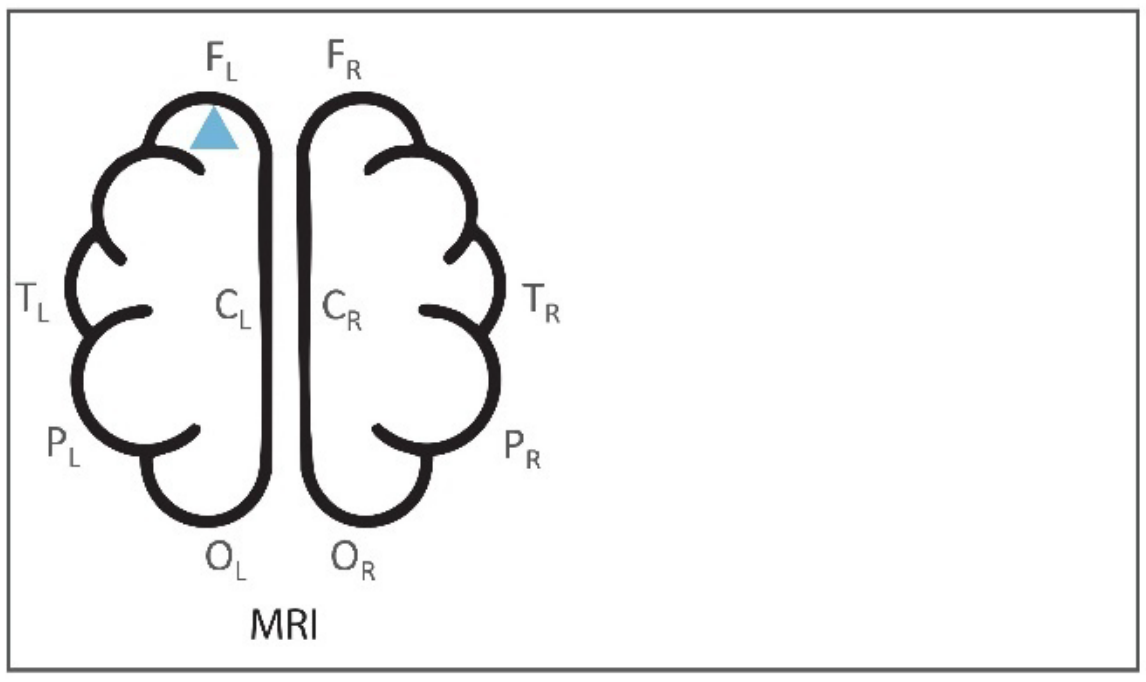

ictal	single focus, consistent
interictal	interictal multifocality > ictal source(s)

patient 45

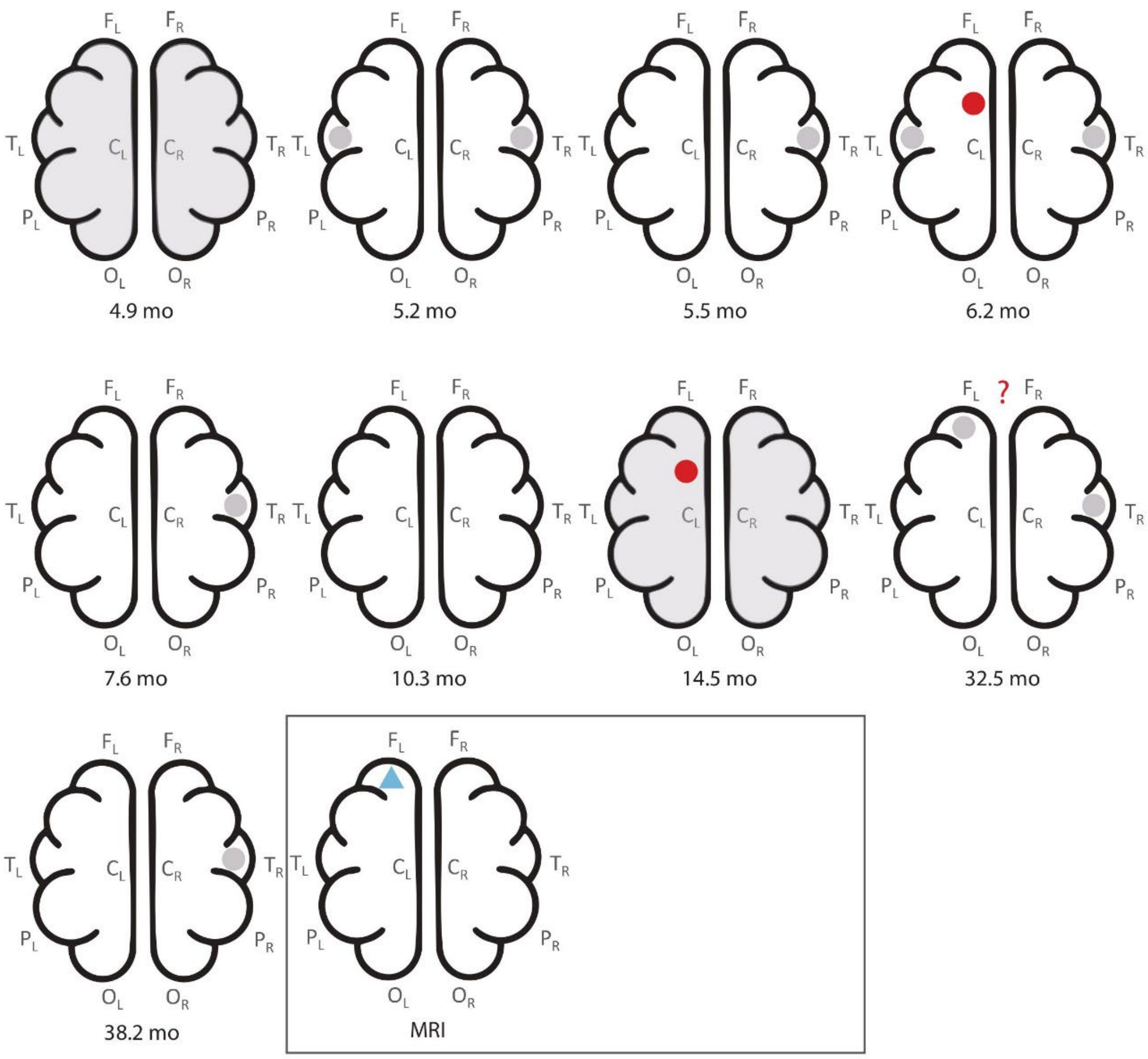

ictal	2 or more foci of which at least 1 is consistent
interictal	interictal multifocality > ictal source(s)

Supplementary Table S1. Distribution of determinants of consistent ictal epileptogenic foci by monitoring duration

	routine monitoring (n=8)	prolonged monitoring (n=37)
TSC 2 mutation*	6 (75.0)	25 (67.6)
age at onset of epilepsy (months)	3.1 (1.4 – 5.6)	4.3 (2.5 – 9.0)
history of epileptic spasms	3 (37.5)	27 (73.0)
multiple seizure types†	4 (50.0)	26 (70.3)
multifocality on interictal EEG‡	5 (62.5)	33 (89.2)
intermediate or high tuber load§	6 (75.0)	26 (70.3)
outcome: consistent ictal epileptogenic foci	4 (50.0)	7 (18.9)

Legend Determinants of interest are presented separately for participants with routine and prolonged EEG monitoring. Data are shown as number (percentage) within each stratum. The prevalence of consistent ictal epileptogenic foci within each monitoring category is also provided. Continuous variables are depicted as means (±SD), or medians (IQR: 25th percentile – 75th percentile) if not normally distributed, and count variables as n (%). SD = standard deviation. IQR = interquartile range. n = number of patients or EEGs. TSC = tuberous sclerosis complex. EEG = electroencephalogram. * = TSC mutation type was dichotomised to TSC 2 mutation type, and TSC 1 mutation type/mutation type not identified/not tested. † = dichotomous variable defined as multiple seizure types with seizure semiology localizing to different epileptogenic zones. ‡ = multifocality on interictal EEG ever during study period. § = intermediate and high tuber load were defined as respectively 10-20 and >20 tubers.
